# Genome-Wide Analysis Reveals Copy Number Variation-Mediated Adaptive Evolution in Chickens Under Diverse Environmental and Domestication Pressures

**DOI:** 10.3390/ani16213424

**Published:** 2026-10-29

**Authors:** Yufei Song, Linran Chen, Wenqing Lu, Yixin Luo, Naiyi Xu, Haiwei Wang, Guangxin E

**Affiliations:** 1 College of Animal Science and Technology, Southwest University, Chongqing 400715, China; songyufei2332005@163.com (Y.S.); chen.linran0413@outlook.com (L.C.); luwenqing0222@163.com (W.L.); luoyixin209@163.com (Y.L.); naiyixu@126.com (N.X.); 2 Chongqing Academy of Animal Sciences, Rongchang, Chongqing 402460, China

**Keywords:** chicken, copy number variation (CNV), selection signatures, thermal adaptation, high-altitude adaptation, domestication

## Abstract

To elucidate the genetic basis underlying chicken adaptation to diverse environmental selection pressures, this study performed a genome-wide analysis of copy number variations (CNVs) using publicly available whole-genome resequencing data from global chicken populations. Population differentiation (FST) and nucleotide diversity (π ratio) were integrated to identify signatures of selection associated with thermal adaptation, domestication, and high-altitude adaptation, followed by the identification of candidate genes and enriched biological pathways. In total, 6862, 9348, and 9384 high-confidence copy number variations (CNVs) were identified in the heat adaptation group, domestication group, and high-altitude adaptation group, respectively. Candidate genes associated with heat adaptation include *AQP9*, *SLC4A8*, *CAT*, *SMPD2*, and *CLOCK*, which are mainly enriched in biological processes related to water and ion homeostasis, antioxidant defense, and circadian rhythm regulation. Candidate genes related to domestication, such as *IL18R1*, *HTR2C*, *GRIN2A*, *PRKAG2*, and *FABP1*, are mainly involved in neuroactive ligand-receptor interaction, energy metabolism, immune regulation, and melanogenesis. In contrast, candidate genes identified in high-altitude populations include *EDNRB*, *ITPR1*, *OGDH*, *PPARA*, and *ADCY6*, which are significantly enriched in vascular smooth muscle contraction, the tricarboxylic acid (TCA) cycle, the PPAR signaling pathway, and pathways related to hypoxic metabolic adaptation. The above findings indicate that distinct environmental selection pressures and artificial selection pressures have shaped population-specific CNV patterns in the chicken genome and promoted the formation of phenotypic adaptation through regulating water–salt balance, neural function, energy metabolism, vascular homeostasis, and hypoxic response pathways.

## Simple Summary

Chickens have successfully adapted to diverse climates and artificial breeding environments worldwide, but the genetic mechanisms driven by copy number variations (CNVs) during environmental adaptation and domestication remain unclear. In this study, we systematically analyzed genome-wide CNVs using resequencing data from 590 chicken samples representing global populations. We compared three selection scenarios: high-temperature adaptation, high-altitude hypoxia adaptation, and artificial domestication. We identified numerous candidate CNV regions and functional genes that underwent significant positive selection. Our results showed that different adaptive environments shaped unique CNV patterns in the chicken genome. Key genes participated in water–ion balance, antioxidant defense, and circadian regulation for heat adaptation; vascular homeostasis and hypoxic metabolism for high-altitude adaptation; and neural regulation, energy metabolism, and immune remodeling for domestication. This study reveals the important role of CNVs in chicken adaptive evolution, provides a novel genetic perspective for understanding environmental adaptation and domestication, and provides candidate genomic regions that may inform future breeding after validation.

## 1. Introduction

The domestic chicken (*Gallus gallus domesticus*) is the most widely distributed domestic animal with the largest rearing population globally, and constitutes one of the most essential sources of animal protein for human consumption [1,2]. Through long-term natural selection and artificial breeding, domestic chickens have developed abundant genetic diversity, enabling them to adapt extensively to diverse ecological environments including tropical, temperate, high-cold, and high-altitude regions. Furthermore, they have evolved complex phenotypes associated with heat tolerance, cold tolerance, hypoxic adaptation, as well as domestication-related behaviors, growth, and reproduction [3,4]. With the intensification of global climate change and the rapid development of the modern poultry industry, environmental stresses such as high temperature and low oxygen have become key factors constraining the production performance and health status of poultry [5,6]. Therefore, dissecting the genetic mechanisms underlying environmental adaptation and domestication in domestic chickens will not only facilitate the elucidation of the adaptive evolutionary patterns of domestic chickens, but also provide a theoretical basis for the breeding of new stress-resistant varieties and the utilization of excellent genetic resources.

In recent years, with the extensive application of whole-genome resequencing technology, the genetic basis of adaptive evolution in domestic chickens has been extensively investigated. With regard to temperature adaptation, extensive analysis of selection signals across the whole genome indicates that genes associated with heat tolerance are primarily involved in biological processes including neuroendocrine regulation, energy metabolism, ion transport, and cardiovascular function. Specifically, *TSHR*, *FABP2*, *RAMP3*, *CAMK2D*, *ATP1A1*, *PLCB4*, *RYR2*, and *AKT3* exhibit significant selection signals in tropical chicken populations. These genes jointly participate in calcium signal transduction, thyroid hormone regulation, and cardiovascular homeostasis maintenance, forming a critical molecular network underlying heat tolerance adaptation in domestic chickens [7,8,9]. In contrast, adaptation to cold environments is more closely associated with processes such as mitochondrial energy metabolism, lipid metabolism, and myocardial contraction. For example, the Nixi chicken, a typical indigenous breed from the Yunnan-Guizhou Plateau, has been shown to possess high genetic diversity related to high-altitude tolerance [10]. Genes including *DNAH5*, *PTGS2*, *INHBA*, *IRX2*, and *NDUFS4* are recognized to be closely correlated with cold environment adaptation [11].

In addition to temperature, a typical characteristic of high-altitude regions is that organisms have long been exposed to multiple selective pressures including hypoxia, low temperature, and strong ultraviolet radiation. For this reason, high-altitude regions serve as an important model system for investigating adaptive evolution in response to environmental challenges. Existing studies have shown that through long-term natural selection, organisms have formed adaptive mechanisms centered on hypoxia perception, vascular remodeling, energy metabolism, and oxidative stress regulation [12]. Genes such as *AGTR1*, *ADAMTS7*, *CACNA1D*, *TPK1*, *BAZ2B*, *MARCHF7*, and *PARK7* have been continuously subjected to natural selection in multiple plateau chicken populations. Among these genes, functional verification has confirmed that *PARK7* can significantly improve the survival ability of cardiomyocytes under hypoxic conditions and plays a key protective role in the process of high-altitude adaptation [13,14,15].

Furthermore, chicken domestication is a complex evolutionary process gradually shaped by long-term artificial selection. This process not only alters the behavioral characteristics of domestic chickens, but also remodels their growth and development, reproductive capacity, immune function, and metabolic regulatory networks [16,17]. Current studies have identified that genes such as *TSHR*, *ALX1*, and *NTS* are closely associated with behavioral domestication, reproductive traits, and craniofacial development, while genes including *VSTM2A* and *RYR2* exhibit clear signatures of artificial selection [18]. In particular, genes related to neurodevelopment and reproduction such as *NRG3*, *NCAM2*, and *VTG2* show significant copy number variations between domestic chickens and red junglefowls, indicating that structural variations may play critical roles in the domestication process of chickens [19].

Copy number variations (CNVs) have been proven to alter gene dosage and structure through the deletion, duplication, or amplification of DNA fragments, exerting a crucial influence on gene expression and the formation of complex traits [20,21,22]. In recent years, in addition to single nucleotide polymorphisms (SNPs), CNVs have also been regarded as a key genetic factor driving phenotypic variation and adaptive evolution [23]. A large number of existing studies have confirmed that CNVs are widely involved in biological processes such as animal growth and development, environmental adaptation, and breed formation, playing an important role in the domestication of chickens and the formation of economic traits [24,25]. However, current research on chicken CNVs mainly focuses on a single environment or a single trait. The adaptive evolutionary mechanisms mediated by CNVs under different ecological conditions and under artificial selection pressure still lack systematic comparative analysis, and large-scale comprehensive studies based on multiple global geographical populations are also insufficient.

To fill this gap, this study utilized the public whole-genome resequencing data of 590 domestic chicken samples to systematically identify copy number variations (CNVs) throughout the chicken genome. Combined with whole-genome selection trait analysis, we identified candidate CNVs, functional genes, and biological pathways related to temperature adaptation, artificial domestication, and high-altitude hypoxic adaptation. The main new finding of this study is that different environmental pressures and artificial selection have promoted the formation and enrichment of CNVs in specific lineages, and these CNVs are closely related to key adaptive processes such as water–salt balance, oxygen metabolism, vascular homeostasis, immune regulation, neural regulation, and energy metabolism. From a breeding perspective, these results provide genome-wide CNV resources for molecular-assisted selection and genomic selection, facilitating the prioritized selection of genes that can be applied to the breeding of heat-tolerant, low-oxygen-tolerant, and more adaptable chicken strains, and contributing to the protection of chicken genetic resources. Moreover, these candidate CNVs and genes also provide testable hypotheses for future experimental verification.

## 2. Materials and Methods

### 2.1. Sample Collection and Data Preprocessing

A total of 590 publicly available whole-genome resequencing datasets of chickens were obtained, including 68 red junglefowl, 18 other junglefowl, 258 Chinese indigenous chickens, 55 chickens from Central and South Asia, 36 African chickens, 21 European chickens, 49 American chickens, 35 commercial lines, and 50 chickens from other regions (Table S1), covering wild red junglefowl, indigenous breeds, and commercial lines. Raw sequencing data were processed using SAMtools (v1.5) [26], and the main procedures included: sorting by chromosomal coordinates, constructing BAM indexes, removing PCR duplicates, and filtering low-quality bases with Phred quality scores lower than 20 and sequences contaminated by sequencing adapters. High-quality BAM files were finally obtained for subsequent variant detection.

### 2.2. Whole-Genome Copy Number Variation Detection

Whole-genome CNV detection was performed using CNVcaller (v1.3), a population-based software [27]. First, the whole genome was divided into overlapping sliding windows of 400 bp; the effective sequencing depth of all samples in each window was counted, and blank windows and abnormal windows with extremely low depth were eliminated. Next, absolute copy number correction and standardization were performed based on the global sequencing depth distribution to eliminate deviations in sequencing depth and GC content among samples. Then, the Pearson correlation coefficient between adjacent windows was calculated, and highly correlated windows with correlation coefficients no less than 0.4 were merged into a copy number variation region (CNVR). The copy-number state of each CNVR was assigned by comparing its normalized sequencing depth against the average normalized depth of diploid genomic regions: regions with normalized depth below this value were classified as copy-number loss, while those with normalized depth above this value were classified as copy-number gain. The raw CNV results were exported in VCF format and processed using VCFtools (v0.1.16) [28]. For each CNVR, different copy-number states were treated as alleles (e.g., CN0, CN1, CN2, and CN3 for losses, normal state, and gains), and allele frequencies were calculated. MAF was defined as the frequency of the least common copy-number allele at each CNVR. CNVRs were then strictly filtered, and only those longer than 2 kb with an MAF of at least 0.05 were retained to reduce false positives. Thus, an MAF threshold of 0.05 means that the minor copy-number allele has a population frequency of at least 5%, rather than the proportion of individuals carrying a CNV event. A total of 66,437 high-confidence autosomal CNVRs were finally obtained for subsequent analysis. The 400-bp window size was adopted following the recommendation of CNVcaller (v1.3) [27]. The Pearson correlation threshold of 0.4 for merging adjacent windows and the minimum CNVR length of 2 kb were selected according to widely used filtering criteria in livestock CNV studies using CNVcaller, balancing detection sensitivity and false-positive rate. As a computational cross-check, the identified CNVRs were compared with previously reported chicken CNVs. The lack of experimental validation is acknowledged as a limitation.

### 2.3. Grouping Design and Genome-Wide Selection Signature Analysis

To identify positive selection loci associated with temperature adaptation, artificial domestication, and high-altitude hypoxia adaptation, three independent comparative groups were designed. Temperature adaptation group: The target group consisted of chicken breeds adapted to long-term habitation in high-temperature regions, including Huiyang Beared Chicken, Xinghua Chicken, Heike Duji, Local chicken breeds of Ethiopia, Qiandongnan Mini Fragrant Chicken, Silkie, Fayoumi chicken, and Wenchang Chicken, while the control group included breeds from high-latitude, low-temperature regions including Korean native chicken, Norwegian Jaerhon, Orloff chicken, Jining Bairi Chicken, Lindian chicken, Beijing You Chicken, and Luxi Fighting Chicken (Table S2). Domesticated group: The long-term artificially selected indigenous chicken breeds including Xianju Chicken, Jining Bairi Chicken, Lindian chicken, Hetian Chicken, Lijiang Chicken, Weining chicken, Qiandongnan Small Fragrant Chicken, and Tibetan chicken were selected as the target population, with the wild subspecies of *Gallus gallus*, including Red Junglefowl, Hongyuan *Podiceps cristatus* subspecies, *Gallus gallus murghi*, *Gallus gallus gallus*, and *Gallus gallus spadiceus*, as the control population (Table S3). High-altitude adaptation group: Target populations consist of chicken breeds inhabiting plateaus above 2500 m above sea level including Lijiang Chicken, Nyingchi Chicken, Tibetan Black Chicken, Tibetan chicken, Shannan Chicken, Nixi chicken, Chahua Chicken, Wumeng Black-bone Chicken, Lhasa White Chicken, Weining chicken, Gongbo’gyamda Chicken, while control populations include chicken breeds from plains and low-altitude hilly areas including Yunyang Large Chicken, Beijing You Chicken, Xianju Chicken, Rhode Island Red chicken, Liyang chicken, Baier Yellow Chicken, Silkie, Luxi Gamefowl, Leghorn, Huiyang Bearded Chicken, Jining Bairi Chicken, White Leghorn, Wenchang Chicken, Xinghua Chicken, White Recessive Rock Chicken, and Sichuan low altitude chicken (Table S4).

Genome-wide selection signal analysis was performed using VCFtools (v0.1.16) to calculate the population differentiation index (FST) and nucleotide diversity ratio (π ratio) for each comparison group via a sliding window model (40 kb window size, 20 kb step size). Within each comparison group, all genomic windows were ranked independently in descending order based on FST and π ratio, respectively. The top 5% of windows for each statistic were extracted based on the empirical distribution of that statistic within the corresponding comparison group, equivalent to the 95th percentile. The overlap between FST and π ratio was evaluated at the genomic window level, not at the CNVR level. Because both statistics were calculated using the same set of sliding windows, each window was either included in both top 5% sets or not; therefore, no partial overlap between FST and π windows occurred. A window present in both top 5% sets was defined as a joint selection signal. Overlapping or adjacent joint-signal windows were merged into continuous candidate selection intervals by taking their union. If CNVR overlap was performed, these candidate selection intervals were then overlapped with the CNVRs detected by CNVcaller. A CNVR was considered a high-confidence candidate CNVR if it overlapped a candidate selection interval by at least 1 bp (or ≥50% of its length). Partially overlapping CNVRs were not merged, and each CNVR was evaluated independently. The group-specific thresholds were as follows: for the temperature group, FST > 0.038 and π ratio > 1.979; for the domestication group, FST > 0.034 and π ratio > 1.700; for the high-altitude group, FST > 0.051 and π ratio > 1.474.

Gene annotation of candidate CNVRs was completed via Shell script alignment against the GFF annotation file of the chicken reference genome GRCg7b, to accurately identify coding genes overlapping with CNVRs and located in the upstream and downstream flanking regions of CNVRs. Genes located within 2 kb upstream and downstream of CNVRs were included. All candidate CNVRs were annotated using ANNOVAR against the GRCg7b reference genome. The Shell-script alignment was used for initial filtering, and ANNOVAR was used for final annotation. For each comparison group, unique candidate genes annotated from the candidate CNVRs were used as the input gene list. The background gene set was all annotated Gallus gallus genes in the GRCg7b reference genome. Functional enrichment analysis was conducted using the online platform KOBAS 2.0 [29] based on the GO and KEGG databases. Fisher’s exact test was applied, and *p* < 0.05 was set as the threshold for significant enrichment to screen for gene functions and metabolic pathways significantly associated with adaptive traits.

## 3. Results

### 3.1. Overall Distribution Characteristics of Genome-Wide CNVRs

**Figure 1:**
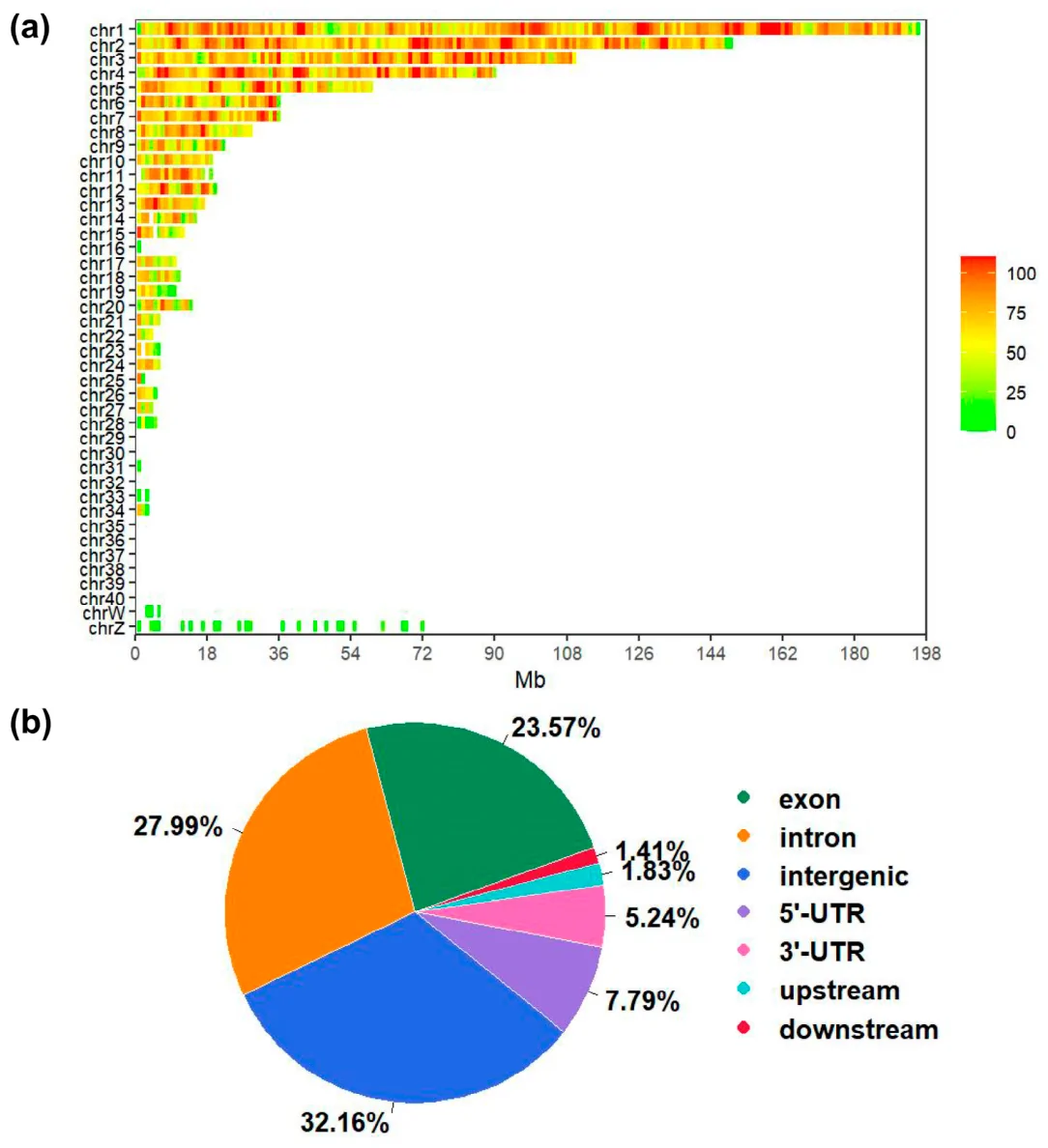
Chromosomal distribution of copy number variation (CNV) (**a**): Chromosomal distribution of 66,437 copy number variation regions across chicken breeds. The vertical axis indicates chromosome names, the horizontal axis represents chromosomal position in units of megabases (Mb), and the size of the colored regions corresponds to the length of copy number variation regions. (**b**): Pie chart showing the annotation proportions of copy number variation regions (CNVRs) across different functional genomic elements, including exons, introns, intergenic regions, 5′ untranslated regions (5′-UTR), 3′ untranslated regions (3′-UTR), and upstream/downstream regions of genes.

Genomic localization and annotation were performed on the high-confidence CNVRs obtained through screening. The chromosomal distribution of CNVRs is shown in Figure 1a. Among these regions, CNVRs exhibited the highest proportion of distribution in intergenic regions (32.16%), followed by intronic regions (27.99%) and exonic regions (23.57%). In regulatory and untranslated regions, the distribution proportions of 5′-UTR, 3′-UTR, and upstream and downstream regions of genes were 7.79%, 5.24%, 1.83%, and 1.41%, respectively (Figure 1b). These results indicate that chicken genomic structural variations widely cover coding regions, intergenic regions, and regulatory regions, providing an abundant basis for functional variation.

### 3.2. Analysis of CNVs and Candidate Genes in the Temperature Adaptation Group

**Figure 2:**
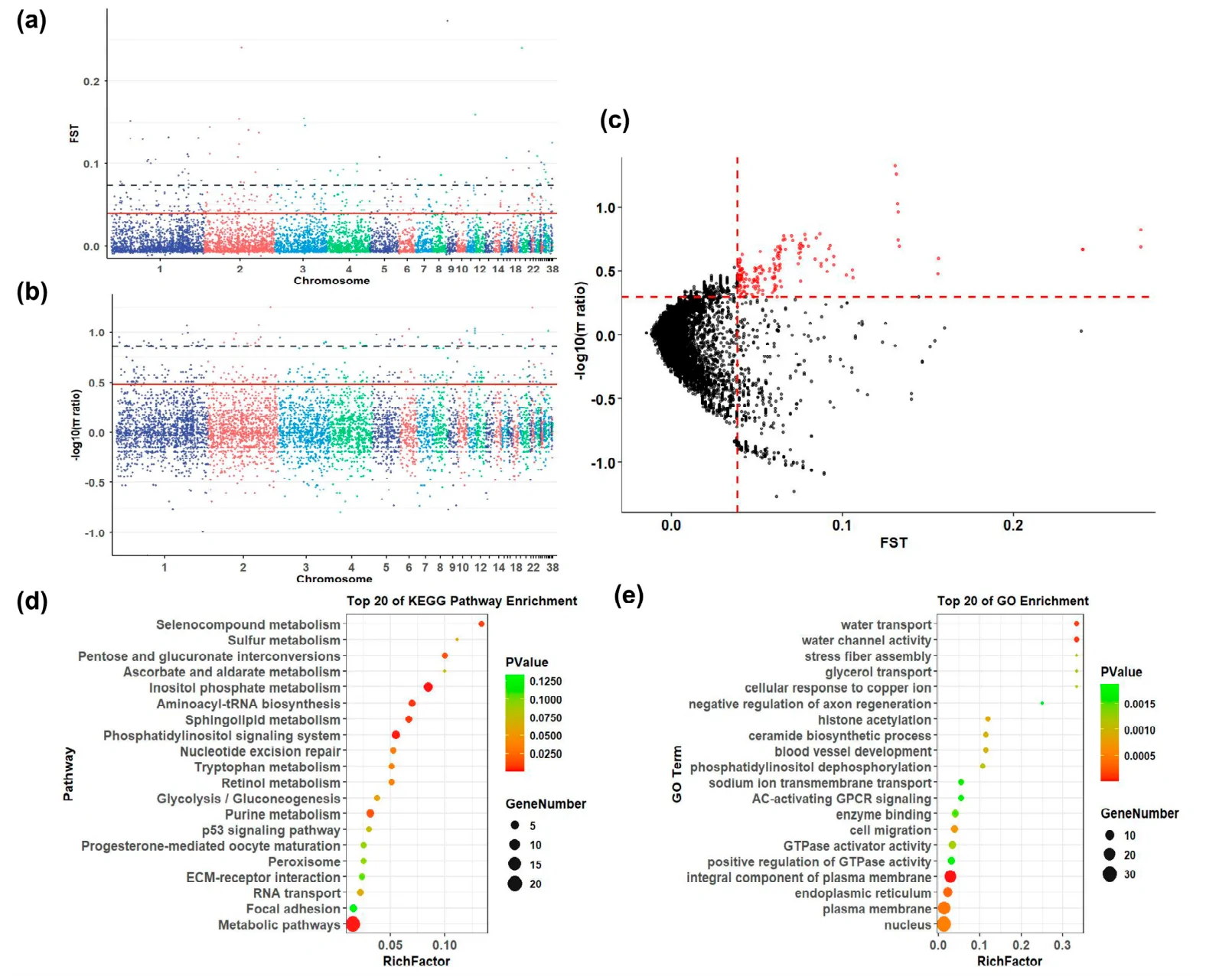
Genome-wide selection signature analysis and functional enrichment of candidate genes in temperature-adapted chickens. (**a**) Genome-wide distribution of FST on autosomes calculated using a 40 kb sliding window with a 20 kb step size; (**b**) Genome-wide distribution of π ratio on autosomes calculated using a 40 kb sliding window with a 20 kb step size; (**c**) Genome-wide distribution of the top 5% overlapping selection signature windows from FST and π ratio analyses, corresponding to high-confidence candidate CNVRs; (**d**) The top 20 significantly enriched KEGG terms screened from candidate genes; (**e**) The top 20 significantly enriched GO signaling pathways screened from candidate genes.

A total of 6862 reliable autosomal CNVs were identified across all data in the temperature adaptation group. The genome-wide distributions of FST and π ratio are presented in Figure 2a and Figure 2b, respectively. This study employed a combined screening approach using both FST and π ratio to detect candidate selection signals, with the top 5% of FST values (FST > 0.038) as the threshold. In the temperature-comparative populations, 546 selected CNV regions were identified, annotated to 373 coding genes including *CELF2*, *IL17REL*, and *ZNF800*. Additionally, using the top 5% of π ratio values (π ratio > 1.979) as an independent criterion, 564 significant CNV loci were detected, annotated to 248 functional genes such as *CNKSR2*, *MAP7D2*, and *PUDP*. The intersection of these two screening methods yielded 271 selected CNV regions, corresponding to 199 candidate functional genes (e.g., *MAP7D2*, *SPRY2*, *DIAPH3*, *CNTN5*) (Table S5, Figure 2c).

The results of KEGG pathway enrichment analysis showed that a total of 50 genes were enriched in 68 KEGG pathways, among which 11 KEGG pathways were significantly enriched (*p* < 0.05), mainly including inositol phosphate metabolism, phosphatidylinositol signaling system, and aminoacyl-tRNA biosynthesis. The aforementioned pathways are widely involved in biological processes including cellular metabolic homeostasis, antioxidant defense, immune recognition, and DNA damage repair (Table S6, Figure 2d). The results of GO functional enrichment analysis showed that a total of 854 GO terms were enriched from 139 candidate genes, among which 128 candidate genes were significantly enriched in 164 GO terms. These enriched terms mainly included aquaporin activity, cell migration, histone acetylation, vascular development, and ceramide biosynthesis (Table S7, Figure 2e).

### 3.3. Analysis of CNVs and Candidate Genes in the High-Altitude Adaptation Group

**Figure 3:**
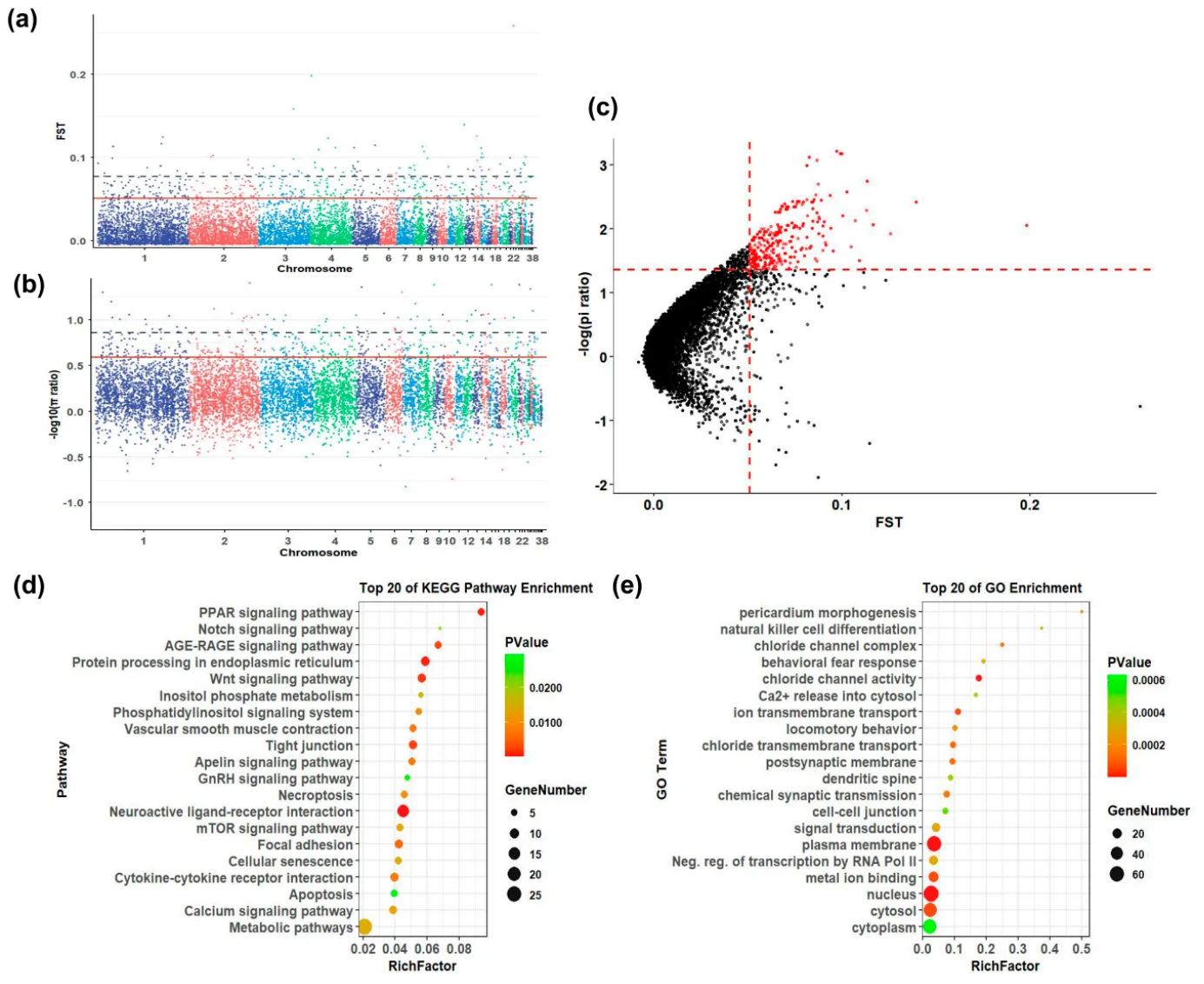
Genome-wide selection signal analysis of altitude-adapted chicken populations using the CNV dataset. (**a**) Genome-wide distribution of FST across autosomes calculated with a 40 kb sliding window (20 kb step size); (**b**) Genome-wide distribution of π ratio across autosomes calculated with a 40 kb sliding window (20 kb step size); (**c**) Genome-wide distribution of the intersection of the top 5% of selection signal windows from FST and π ratio analysis, corresponding to high-confidence candidate CNVRs; (**d**) The top 20 significantly enriched KEGG functional terms screened from candidate genes; (**e**) The top 20 significantly enriched GO signaling pathways screened from candidate genes.

A total of 9384 reliable autosomal copy number variations (CNVs) were identified in the high-altitude adaptation group. The genome-wide distributions of FST and π ratio are presented in Figure 3a and Figure 3b, respectively. With FST > 0.051 between high- and low-altitude populations set as the top 5% screening threshold, 712 significantly differentiated CNV regions were obtained, and 578 protein-coding genes, including *TMEM47*, *GREM2*, *FMN2,* and *NELL2,* were annotated. With π ratio > 1.474 set as the top 5% diversity screening threshold, 712 significant CNV loci were also obtained, and 598 functional genes, including *GNAI1*, *PHTF2*, *TMEM60*, and *RSBN1L*, were obtained through annotation screening. By taking the intersection interval of the two selection signals, 433 high-confidence CNV regions associated with high-altitude adaptation were finally obtained, and 373 candidate genes, including *PUDP*, *RSBN1L*, *GABRA5*, and *NPAS2*, were annotated in total (Table S8, Figure 3c).

KEGG enrichment analysis revealed that 123 candidate genes were collectively enriched in 100 pathways, with significant enrichment observed in 25 of these pathways, including neuroactive ligand–receptor interaction, protein processing in the endoplasmic reticulum, and the Wnt signaling pathway (Table S9, Figure 3d). GO annotation showed that 279 genes were enriched in 1459 functional terms, with 258 genes significantly enriched in 245 GO terms, primarily involving chloride channel activity, transmembrane transport of chloride ions, and chemical synaptic transmission (Table S10, Figure 3e).

Notably, this study also identified a panel of shared genes such as HTR2C, GRIA3, TNFRSF19, and NCOR2 that were subjected to selection in both temperature adaptation and altitude adaptation. These genes were mainly enriched in biological pathways including cytokine–cytokine receptor interaction, neuroactive ligand–receptor interaction, and the Notch signaling pathway.

### 3.4. Domestication Group CNV and Candidate Gene Analysis

**Figure 4:**
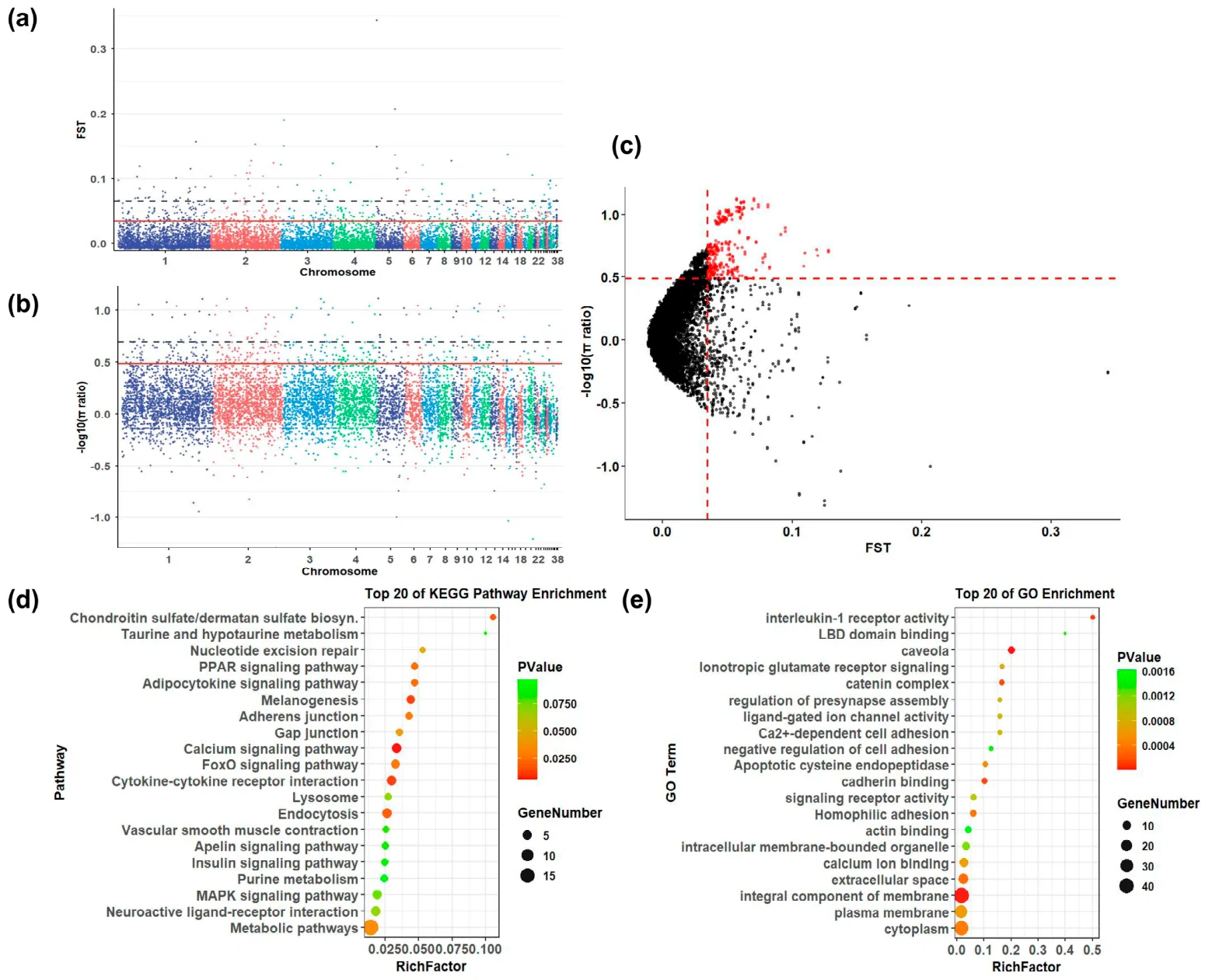
Genome-wide selection signal analysis of domesticated chickens using the CNV dataset. (**a**) Genomic distribution of FST on autosomes calculated via a 40 kb sliding window with a 20 kb step size; (**b**) Genomic distribution of π ratio on autosomes calculated via a 40 kb sliding window with a 20 kb step size; (**c**) Genome-wide distribution of the intersection of the top 5% selection signal windows from FST and π ratio (i.e., high-confidence candidate CNVRs); (**d**) The top 20 significantly enriched KEGG functional terms screened from candidate genes; (**e**) The top 20 significantly enriched GO signaling pathways screened from candidate genes.

A total of 9348 high-quality autosomal CNVs were detected in the domestication group. The genome-wide distributions of FST and π ratio are presented in Figure 4a and Figure 4b, respectively. Based on a selection threshold of FST > 0.034 for the top 5%, 699 significantly selected CNV regions were identified, successfully annotated to 570 functional genes, including *GRM8*, *FAM3C*, *WNT16*, and *CPED1*. Using the top 5% cutoff of π ratio > 1.700, 701 significant CNV loci were selected, annotating 912 genes such as *GABRG3*, *NPAS2*, and *RPL31*. Taking the intersection of the significantly differentiated regions from both FST and π ratio analyses, a final set of 371 high-confidence domestication-associated CNV regions was obtained, and 268 functional genes, including *FAM3C*, *IMMP2L*, *ACSS3*, and *CRADD*, were annotated (Table S11, Figure 4c).

The KEGG pathway enrichment analysis revealed that 75 candidate genes were enriched in a total of 85 KEGG pathways, among which 12 pathways were significantly enriched, including the calcium signaling pathway, melanogenesis, cytokine-cytokine receptor interaction, and other pathways. The aforementioned pathways are highly consistent with the behavioral traits, immune function, and phenotypic remodeling during the domestication process of domestic chickens (Table S12, Figure 4d). The GO enrichment results showed that 183 genes were enriched in 1103 functional terms in total, and 158 genes were significantly enriched in 162 GO terms, mainly including integral component of membrane, interleukin-1 receptor activity, cadherin binding, and cysteine-type endopeptidase activation (Table S13, Figure 4e).

## 4. Discussion

This study identified a total of 66,437 highly confident autosomal copy number variations (CNVRs). These CNVRs were widely distributed in intergenic regions, introns, exons, and regulatory regions, indicating that the CNVRs in chickens cover the coding regions, non-coding regions, and regulatory regions, providing a rich foundation for functional variations. This genomic distribution is helpful for interpreting the significance of candidate CNVRs in the process of adaptation and domestication in the future.

Heat stress severely suppresses poultry production performance by inducing dehydration, oxidative stress, and acid–base imbalance [30]. Massive loss of body fluid and CO_2_ induced by thermal polypnea can lead to respiratory alkalosis [31]. Existing studies have demonstrated that aquaporin 9 (*AQP9*) is expressed in the central nervous system, such as the hypothalamus. As an aquaglyceroporin, *AQP9* facilitates the transmembrane transport of water and glycerol, which indicates that *AQP9* may be involved in the regulation of water homeostasis in the central nervous system [32,33], while *SLC4A8* buffers neuronal acid–base disorder via sodium-dependent carbonate exchange [34]. It is therefore inferred that the synergistic action of these two genes can form a defensive line for water–salt and acid–base balance under heat stress. Furthermore, in the temperature adaptation group, the KEGG enrichment analysis revealed the pathways of inositol phosphate metabolism, phosphatidylinositol signaling system, and aminoacyl-tRNA biosynthesis. These pathways are extensively involved in cellular metabolic homeostasis, antioxidant defense, immune recognition, and DNA damage repair, which are consistent with the physiological challenges brought about by heat stress.

At the level of antioxidant defense and membrane homeostasis, heat stress induces massive accumulation of mitochondrial reactive oxygen species that exceeds the cellular clearance threshold [34,35,36]. Catalase (CAT) is a key antioxidant enzyme that catalyzes the decomposition of H_2_O_2_ to H_2_O and O_2_, thereby protecting cells against oxidative stress in chickens [37,38]. It has been confirmed that *SMPD2* catalyzes the hydrolysis of sphingomyelin to generate ceramide, and remodels membrane lipid composition to maintain the fluidity and integrity of cell membranes, potentially under high temperature [39,40,41].

The aforementioned peripheral defense is ultimately temporally integrated by the core circadian rhythm regulator *CLOCK* [42,43,44]. Existing studies have revealed that *CLOCK* is highly sensitive to intracellular H_2_O_2_ redox fluctuations; it can cooperate with *BMAL1* and *Nrf2* to drive the rhythmic expression of antioxidant genes [45], and bind to *HSF1* to induce heat shock proteins under heat shock conditions [46]. Collectively, these findings indicate that *AQP9*, *SLC4A8*, *CAT*, *SMPD2*, and the *CLOCK* gene may jointly delineate a complete molecular network of high-temperature adaptation ranging from heat dissipation homeostasis and antioxidant protection to neuroendocrine circadian integration.

Extensive theoretical studies on ecological adaptation have confirmed that the high-altitude hypoxic environment drives domestic chickens to form an adaptive mechanism centered on vascular remodeling and metabolic reprogramming [47,48,49]. Clinicopathological studies have established that hypoxia-induced excessive contraction and abnormal remodeling of pulmonary arteries can lead to pulmonary hypertension and right heart dysfunction [50,51]. In this study, ITPR1 was enriched in the calcium signaling pathway and the vascular smooth muscle contraction pathway. Previous studies have shown that ITPR1 mediates hypoxic pulmonary vascular contraction by regulating endoplasmic reticulum calcium release and optimizes the ventilation/perfusion ratio [52]. Similarly, EDNRB has been reported to establish an endogenous protective mechanism by promoting the release of nitric oxide (NO) and prostacyclin (PGI_2_), thereby inhibiting abnormal proliferation of vascular smooth muscle and preventing excessive vascular contraction [53,54,55]. We speculate that these two genes may work together to balance the vasoconstriction and vasodilation induced by hypoxia, thereby reducing the risk of cardiac injury at high altitudes.

Extensive studies have confirmed that oxidative phosphorylation is inhibited under hypoxic stress, and organisms rely on enhanced glycolysis to compensate for insufficient energy production via glucose oxidation [56,57,58]. *PPARA*, a core transcription factor that regulates lipid metabolism, can activate the expression of rate-limiting enzymes in the fatty acid β-oxidation pathway and increase the energy supply level from lipid catabolism [59,60]. Furthermore, the promoter region of the *ADCY6* gene contains a HIF-1 binding site; after *ADCY6* expression is upregulated under hypoxic conditions, it catalyzes the synthesis of cAMP and maintains the activity of the PKA pathway, thereby ensuring the normal progression of cell migration and metabolic adaptation [61]. The aforementioned studies indicate that a multi-level molecular regulatory network may exist during the adaptation of domestic chickens to high-altitude hypoxia.

Notably, this study further confirms that genes including *IL13RA2*, *TNFRSF19*, *GRIA3*, and *NCOR2* are subjected to positive selection in both the temperature-adapted group and the high-altitude-adapted group. The *GRIA3* gene encodes a subunit of the AMPA-type glutamate receptor, which mediates excitatory synaptic transmission in neurons [62,63]. Both heat stress and hypoxia can induce neuronal excitability remodeling, indicating that this gene may be involved in neuroprotection and adaptive regulation under the two stress conditions. *IL13RA2* and *TNFRSF19* belong to the interleukin receptor superfamily and the tumor necrosis factor receptor superfamily, respectively: in an IL-10-deficient colitis model, IL-13Rα2 attenuates the IL-13-mediated protective effect and thereby promotes intestinal inflammation driven by Th1/Th17 [64]; *TNFRSF19* is involved in immune evasion by inhibiting senescence-associated secretory phenotype-like features [65]. Both of these genes may function in maintaining immune homeostasis during hyperthermia-induced systemic inflammation and hypoxia-induced tissue damage. As a nuclear receptor co-repressor, *NCOR2* regulates the signaling pathways of nuclear receptors such as thyroid hormone receptors and *PPARs* to influence energy metabolism and adipocyte differentiation [66], and it may play a synergistic role in temperature adaptation and hypoxia-induced metabolic reprogramming.

Extreme temperatures and high-altitude environments pose significant challenges to the energy metabolism, temperature regulation, and oxidative stress defense of organisms. The discovery of common genes indicates that domestic chickens may have constructed a common molecular basis by repeatedly utilizing a set of core pleiotropic genes, providing a new perspective for understanding the co-evolution of domestic chickens in multi-dimensional environmental adaptation. In conclusion, the aforementioned shared genes indicate that temperature adaptation and high-altitude adaptation are not completely independent evolutionary processes, and domestic chickens can construct a common molecular basis for cross-stress adaptation by repeatedly recruiting a set of pleiotropic genes.

Furthermore, the academic community has reached a consensus: domestic chickens originated from the long-term artificial domestication of the wild red junglefowl (*Gallus gallus*), and exhibit typical characteristics of the domestication syndrome, including a docile temperament, changes in energy metabolism, and the phenomenon of diverse feather colors [67,68]. In the domesticated population, KEGG and GO enrichment analyses revealed pathways such as calcium signal transduction, melanin production, cytokine-cytokine receptor interactions, membrane integration components, interleukin-1 receptor activity, cadherin binding, and cysteine-type endopeptidase activation. These pathways are consistent with previous functional research results, namely that calcium signal transduction and melanin production are related to neural crest development and pigmentation, cytokine-cytokine receptor interactions and interleukin-1 receptor activity are related to immune regulation, and cadherin binding and cysteine-type endopeptidase activation are related to cell adhesion and protein hydrolysis involved in immunity. These findings collectively indicate that the candidate genes identified may be involved in the behavioral, immune, and phenotypic reshaping processes related to chicken domestication, rather than directly constituting its molecular basis. At the behavioral level, the HTR2C gene encodes the 5-hydroxytryptamine receptor, which is a key molecular switch regulating fear and aggressive behavior; structural variations of this gene can significantly reduce the organism’s sensitivity to environmental stress and reduce the occurrence of defensive aggression [69,70,71]. GRIN2A is an important subunit of the NMDA receptor, involved in regulating synaptic plasticity and central nervous system signal transduction [72]. Due to the reduction in fear and aggressive levels during domestication, the screening effects of HTR2C and GRIN2A may help explain the behavioral reshaping phenomenon exhibited by domestic chickens. The synergistic effect of these two genes, which decouples the “environmental stimulus—defense stress” neural pathway, suggests that this provides a neurobiological basis for redistributing energy from defensive behaviors to growth and development.

At the metabolic level, *PRKAG2* encodes the γ2 regulatory subunit of AMPK, which is a core factor for cellular energy sensing and can globally regulate glycogen metabolism and lipid synthesis [73,74]. A weakened stress response increases the body’s energy surplus and inhibits AMPK activity, thereby upregulating fatty acid uptake and deposition mediated by *FABP1*, promoting fat accumulation [75]. This is consistent with the energy metabolism changes and increased fat deposition observed during chicken domestication. Additionally, *WNT9B* is involved in melanin production and the development of neural crest cells. As a common precursor for the nervous system and pigment cells, the change in the differentiation fate of neural crest cells may lead to the synchronous appearance of diverse feather colors [76,77,78]. Therefore, *WNT9B* provides a reasonable link between neural crest development and feather color diversification, which is part of the chicken domestication syndrome. To adapt to possible inflammatory damage in high-density intensive farming environments, interleukin receptor genes such as *IL1RL1* and *IL18R1* can positively regulate inflammatory responses, macrophage activation, and antiviral immunity, providing a potential molecular basis for the adaptation of immune function during domestication [79,80,81,82]. In conclusion, we speculate that the *HTR2C–GRIN2A* behavioral module, *PRKAG2–FABP1* energy module, and *WNT9B*–immune gene module jointly construct a multi-dimensional phenotypic reshaping network supporting chicken domestication.

This study has several limitations. First, the candidate CNVs and genes were identified through whole-genome resequencing and bioinformatic prediction and were not experimentally validated. Second, CNV detection using CNVcaller is sensitive to sequencing depth and coverage; although we performed depth correction, standardization, and filtering, residual technical bias cannot be completely excluded. Third, population structure, sample size, and reference genome quality may influence CNV calling and selection signal detection. Fourth, the domestication comparison may capture demographic history, population bottlenecks, drift, and divergence in addition to artificial selection. Because FST and π are not independent of demographic processes, their intersection does not eliminate confounding by bottlenecks, founder effects, or population subdivision. Therefore, future studies should validate the prioritized CNVs and genes using qPCR, reporter assays, and gene-editing experiments, and evaluate their effects on heat tolerance, hypoxic adaptation, and production traits in chicken breeding programs.

## 5. Conclusions

As one of the most economically valuable livestock species globally, the domestic chicken (*Gallus gallus domesticus*) has undergone complex environmental adaptation and genetic remodeling through long-term natural selection and artificial domestication, resulting in rich genetic diversity and lineage-specific phenotypic traits. However, the genetic basis of its adaptive evolution remains incompletely elucidated, particularly under extreme ecological pressures such as heat stress and hypoxia, as well as in the context of trait improvement driven by artificial selection. In recent years, structural variants (SVs), especially copy number variations (CNVs), have been recognized as playing critical roles in animal environmental adaptation and artificial selection due to their broad impact on the genome. CNVs can influence biological traits by altering gene dosage, regulating gene expression, and modulating the function of functional genomic elements. Nevertheless, current research on chicken adaptive evolution has primarily focused on single nucleotide polymorphisms (SNPs) and selection signal analysis, while the systematic roles of copy number variations (CNVs) in temperature adaptation, high-altitude hypoxia adaptation, and artificial domestication processes remain unclear. Based on whole-genome resequencing data from chicken populations across multiple geographic regions worldwide, this study systematically identified the variation characteristics of CNVs under different ecological environments and domestication backgrounds, mined key CNV loci and candidate genes associated with heat tolerance, high-altitude adaptation, and domestication selection, and generated testable hypotheses about their potential functional mechanisms. The principal novel findings of this study are as follows: distinct environmental pressures and artificial selection drove the formation and enrichment of lineage-specific CNVs, which may contribute to chicken environmental adaptation and the formation of domesticated traits by regulating key biological processes including water–salt balance, oxygen metabolism, vascular homeostasis, immune regulation, neural regulation, and energy metabolism. From a breeding perspective, these results provide genome-wide copy number variation (CNV) resources and prioritize candidate loci and genes suitable for molecular-assisted selection and genomic selection, aiming to cultivate heat-tolerant, hypoxia-tolerant, and more adaptable chicken breeds, while also contributing to the conservation of domestic chicken genetic resources. Although these findings provide candidate CNVRs and generate testable hypotheses, they are based on bioinformatic prediction and require further experimental validation. Future studies using qPCR, reporter assays, and gene-editing approaches will be needed to confirm the functional roles of the prioritized CNVs and genes in chicken adaptation and breeding.

## Data Availability

Publicly available datasets were analyzed in this study. These data can be found in the DDBJ Sequence Read Archive (DDBJ DRA), the European Nucleotide Archive (ENA), and the NCBI Sequence Read Archive (NCBI SRA) under the accession numbers listed in Supplementary Table S1.

## Supplementary Materials

The following supporting information can be downloaded at: https://www.mdpi.com/article/10.3390/ani16213424/s1, Table S1: Sample information and geographic origins of the chicken populations used in this study; Table S2: Population grouping for the thermal adaptation analysis; Table S3: Population grouping for the domestication analysis; Table S4: Population grouping for the altitude adaptation analysis; Table S5: Candidate CNVRs and annotated genes under positive selection in the thermal adaptation group; Table S6: KEGG pathways significantly enriched for genes overlapping candidate CNVRs under thermal adaptation selection; Table S7: GO terms significantly enriched for genes overlapping candidate CNVRs under thermal adaptation selection; Table S8: Candidate CNVRs and annotated genes under positive selection in the high-altitude adaptation group; Table S9: KEGG pathways significantly enriched for genes overlapping candidate CNVRs under high-altitude adaptation selection; Table S10: GO terms significantly enriched for genes overlapping candidate CNVRs under high-altitude adaptation selection; Table S11: Candidate CNVRs and annotated genes under positive selection in the domestication group; Table S12: KEGG pathways significantly enriched for genes overlapping candidate CNVRs under domestication selection; Table S13: GO terms significantly enriched for genes overlapping candidate CNVRs under domestication selection.
