# Supplementary material for: Genome-Wide Analysis Reveals Copy Number Variation-Mediated Adaptive Evolution in Chickens Under Diverse Environmental and Domestication Pressures
Source: Animals (Basel). 2026 Oct 29;16(21):3424. doi: 10.3390/ani16213424 (PMC13762271; doi:10.3390/ani16213424)
Supplement: Supplementary file 1 [file animals-16-03424-s001.zip › Table S13.pdf]

Table S13. GO terms significantly enriched for genes overlapping candidate CNVRs under domestication selection.

Term	ID	P_Value	Matched_Genes
caveola	GO:0005901	6.21321E-08	TGFBR2, CDH13, EHD2, KIF18A, TRPC4, TFPI, DLC1
integral component of membrane	GO:0016021	7.27569E-06	FER1L6, CDH10, MALRD1, IL1RAPL1, IL1RL1, UST, SLC25A33, TMEM121, FRRS1, GPRC5C, TNFRSF19, LINGO1, OSBPL5, TMEM192, CDH8, SUSD4, GPR142, VIPR2, PNPLA4, B4GALT2, ATP6V0B, TGFBR2, SLITRK4, HHAT, SLITRK2, TPO, SERTM1, CTSC, INPP5B, KCNF1, ATP2B2, GALNT3, IL13RA2, DEGS2, SEMA5B, SYT2, IMMP2L, PTPRJ, FAT1, CSMD1, VTI1A, CANT1, TMEFF2, TMEM233
interleukin-1 receptor activity	GO:0004908	4.94742E-05	IL1RAPL1, IL18R1, IL1RL1
catenin complex	GO:0016342	8.9664E-05	CDH8, CDH13, CTNNA1, CDH10
cadherin binding	GO:0045296	9.68644E-05	CDH8, CDH13, CTNNA1, TRPC4, CDH10
extracellular space	GO:0005615	0.000247286	TFPI, SERPINA10, ANGPT1, PDGFD, TIMP2, LINGO1, TPO, CTSC, CPA6, CDH13, WNT16, SPIA4, IFNL3A, MMP9, PLTP,

			WNT9B, ENGASE
homophilic cell adhesion via plasma membrane adhesion molecules	GO:0007156	0.000300861	CDH10, CDH13, PCDH9, ROBO1, CDH8, FAT1
cytoplasm	GO:0005737	0.000344495	ASB10, CDH13, AGL, EHD2, IPO13, SMAD6, PRKAG2, FABP1, CIT, TYK2, CRADD, CACNA1G, LIG1, PPP1R12B, PNPLA4, MTMR1, TRAF1, PRKAB1, DST, PPARG, OLA1, SMYD1, METTL21EP, FTO, SASH1, UBE4A, RBFOX1, ADCY2, MTF1, TACC2, C1QTNF1, MYO6, MSRA, ROBO1, WNT16, TUBGCP3
activation of cysteine-type endopeptidase activity involved in apoptotic process	GO:0006919	0.000446509	ROBO1, PPARG, DLC1, CRADD
plasma membrane	GO:0005886	0.000577588	CDH10, CEMIP, IL1RAPL1, EHD2, GRIA3, SLC6A9, SCN9A, GPRC5C, TNFRSF19, LINGO1, NSF, CACNA1G, VIPR2, TTYH2, ANGPT1, MTMR1, TGFBR2, GRID1, SYT2, EPN3, LDB2, GPR142, ROBO1, TMEFF2, MYO6, GRIN2A, PTPRJ
calcium ion binding	GO:0005509	0.000613807	PCDH9, CDH13, CDH10, TPO, SYT2, CDH8, FAT1,

			CANT1, PLA2G4A, GALNT3, DST, EPDR1
ionotropic glutamate receptor signaling pathway	GO:0035235	0.000726338	GRIA3, GRIN2A, GRID1
regulation of presynapse assembly	GO:1905606	0.000835758	IL1RAPL1, SLITRK4, SLITRK2
calcium-dependent cell-cell adhesion via plasma membrane cell adhesion molecules	GO:0016339	0.000835758	CDH8, CDH13, CDH10
ligand-gated ion channel activity	GO:0015276	0.000835758	GRIA3, GRIN2A, GRID1
signaling receptor activity	GO:0038023	0.000949611	GRIA3, TNFRSF19, GRID1, GRIN2A, TLR5
intracellular membrane-bounded organelle	GO:0043231	0.001254931	EHD2, CTSA, SERTM1, EPN3, ATP2B2, CTNNA1, GPRC5C, OSBPL5
LBD domain binding	GO:0050693	0.001478408	PPARG, SPON1
negative regulation of cell adhesion	GO:0007162	0.001538389	TGFBR2, CDH13, ANGPT1
actin binding	GO:0003779	0.001614791	EVL, DST, KIF18A, MYO6, WIPF3, SPTBN1
membrane	GO:0016020	0.001725237	TGFBR2, INPP5B, PDGFD, DST, MYO6, TMEM233, PNPLA4, TYK2, HPSE2, MTMR1, OSBPL5
type I transforming growth factor beta receptor binding	GO:0034713	0.001960147	TGFBR2, SMAD6
blood circulation	GO:0008015	0.001960147	FLI1, IMMP2L
axon guidance receptor activity	GO:0008046	0.001960147	EPHA7, ROBO1
glutamatergic synapse	GO:0098978	0.002005191	SLITRK4, SLITRK2, EPHA7, IL1RAPL1, CDH8, GRIN2A, GRID1
cell-cell adhesion via	GO:0098742	0.002305656	CDH8, CDH13,

plasma-membrane adhesion molecules			CDH10
regulation of ion transmembrane transport	GO:0034765	0.002452211	CACNA1G, KCNH1, KCNF1, SCN9A
nucleus	GO:0005634	0.002491748	CRADD, ASB10, TOX, AGL, IPO13, SMAD6, PRKAG2, PHF19, ZNF335, USP28, TYK2, NCOR2, GBX1, SAMD11, CSRNP3, ETS1, LIG1, DLGAP5, DLC1, PRKAB1, KIF18A, FLI1, ZNF521, ERCC5, LDB2, SMYD1, PCIF1, PLA2G4A, USP36, UBE4A, RBFOX1, MTF1, MYO6, LEO1, FAT1
cell-substrate adhesion	GO:0031589	0.002506053	SMAD6, ANGPT1
nucleotide-activated protein kinase complex	GO:0031588	0.002506053	PRKAG2, PRKAB1
antioxidant activity	GO:0016209	0.002506053	SELENOW, FABP1
extracellular matrix	GO:0031012	0.002807419	TGFBR2, SPON1, TIMP2, LINGO1, MMP9, HPSE2
response to drug	GO:0042493	0.002883715	SLC12A5, TGFBR2, GRIN2A, HTR2C
startle response	GO:0001964	0.003115008	CSMD1, GRIN2A
keratinocyte proliferation	GO:0043616	0.003115008	WNT16, CDH13
protein deglycosylation	GO:0006517	0.003115008	C1QTNF1, ENGASE
neuron projection morphogenesis	GO:0048812	0.003274289	ZNF335, SHTN1, NYAP2
clathrin-coated endocytic vesicle	GO:0045334	0.003785907	CEMIP, MYO6
microtubule depolymerization	GO:0007019	0.004517659	NCKAP5, KIF18A
regulation of peptidyl-tyrosine phosphorylation	GO:0050730	0.004517659	EPHA7, PDGFD

mitotic cytokinesis	GO:0000281	0.004790907	CHMP4B, SPTBN1, CIT
positive regulation of cell migration	GO:0030335	0.005042007	TGFBR2, CDH13, CEMIP, PDGFD, SEMA5B
cerebral cortex neuron differentiation	GO:0021895	0.005309188	TOX, ZNF335
positive regulation of epidermal growth factor receptor signaling pathway	GO:0045742	0.005309188	PDE6G, MMP9
positive regulation of dendrite extension	GO:1903861	0.005309188	SYT2, RIMS2
negative regulation of endopeptidase activity	GO:0010951	0.005355376	SERPINA10, SPIA4, TIMP2, TFPI
perinuclear region of cytoplasm	GO:0048471	0.005821905	RARB, SHTN1, TMEM192, MYO6, EPN3, FAT1, EHD2, GALNT3
ruffle membrane	GO:0032587	0.0058735	DLC1, MYO6, PTPRJ
MyD88-dependent toll-like receptor signaling pathway	GO:0002755	0.00615943	ETS1, TLR5
calcium ion-regulated exocytosis of neurotransmitter	GO:0048791	0.00615943	SYT2, RIMS2
intercellular bridge	GO:0045171	0.006264167	EHD2, PCIF1, MSRA
late endosome membrane	GO:0031902	0.006669991	CHMP4B, VTI1A, MREG
actin filament-based movement	GO:0030048	0.007067336	WIPF3, MYO6
membrane depolarization during action potential	GO:0086010	0.007067336	CACNA1G, SCN9A
positive regulation of peptidyl-threonine phosphorylation	GO:0010800	0.007067336	PRKAG2, CEMIP
positive regulation of JNK cascade	GO:0046330	0.007091114	TRAF1, WNT16, TNFRSF19
calcium ion import	GO:0070509	0.00803187	TRPC4, CACNA1G
ATPase activity	GO:0016887	0.008186709	OLA1, KIF18A, ABCF2, NSF, KIF3A
serine-type	GO:0004867	0.008931196	SERPINA10, SPIA4,

endopeptidase inhibitor activity			TFPI
voltage-gated sodium channel complex	GO:0001518	0.009052011	CACNA1G, SCN9A
voltage-gated sodium channel activity	GO:0005248	0.009052011	CACNA1G, SCN9A
nervous system development	GO:0007399	0.009278391	NAV1, LDB2, NAV2, RBFOX1
PDZ domain binding	GO:0030165	0.009430706	ATP2B2, NSF, CIT
cysteine-type endopeptidase activity	GO:0004197	0.009946224	CTSC, USP36, USP28
regulation of I-kappaB kinase/NF-kappaB signaling	GO:0043122	0.010126748	TRAF1, ANGPT1
plasma membrane organization	GO:0007009	0.010126748	FER1L6, SPTBN1
protein kinase activator activity	GO:0030295	0.010126748	PRKAG2, GPRC5C
positive regulation of reactive oxygen species metabolic process	GO:2000379	0.010126748	TGFBR2, IFNL3A
positive regulation of DNA binding	GO:0043388	0.010126748	PPARG, MMP9
positive regulation of apoptotic signaling pathway	GO:2001235	0.010126748	CTSC, CRADD
postsynaptic density membrane	GO:0098839	0.010126748	ATP2B2, GRIN2A
neuronal action potential	GO:0019228	0.010126748	CACNA1G, SCN9A
cardiac epithelial to mesenchymal transition	GO:0060317	0.010126748	WNT16, TGFBR2
positive regulation of phosphatidylinositol 3-kinase signaling	GO:0014068	0.011025663	WNT16, ANGPT1, PDGFD
cellular oxidant detoxification	GO:0098869	0.011025663	TPO, SELENOW, FABP1
negative regulation of stress fiber assembly	GO:0051497	0.011255085	TMEFF2, DLC1
negative regulation of chondrocyte differentiation	GO:0032331	0.011255085	SMAD6, RARB
regulation of postsynapse	GO:0099175	0.011255085	EPHA7, IL1RAPL1

organization			
phospholipid transport	GO:0015914	0.013668639	PLTP, OSBPL5
regulation of lipid metabolic process	GO:0019216	0.013668639	PPARG, H2AFY
outflow tract septum morphogenesis	GO:0003148	0.013668639	SMAD6, RARB
apoptotic process	GO:0006915	0.013838706	EPHA7, TGFBR2, CTSC, DLC1
microtubule-based movement	GO:0007018	0.014656901	DNAI2, KIF18A, KIF3A
adherens junction organization	GO:0034332	0.014951928	CDH8, CDH10
hyaluronic acid binding	GO:0005540	0.014951928	CEMIP, IMPG1
negative regulation of intrinsic apoptotic signaling pathway	GO:2001243	0.014951928	NOC2L, MMP9
microtubule bundle formation	GO:0001578	0.014951928	NAV1, NCKAP5
locomotory behavior	GO:0007626	0.01599998	NAV2, B4GALT2, HTR2C
spindle assembly	GO:0051225	0.016284958	CENPW, TUBGCP3
synaptic transmission, glutamatergic	GO:0035249	0.016284958	CDH8, GRID1
platelet-derived growth factor receptor signaling pathway	GO:0048008	0.016284958	PDGFD, PTPRJ
microtubule cytoskeleton	GO:0015630	0.017410025	NAV1, EHD2, PCIF1
polyubiquitin modification-dependent protein binding	GO:0031593	0.017666797	AGL, ETS1
positive regulation of cell adhesion	GO:0045785	0.017666797	ANGPT1, PTPRJ
negative regulation of cell migration	GO:0030336	0.018140242	ROBO1, TMEFF2, DLC1
ADP binding	GO:0043531	0.019096525	PRKAG2, MYO6
regulation of cytosolic calcium ion concentration	GO:0051480	0.019096525	ATP2B2, TRPC4
ureteric bud development	GO:0001657	0.019096525	SMAD6, RARB
cell-cell junction assembly	GO:0007043	0.020573231	CDH8, CDH10

positive regulation of inflammatory response	GO:0050729	0.020573231	ETS1, IL1RL1
Golgi to plasma membrane protein transport	GO:0043001	0.020573231	NSF, SPTBN1
intra-Golgi vesicle-mediated transport	GO:0006891	0.020573231	VTI1A, NSF
cell leading edge	GO:0031252	0.020573231	LDB2, SHTN1
nuclear body	GO:0016604	0.02058099	SMAD6, USP28, FLI1, NCOR2, MSRA
focal adhesion	GO:0005925	0.020704758	EVL, DST, DLC1, IL1RL1
lipid binding	GO:0008289	0.022044142	FABP1, DLC1, OSBPL5
regulation of postsynaptic membrane potential	GO:0060078	0.022096018	GRIA3, GRID1
positive regulation of blood vessel endothelial cell migration	GO:0043536	0.022096018	ETS1, ANGPT1
chemorepellent activity	GO:0045499	0.022096018	EPHA7, SEMA5B
mitotic metaphase plate congression	GO:0007080	0.022096018	CHMP4B, KIF18A
sprouting angiogenesis	GO:0002040	0.022096018	CDH13, ANGPT1
cytoplasmic side of plasma membrane	GO:0009898	0.023664001	TRAF1, CHMP4B
response to retinoic acid	GO:0032526	0.025276306	PPARG, RARB
clathrin-coated pit	GO:0005905	0.025276306	MYO6, EPN3
integral component of postsynaptic density membrane	GO:0099061	0.025276306	GRIN2A, GRID1
histone deacetylase complex	GO:0000118	0.025276306	HDAC9, NCOR2
calcium ion transmembrane transport	GO:0070588	0.026370042	ATP2B2, TRPC4, CACNA1G
neurogenesis	GO:0022008	0.026932071	NAV2, GRIN2A
transmitter-gated ion channel activity involved in regulation	GO:1904315	0.026932071	GRIA3, GRID1

of postsynaptic membrane potential			
negative regulation of phosphatase activity	GO:0010923	0.026932071	PCIF1, CSRNP3
positive regulation of endothelial cell migration	GO:0010595	0.026932071	ETS1, SASH1
phosphatidylinositol dephosphorylation	GO:0046856	0.026932071	INPP5B, MTMR1
cation channel activity	GO:0005261	0.026932071	CACNA1G, SCN9A
sodium ion transmembrane transport	GO:0035725	0.027285802	SLC6A9, CACNA1G, SCN9A
neuron projection	GO:0043005	0.027711512	EPHA7, SLC12A5, CDH13, GRIN2A, CACNA1G
negative chemotaxis	GO:0050919	0.028630444	EPHA7, SEMA5B
visual learning	GO:0008542	0.028630444	B4GALT2, GRIN2A
frizzled binding	GO:0005109	0.028630444	WNT16, WNT9B
drug binding	GO:0008144	0.028630444	PPARG, HTR2C
sensory perception of sound	GO:0007605	0.029167757	NAV2, CEMIP, MYO6
cellular response to UV	GO:0034644	0.030370587	NOC2L, USP28
protein kinase binding	GO:0019901	0.031482753	PRKAB1, PRKAG2, H2AFY, NSF, PPP1R12B, CACUL1
autophagy	GO:0006914	0.032151669	CHMP4B, VTI1A
hormone-mediated signaling pathway	GO:0009755	0.032151669	PPARG, RARB
clathrin binding	GO:0030276	0.032151669	SYT2, EPN3
positive regulation of NIK/NF-kappaB signaling	GO:1901224	0.033972874	IL18R1, SASH1
kinesin complex	GO:0005871	0.033972874	KIF18A, KIF3A
positive regulation of MAP kinase activity	GO:0043406	0.033972874	ROBO1, PDGFD
intermembrane lipid transfer	GO:0120009	0.033972874	PLTP, OSBPL5
scaffold protein binding	GO:0097110	0.035833394	CACNA1G, CIT
presynaptic membrane	GO:0042734	0.035833394	GRIN2A, RIMS2
cytoskeleton	GO:0005856	0.035882168	TYK2, EVL, TMOD3, CIT

chromatin organization	GO:0006325	0.039669207	PHF19, H2AFY
nuclear receptor activity	GO:0004879	0.04164294	PPARG, RARB
positive regulation of stress fiber assembly	GO:0051496	0.04164294	TGFBR2, EVL
cytokine activity	GO:0005125	0.043020695	WNT16, IFNL3A, WNT9B
receptor complex	GO:0043235	0.043189755	EPHA7, IL13RA2, GPRC5C, TGFBR2
positive regulation of synapse assembly	GO:0051965	0.043652866	SLITRK4, SLITRK2
flagellated sperm motility	GO:0030317	0.043652866	INPP5B, PLTP
filopodium	GO:0030175	0.043652866	FAT1, MYO6
brain development	GO:0007420	0.04420078	EPHA7, ZNF335, IMMP2L
membrane raft	GO:0045121	0.045396911	TGFBR2, ANGPT1, DLC1
actin filament organization	GO:0007015	0.045396911	FAT1, MYO6, TMOD3
endocytic vesicle	GO:0030139	0.045698233	MYO6, EHD2
phosphatidylserine binding	GO:0001786	0.045698233	SYT2, OSBPL5
metal ion binding	GO:0046872	0.047276066	TGFBR2, SPON1, TIMP2, ADCY2, SMAD6, PHF19, NSF, CHN2, OLA1, CIT, RIMS2
cytoplasmic microtubule organization	GO:0031122	0.047778294	DST, TUBGCP3
memory	GO:0007613	0.047778294	B4GALT2, GRIN2A
chromatin binding	GO:0003682	0.047907708	SAMD11, SMAD6, FLI1, PHF19, FABP1, NCOR2
microvillus	GO:0005902	0.049892317	ANGPT1, MYO6
microtubule motor activity	GO:0003777	0.049892317	KIF18A, KIF3A
cell fate commitment	GO:0045165	0.049892317	WNT16, WNT9B
