# Supplementary material for: Genome-Wide Analysis Reveals Copy Number Variation-Mediated Adaptive Evolution in Chickens Under Diverse Environmental and Domestication Pressures
Source: Animals (Basel). 2026 Oct 29;16(21):3424. doi: 10.3390/ani16213424 (PMC13762271; doi:10.3390/ani16213424)
Supplement: Supplementary file 1 [file animals-16-03424-s001.zip › Table S12.pdf]

Table S12. KEGG pathways significantly enriched for genes overlapping candidate CNVRs under domestication selection.

Term	ID	P_Value	Matched_Genes
Calcium signaling pathway	gga04020	0.005342369	TRDN, ADCY2, CACNA1G, GRIN2A, HTR2C, ATP2B2
Melanogenesis	gga04916	0.008950232	WNT3, WNT16, WNT9B, ADCY2
Cytokine-cytokine receptor interaction	gga04060	0.009047001	IL13RA2, IL1RL1, TGFBR2, IL18R1, IFNL3A, TNFRSF19
Glycosaminoglycan biosynthesis - chondroitin sulfate / dermatan sulfate	gga00532	0.013668639	CHPF2, UST
Endocytosis	gga04144	0.015449531	TGFBR2, EHD2, CHMP4B, EPN3, WIPF3, CYTH1
PPAR signaling pathway	gga03320	0.019651192	PPARG, PLTP, FABP1
Adipocytokine signaling pathway	gga04920	0.019651192	PRKAG2, IRS2, PRKAB1
FoxO signaling pathway	gga04068	0.023583215	TGFBR2, PRKAG2, IRS2, PRKAB1
Adherens junction	gga04520	0.024589062	TGFBR2, PTPRJ, CTNNA1
Metabolic pathways	gga01100	0.031375716	ATP6V0B, DEGS2, AGL, CANT1, ADCY2, ACSS3, CHPF2, INPP5B, HPSE2, TPO, ENO4, PDE6G, ACOT8, GADL1, PLA2G4A, B4GALT2, MTMR1, GALNT3
Gap junction	gga04540	0.038462162	PDGFD, HTR2C, ADCY2
Nucleotide excision repair	gga03420	0.045698233	ERCC5, LIG1
