# Supplementary material for: Genome-Wide Analysis Reveals Copy Number Variation-Mediated Adaptive Evolution in Chickens Under Diverse Environmental and Domestication Pressures
Source: Animals (Basel). 2026 Oct 29;16(21):3424. doi: 10.3390/ani16213424 (PMC13762271; doi:10.3390/ani16213424)
Supplement: Supplementary file 1 [file animals-16-03424-s001.zip › Table S11.pdf]

Table S11. Candidate CNVRs and annotated genes under positive selection in the domestication group.

CHROM	BIN_START	BIN_END	Gene_Name
1	23400001	23440000	FAM3C
1	23400001	23440000	WNT16
1	23400001	23440000	CPED1
1	27860001	27900000	IMMP2L
1	27880001	27920000	IMMP2L
1	40220001	40260000	ACSS3
1	40240001	40280000	ACSS3
1	45000001	45040000	CRADD
1	59920001	59960000	gene:ENSGALG00010012109
1	96260001	96300000	ROBO1
1	96280001	96320000	ROBO1
1	105720001	105760000	MRPS6
1	105720001	105760000	SLC5A3
1	105740001	105780000	MRPS6
1	105740001	105780000	SLC5A3
1	116440001	116480000	IL1RAPL1
1	119960001	120000000	MAP7D2
1	126280001	126320000	PNPLA4
1	131200001	131240000	GABRG3
1	131220001	131260000	GABRG3
1	133960001	134000000	IL1RL1
1	133980001	134020000	IL1RL1
1	133980001	134020000	IL18R1
1	137980001	138020000	TUBGCP3
1	138660001	138700000	IRS2
1	142700001	142740000	gene:ENSGALG00010006086
1	142720001	142760000	ERCC5
1	142720001	142760000	METTL21EP
1	145740001	145780000	HS6ST3
1	145760001	145800000	HS6ST3
1	159180001	159220000	PCDH9
1	159200001	159240000	PCDH9
1	171720001	171760000	TRPC4
1	171740001	171780000	TRPC4
1	172340001	172380000	SERTM1
1	177360001	177400000	TNFRSF19
1	177380001	177420000	TNFRSF19
1	181780001	181820000	PDGFD
1	181800001	181840000	PDGFD
1	188120001	188160000	CTSC

2	140001	180000	CHPF2
2	140001	180000	ABCF2
2	140001	180000	H2BK1
2	140001	180000	ASB10
2	160001	200000	H2BK1
2	160001	200000	ASB10
2	160001	200000	GBX1
2	6420001	6460000	PRKAG2
2	6440001	6480000	PRKAG2
2	9620001	9660000	VIPR2
2	18780001	18820000	MALRD1
2	18800001	18840000	MALRD1
2	29000001	29040000	HDAC9
2	29020001	29060000	HDAC9
2	29200001	29240000	HDAC9
2	33680001	33720000	CHN2
2	33680001	33720000	gene:ENSGALG00010002631
2	33700001	33740000	WIPF3
2	37520001	37560000	RARB
2	39760001	39800000	TGFBR2
2	39760001	39800000	GADL1
2	39780001	39820000	GADL1
2	43860001	43900000	EIF1B
2	43860001	43900000	gene:ENSGALG00010015863
2	43880001	43920000	EIF1B
2	43880001	43920000	gene:ENSGALG00010015863
2	43880001	43920000	MYRIP
2	46020001	46060000	EPDR1
2	71860001	71900000	CDH10
2	78900001	78940000	ADCY2
2	78920001	78960000	ADCY2
2	93880001	93920000	gene:ENSGALG00010001227
2	93900001	93940000	gene:ENSGALG00010001227
2	103420001	103460000	ZNF521
2	103440001	103480000	ZNF521
2	110240001	110280000	gene:ENSGALG00010004565
2	111920001	111960000	TOX
2	115180001	115220000	CPA6
2	115200001	115240000	CPA6
2	129440001	129480000	RIMS2
2	130900001	130940000	ANGPT1
2	130920001	130960000	ANGPT1
2	137240001	137280000	FER1L6

2	143900001	143940000	KCNK9
3	2900001	2940000	SPTBN1
3	2920001	2960000	SPTBN1
3	17460001	17500000	SUSD4
3	17460001	17500000	TLR5
3	17480001	17520000	SUSD4
3	17480001	17520000	TLR5
3	19340001	19380000	SPATA17
3	22140001	22180000	KCNH1
3	22140001	22180000	HHAT
3	22160001	22200000	KCNH1
3	22160001	22200000	HHAT
3	46920001	46960000	SASH1
3	47540001	47580000	UST
3	47560001	47600000	UST
3	59220001	59260000	CENPW
3	60640001	60680000	TRDN
3	73640001	73680000	EPHA7
3	73660001	73700000	EPHA7
3	80100001	80140000	IMPG1
3	80120001	80160000	IMPG1
3	80120001	80160000	MYO6
3	84820001	84860000	gene:ENSGALG00010001483
3	86640001	86680000	DST
3	86660001	86700000	DST
3	89300001	89340000	CSMD1
3	89320001	89360000	CSMD1
3	92460001	92500000	TPO
3	96720001	96760000	KCNF1
3	106240001	106280000	MSRA
4	2740001	2780000	HTR2C
4	2760001	2800000	HTR2C
4	2760001	2800000	IL13RA2
4	9780001	9820000	SLITRK4
4	9800001	9840000	SLITRK4
4	15840001	15880000	GRIA3
4	17640001	17680000	CD99L2
4	17640001	17680000	MTMR1
4	18840001	18880000	SLITRK2
4	18860001	18900000	SLITRK2
4	23180001	23220000	APELA
4	23200001	23240000	APELA
4	23200001	23240000	TMEM192

4	37380001	37420000	RPL34
4	61260001	61300000	FAT1
4	61280001	61320000	FAT1
4	64080001	64120000	DLC1
4	75880001	75920000	LDB2
4	75900001	75940000	LDB2
4	85560001	85600000	gene:ENSGALG00010017386
4	85560001	85600000	gene:ENSGALG00010017378
4	85560001	85600000	gene:ENSGALG00010017389
4	85560001	85600000	SMYD1
4	85580001	85620000	SMYD1
4	85580001	85620000	FABP1
5	1960001	2000000	NAV2
5	1980001	2020000	NAV2
5	4100001	4140000	KIF18A
5	4100001	4140000	METTL15P1
5	4120001	4160000	METTL15P1
5	6140001	6180000	SPON1
5	6160001	6200000	SPON1
5	6180001	6220000	SPON1
5	6200001	6240000	SPON1
5	12440001	12480000	PTPRJ
5	12460001	12500000	PTPRJ
5	12460001	12500000	OSBPL5
5	45020001	45060000	SERPINA10
5	45020001	45060000	SPIA4
5	47980001	48020000	EVL
5	47980001	48020000	DEGS2
5	47980001	48020000	YY2
5	55820001	55860000	FBXO34
5	55820001	55860000	DLGAP5
6	2760001	2800000	GRID1
6	2780001	2820000	GRID1
6	5360001	5400000	RASGEF1A
6	23000001	23040000	HPSE2
6	23000001	23040000	HPS1
6	23020001	23060000	HPSE2
6	23020001	23060000	HPS1
6	23020001	23060000	PYROXD2
6	23020001	23060000	R3HCC1L
6	27660001	27700000	VTI1A
6	27680001	27720000	VTI1A
6	29740001	29780000	ENO4

6	29740001	29780000	SHTN1
6	29760001	29800000	ENO4
6	29760001	29800000	SHTN1
6	30620001	30660000	CACUL1
6	31920001	31960000	TACC2
6	31940001	31980000	TACC2
7	1100001	1140000	TFPI
7	4600001	4640000	MREG
7	4600001	4640000	gene:ENSGALG00010012015
7	4600001	4640000	IFNL3A
7	4600001	4640000	gene:ENSGALG00010012045
7	7860001	7900000	TMEFF2
7	16640001	16680000	OLA1
7	19160001	19200000	SCN9A
7	19220001	19260000	gene:ENSGALG00010026770
7	19320001	19360000	GALNT3
7	19320001	19360000	CSRNP3
7	19340001	19380000	CSRNP3
7	26160001	26200000	SEMA5B
7	29620001	29660000	NCKAP5
7	29640001	29680000	NCKAP5
8	3880001	3920000	TMEM121
8	10000001	10040000	PLA2G4A
8	10020001	10060000	PLA2G4A
8	11060001	11100000	COL11A1
8	11060001	11100000	gene:ENSGALG00010023368
8	11780001	11820000	gene:ENSGALG00010024001
8	11780001	11820000	AGL
8	11800001	11840000	gene:ENSGALG00010024001
8	11800001	11840000	AGL
8	11800001	11840000	FRRS1
8	20040001	20080000	IPO13
8	20040001	20080000	DPH2
8	20040001	20080000	ATP6V0B
8	20040001	20080000	B4GALT2
8	20040001	20080000	SLC6A9
9	8400001	8440000	NYAP2
9	8420001	8460000	NYAP2
9	20040001	20080000	gene:ENSGALG00010016955
9	20060001	20100000	gene:ENSGALG00010016955
10	2760001	2800000	LINGO1
10	2780001	2820000	LINGO1
10	9000001	9040000	LEO1

10	9000001	9040000	TMOD3
10	9020001	9060000	LEO1
10	9020001	9060000	TMOD3
10	12260001	12300000	gene:ENSGALG00010019292
10	12260001	12300000	CEMIP
10	12280001	12320000	CEMIP
10	12280001	12320000	gene:ENSGALG00010019498
10	18720001	18760000	SMAD6
11	4260001	4300000	FTO
11	4280001	4320000	FTO
11	12540001	12580000	CDH8
11	12560001	12600000	CDH8
11	15940001	15980000	CDH13
12	4040001	4080000	ATP2B2
12	4060001	4100000	ATP2B2
12	5020001	5060000	PPARG
12	5040001	5080000	PPARG
12	14840001	14880000	gene:ENSGALG00010019696
12	16840001	16880000	PDZRN3
12	16860001	16900000	PDZRN3
13	2200001	2240000	SIL1
13	2200001	2240000	CTNNA1
13	2220001	2260000	SIL1
13	2220001	2260000	CTNNA1
13	4920001	4960000	TENM2
13	8040001	8080000	ARHGEF37
13	8040001	8080000	CSNK1A1
13	8060001	8100000	CSNK1A1
13	14980001	15020000	H2AFY
13	16580001	16620000	KIF3A
13	16580001	16620000	CCNI2
13	16580001	16620000	SEPTIN8
14	1800001	1840000	FOXL3
14	9340001	9380000	GRIN2A
14	11480001	11520000	RBFOX1
14	11500001	11540000	RBFOX1
15	4660001	4700000	NCOR2
15	6940001	6980000	SGSM1
15	6940001	6980000	KIAA1671
15	9620001	9660000	CIT
15	9620001	9660000	PRKAB1
15	9620001	9660000	TMEM233
15	9640001	9680000	CIT

15	9640001	9680000	PRKAB1
15	9640001	9680000	TMEM233
15	9640001	9680000	CCDC60
17	8880001	8920000	PSMD5
17	8880001	8920000	CUTA
17	8880001	8920000	PHF19
17	8880001	8920000	TRAF1
18	9740001	9780000	TTYH2
18	9740001	9780000	DNAI2
18	9740001	9780000	KIF19
18	9740001	9780000	BTBD17
18	9740001	9780000	GPR142
18	9740001	9780000	GPRC5C
18	9740001	9780000	OXLD1
18	9740001	9780000	gene:ENSGALG00010029508
18	9740001	9780000	PDE6G
18	9740001	9780000	TSPAN10
18	10380001	10420000	ENGASE
18	10380001	10420000	CANT1
18	10380001	10420000	C1QTNF1
18	10380001	10420000	TIMP2
18	10380001	10420000	USP36
18	10380001	10420000	CYTH1
18	10900001	10940000	CACNA1G
18	10900001	10940000	SPATA20
18	10920001	10960000	CACNA1G
18	10920001	10960000	SPATA20
18	10920001	10960000	EPN3
18	10920001	10960000	MYCBPAP
20	2400001	2440000	gene:ENSGALG00010024375
20	2420001	2460000	CHMP4B
20	10860001	10900000	ACOT8
20	10860001	10900000	CTSA
20	10860001	10900000	ZSWIM1
20	10860001	10900000	NEURL2
20	10860001	10900000	PLTP
20	10860001	10900000	PCIF1
20	10860001	10900000	gene:ENSGALG00010016039
20	10860001	10900000	ZNF335
20	10880001	10920000	PLTP
20	10880001	10920000	PCIF1
20	10880001	10920000	gene:ENSGALG00010016039
20	10880001	10920000	ZNF335

20	10880001	10920000	MMP9
20	10880001	10920000	SLC12A5
21	2880001	2920000	NOC2L
21	2880001	2920000	SAMD11
21	2900001	2940000	NOC2L
21	2900001	2940000	SAMD11
21	3380001	3420000	SLC25A33
21	3380001	3420000	TMEM201
21	3400001	3440000	SLC25A33
21	3400001	3440000	TMEM201
23	3880001	3920000	SF3A3
23	3880001	3920000	INPP5B
23	3880001	3920000	MTF1
24	980001	1020000	ETS1
24	1000001	1040000	FLI1
24	4460001	4500000	UBE4A
24	4460001	4500000	gene:ENSGALG00010024305
24	4460001	4500000	gene:ENSGALG00010026127
24	4460001	4500000	USP28
25	520001	560000	gene:ENSGALG00010027354
26	700001	740000	NAV1
26	700001	740000	SYT2
26	700001	740000	PPP1R12B
26	720001	760000	PPP1R12B
27	740001	780000	WNT9B
27	740001	780000	WNT3
27	740001	780000	NSF
29	660001	700000	gene:ENSGALG00010000686
29	660001	700000	gene:ENSGALG00010000709
29	660001	700000	gene:ENSGALG00010000713
29	660001	700000	gene:ENSGALG00010000716
29	660001	700000	gene:ENSGALG00010000596
29	680001	720000	gene:ENSGALG00010000713
30	1	40000	FDX2
30	1	40000	RAVER1
30	1	40000	TYK2
30	1	40000	gene:ENSGALG00010002450
31	40001	80000	gene:ENSGALG00010004397
31	40001	80000	gene:ENSGALG00010004406
31	40001	80000	gene:ENSGALG00010004420
31	40001	80000	LIG1
31	60001	100000	gene:ENSGALG00010004397
31	60001	100000	gene:ENSGALG00010004406

31	60001	100000	gene:ENSGALG00010004420
31	60001	100000	LIG1
31	60001	100000	gene:ENSGALG00010004622
31	60001	100000	gene:ENSGALG00010004630
31	60001	100000	gene:ENSGALG00010004705
35	1	40000	METTL3
35	1	40000	gene:ENSGALG00010003473
36	1	40000	gene:ENSGALG00010000623
36	1	40000	gene:ENSGALG00010000627
36	1	40000	gene:ENSGALG00010000630
36	1	40000	gene:ENSGALG00010000631
36	1	40000	gene:ENSGALG00010000634
38	220001	260000	BICRA
38	220001	260000	EHD2
38	220001	260000	NOP53
38	220001	260000	gene:ENSGALG00010008205
38	220001	260000	SELENOW
38	220001	260000	H3-IX
38	220001	260000	OTX5
39	1	40000	gene:ENSGALG00010000483
39	1	40000	gene:ENSGALG00010000486
39	1	40000	gene:ENSGALG00010000491
