# Supplementary material for: Genome-Wide Analysis Reveals Copy Number Variation-Mediated Adaptive Evolution in Chickens Under Diverse Environmental and Domestication Pressures
Source: Animals (Basel). 2026 Oct 29;16(21):3424. doi: 10.3390/ani16213424 (PMC13762271; doi:10.3390/ani16213424)
Supplement: Supplementary file 1 [file animals-16-03424-s001.zip › Table S10.pdf]

**Table S10. GO terms significantly enriched for genes overlapping candidate CNVRs under high-altitude adaptation selection.**

| Term | ID | P_Value | Matched_Genes |
|---|---|---|---|
| plasma membrane | GO:0005886 | 2.40737E-11 | PTGFR, UBL3, GRIA2, GRIA3, ANO6, CRYAB, SMAD3, STXBP1, ATP23, TENM1, PRKAR2B, STXBP5, WHRN, TTC7A, ATP6V1G3, VSTM5, FZD10, ADCYAP1R1, VANGL2, GPRC5B, ITPR1, LY6CLEL, GRID2, PIK3CG, RNF114, MAGI3, ANO3, GHRHR-LR, ANO5, SCN4A, USH1C, TNFRSF19, PENK, SDCBP, MAGI1, IFITM5, ARVCF, ARL14EP, JPT2, |

| | | | |
|---|---|---|---|
| | | | CRTAM, TMPRSS7, LDB2, SUCLG1, DLL4, PHLDB2, GABRB3, CALD1, GH, ILDR1, APBA2, PTPN13, DNAJC5, PTPRE, KCNT2, ABCC8, PIK3C2G |
| nucleus | GO:0005634 | 1.66194E-07 | CLIC6, NFATC2, TBX20, USP15, CRYAB, SIRT1, TOX, SMAD3, BRF1, TENM1, NHLH1, PLCB4, KAT7, SKI, NCOR2, DHX37, PHTF2, FIGNL1, RGS20, HSPH1, UBAP2L, CDC73, MECOM, BACH2, CGGBP1, USP46, NPAS2, DDX17, SOBP, |

| | | | |
|---|---|---|---|
| | | | UBXN2B, MED17, BRDT, MAPK8, TNKS2, TOP2A, SIRT3, MGMT, CRAMP1, RRAGA, ARVCF, IQGAP3, LPP, LDB2, APPL2, ZNF654, KHDRBS2, CBY1, GH, CSRNP1, TAGLN3, RBFOX1, PRDM11, DMRTA2, LMO1, PSMD13, CASP9, PTPN13, PTPRE, RUNX1, ZBTB46, CCND1, WDR48, FBXL21, UBE2W |
| chloride channel activity | GO:0005254 | 2.3717E-06 | CLIC6, ANO5, ANO6, GABRQ, ANO3, GABRA5, GABRB3 |
| cytosol | GO:0005829 | 3.52288E-05 | NFATC2, ANO6, SEC24B, CRYAB, |

| | | | |
|---|---|---|---|
| | | | SMAD3, STXBP1, ATP23, STXBP5, CASK, GABRA5, CASP9, GPRC5B, HSPH1, CDC73, CDKN3, EIF3A, USP46, ABHD10, NPAS2, UBQLN4, PIK3CG, ARL14EP, RNF114, UBXN2B, USH1C, SNX9, XPNPEP1, SIRT1, FAF1, JPT2, MMS22L, PGGHG, SUCLG1, USP15, BACH2, PHLDB2, SDCBP, URI1, RDM1, HMBOX1, PTPN13, MTHFD1L, RPAP3, PSMD13, KAT7, PRKCZ, ATP6V1G3, DIXDC1, FBXL21, |

| | | | |
|---|---|---|---|
| | | | CHMP4B |
| ion transmembrane transport | GO:0034220 | 3.84183E-05 | GRID2, GRIA2, GRIA3, GABRQ, CHRNA1, GABRA5, GABRB3 |
| metal ion binding | GO:0046872 | 4.06638E-05 | AGMAT, PHF21A, ATP23, CHN1, LIMS1, CACNA1D, NPAS2, PDE7A, RNF114, MIPEP, PHOSPHO1, RNF220, SOBP, PDE5A, CRYAB, LPP, PRKCZ, TRMT44, NT5DC4, BRF1, GH, LMO1, VAV3, LECT2 |
| chloride transmembrane transport | GO:1902476 | 8.99214E-05 | CLIC6, ANO5, ANO6, GABRQ, ANO3, GABRA5, GABRB3 |
| postsynaptic membrane | GO:0045211 | 0.00010502 | KCTD16, GRID2, GRIA2, GRIA3, GABRQ, CHRNA1, |

| | | | |
|---|---|---|---|
| | | | GABRB3 |
| chloride channel complex | GO:0034707 | 0.000115404 | CLIC6, ANO6, GABRB3, GABRQ |
| chemical synaptic transmission | GO:0007268 | 0.00013284 | APBA2, GABRQ, SDCBP, CHRNA1, HTR2C, GABRA5, GABRB3, PENK |
| pericardium morphogenesis | GO:0003344 | 0.000172756 | BMP7, TBX20, DLL4 |
| locomotory behavior | GO:0007626 | 0.000200113 | APBA2, ZDHHC8, GMFB, SOBP, HTR2C, PENK |
| signal transduction | GO:0007165 | 0.000258872 | SIGIRR, ARHGAP22, IFNG, GABRQ, GMFB, PDE7A, IL7, TENM1, MAGI3, CHRNA1, MAGI1, GABRA5, GABRB3, PDE5A |
| negative regulation of transcription by RNA polymerase II | GO:0000122 | 0.000266525 | NFATC2, CDC73, CCND1, TBX20, HMBOX1, PHF21A, SIRT1, SMAD3, |

| | | | |
|---|---|---|---|
| | | | LDB2, DLL4, PPARA, IKZF5, BACH2, URI1, TAGLN3, SKI, NCOR2, LMO1 |
| behavioral fear response | GO:0001662 | 0.000286258 | GABRA5, USP46, PENK, HTR2C |
| natural killer cell differentiation | GO:0001779 | 0.000332842 | TOX, PTPRC, KAT7 |
| dendritic spine | GO:0043197 | 0.000437673 | GRID2, GRIA2, GRIA3, APBA2, PRKAR2B, KCNA4 |
| release of sequestered calcium ion into cytosol | GO:0051209 | 0.000449321 | ITPR1, PTPRC, HTR2C, RASA3 |
| cell-cell junction | GO:0005911 | 0.000506098 | ARVCF, CASK, LIMS1, MAGI3, MAGI1, VANGL2, IQGAP3 |
| cytoplasm | GO:0005737 | 0.000627656 | CLIC6, PTGFR, TBX20, SH2D1A, SMAD3, TENM1, LIMS1, STXBP5, WHRN, FZD10, |

| | | | |
|---|---|---|---|
| | | | IQGAP3, DDX17, RGS20, CDC16, UBAP2L, LGALSL, CDKN3, NPAS2, HDDC2, PIK3CG, MAGI3, MAGI1, MAPK8, TNKS2, TOP2A, SIRT3, SIRT1, ARVCF, LPP, UBASH3B, PIK3C2G, TPD52L1, DDC, SDCBP, SASH1, RDM1, RBFOX1, APBA2, PTPN13, SCARB1, MYLK, CASP9, TRIM9, CCND1, PTPRE, PRKAR2B, SPATA2, TRIM2, VAV3 |
| sensory perception of sound | GO:0007605 | 0.000661037 | CACNA1D, SOBP, WHRN, GABRA5, USH1C, |

| | | | |
|---|---|---|---|
| | | | GABRB3 |
| B cell receptor signaling pathway | GO:0050853 | 0.000669644 | CD79B, NFATC2, PTPRC, VAV3 |
| histone deacetylase complex | GO:0000118 | 0.000669644 | MECOM, TBL1X, PHF21A, NCOR2 |
| parallel fiber to Purkinje cell synapse | GO:0098688 | 0.000713305 | GRIA3, PLCB4, GRID2 |
| integral component of membrane | GO:0016021 | 0.000747363 | PTGFR, TMEM135, ANO5, ANO3, LMF1, SEC63, VSTM5, ADAM33, EDNRB, CCR7, CD200, VANGL2, ADCYAP1R1, DAD1, FZD10, GPRC5B, CNIH1, PHTF2, PHTF1, CD79B, PKDREJ, SLC9A8, DNAJC16, DGKH, GHRHR-LR, ABCC8, KCNA4, TNFRSF19, TMEM53, CUZD1, ILDR1, CTSC, |

| | | | |
|---|---|---|---|
| | | | GPR146, COMT, TMX1, FRMD6, ITPR1, GALNT1, IL13RA2, CRTAM, CYP7A1, SEMA5B, TMEM60, TP53I11, ZDHHC8, PTPRE, KCNT2, PTPRC, TM4SF1a, SIGIRR, DLL4, YIPF5, TMEM18 |
| transmitter-gated ion channel activity involved in regulation of postsynaptic membrane potential | GO:1904315 | 0.000757531 | GABRA5, GRIA3, GABRB3, GRID2 |
| regulation of cell growth | GO:0001558 | 0.000757531 | NANOS1, URI1, IGFBP4, CDC73 |
| positive regulation of B cell proliferation | GO:0030890 | 0.000757531 | IL7, NFATC2, PTPRC, VAV3 |
| intracellular calcium activated chloride channel activity | GO:0005229 | 0.000883075 | ANO5, ANO6, ANO3 |
| ubiquitin binding | GO:0043130 | 0.000940098 | FAF1, WDR48, TOP2A, UBXN2B, SMAD3 |
| ankyrin binding | GO:0030506 | 0.001076437 | CACNA1D, PTPRC, SPTBN1 |

| | | | |
|---|---|---|---|
| focal adhesion | GO:0005925 | 0.001269811 | ARL14EP, LPP, LIMS1, PTPRC, PHLDB2, IQGAP3, TNS4 |
| cochlea development | GO:0090102 | 0.00153861 | GABRA5, GABRB3, SOBP |
| neurotransmitter receptor activity | GO:0030594 | 0.001574025 | GABRA5, GABRB3, GABRQ, CHRNA1, HTR2C |
| GABA-A receptor complex | GO:1902711 | 0.002108587 | GABRA5, GABRB3, GABRQ |
| positive regulation of JUN kinase activity | GO:0043507 | 0.002108587 | FZD10, VANGL2, SASH1 |
| GABA-A receptor activity | GO:0004890 | 0.002108587 | GABRA5, GABRB3, GABRQ |
| nervous system process | GO:0050877 | 0.002324191 | GABRA5, GABRB3, GABRQ, CHRNA1 |
| heterophilic cell-cell adhesion via plasma membrane cell adhesion molecules | GO:0007157 | 0.002436501 | TENM1, CRTAM, GRID2 |
| ionotropic glutamate receptor signaling pathway | GO:0035235 | 0.002436501 | GRIA2, GRIA3, GRID2 |
| regulation of actin cytoskeleton organization | GO:0032956 | 0.002749613 | FZD10, IQGAP3, VANGL2, DIXDC1 |
| ligand-gated ion channel activity | GO:0015276 | 0.002794259 | GRIA2, GRIA3, GRID2 |
| perinuclear region of cytoplasm | GO:0048471 | 0.003163352 | CDKN3, SYNJ2, FIGNL1, |

| | | | |
|---|---|---|---|
| | | | FAF1, TENM1, TPD52L1, LAMB1, NANOS1, TNKS2, GALNT1, FBXW7 |
| nuclear inner membrane | GO:0005637 | 0.003182719 | SIRT1, ITPR1, SMAD3 |
| cholesterol catabolic process | GO:0006707 | 0.003391943 | SCARB1, CYP7A1 |
| peptidyl-lysine deacetylation | GO:0034983 | 0.003391943 | SIRT3, SIRT1 |
| bone mineralization involved in bone maturation | GO:0035630 | 0.003391943 | ANO6, PHOSPHO1 |
| vasoactive intestinal polypeptide receptor activity | GO:0004999 | 0.003391943 | ADCYAP1R1, GHRHR-LR |
| phospholipid scramblase activity | GO:0017128 | 0.003391943 | ANO6, ANO3 |
| proteasome-mediated ubiquitin-dependent protein catabolic process | GO:0043161 | 0.003806249 | SIRT1, TBL1X, TRIM9, UBXN2B, TRIM2, UBE2W |
| positive regulation of transcription by RNA polymerase II | GO:0045944 | 0.004222181 | BMP7, CSRNP1, NFATC2, TBL1X, SIRT1, AKNA, TBX20, MED17, NPAS2, CASK, SMAD3, LDB2, LMO1, GALR1, RUNX1, PPARA, |

| | | | |
|---|---|---|---|
| | | | IKZF5, ZNF654, KAT7, TOP2A |
| synapse | GO:0045202 | 0.004478382 | SDCBP, APBA2, GABRQ, RPL31, MAGI3, CHRNA1, HTR2C, GABRA5, GABRB3 |
| sensory perception of light stimulus | GO:0050953 | 0.004483827 | USH1C, WHRN |
| response to food | GO:0032094 | 0.004483827 | GH, TMEM135 |
| posttranslational protein targeting to endoplasmic reticulum membrane | GO:0006620 | 0.004483827 | CHMP4B, SEC63 |
| posttranslational protein targeting to membrane, translocation | GO:0031204 | 0.004483827 | SEC61G, SEC63 |
| inner ear receptor cell development | GO:0060119 | 0.004483827 | GABRA5, GABRB3 |
| identical protein binding | GO:0042802 | 0.004675776 | DHX8, SIRT1, UBASH3B, SUMF1, ILDR1, CGGBP1, APBA2, UBQLN4, PIK3CG, DLAT, RPIA, WHRN, HTR2C, USP15, HMBOX1, GABRB3, CD79B, FBXW7 |
| neuron projection | GO:0043005 | 0.004955248 | GABRB3, EPHA7, GABRQ, |

| | | | |
|---|---|---|---|
| | | | TENM1, CHRNA1, GABRA5, ADCYAP1R1, IQGAP3 |
| positive regulation of phagocytosis, engulfment | GO:0060100 | 0.005715562 | APPL2, ANO6 |
| 1-phosphatidylinositol-4-phosphate 3-kinase activity | GO:0035005 | 0.005715562 | PIK3C2G, PIK3CG |
| nuclear envelope reassembly | GO:0031468 | 0.005715562 | CHMP4B, UBXN2B |
| negative regulation of wound healing | GO:0061045 | 0.005715562 | CASK, SMAD3 |
| positive regulation of peptide hormone secretion | GO:0090277 | 0.005715562 | LGALSL, ILDR1 |
| molecular adaptor activity | GO:0060090 | 0.006063635 | MAGI3, MAGI1, LAMTOR2, SASH1 |
| transforming growth factor beta receptor signaling pathway | GO:0007179 | 0.006063635 | APPL2, USP15, SIRT1, SMAD3 |
| protein domain specific binding | GO:0019904 | 0.006181027 | FAF1, TRIM9, DIXDC1, IKZF5, SKI, IQGAP3 |
| integral component of plasma membrane | GO:0005887 | 0.006406304 | TM4SF1a, GABRB3, SLC20A1, IFITM5, OPN4-1, EPHA7, ANO6, GABRQ, TENM1, NCSTN, GALR1, CHRNA1, HTR2C, GABRA5, HMCN1, BOC, |

| | | | |
|---|---|---|---|
| | | | SCARB1 |
| peptidyl-tyrosine dephosphorylation | GO:0035335 | 0.006442554 | CDKN3, PTPN13, PTPRE, UBASH3B, PTPRC |
| protein tyrosine phosphatase activity | GO:0004725 | 0.006746273 | CDKN3, PTPN13, PTPRE, UBASH3B, PTPRC |
| positive regulation of gene expression | GO:0010628 | 0.006782827 | PTGFR, TBX20, IL7, DLL4, WHRN, MAPK8, GH |
| nuclear matrix | GO:0016363 | 0.006864312 | TENM1, PRKCZ, NCOR2, CASK |
| transforming growth factor beta receptor binding | GO:0005160 | 0.007083347 | USP15, SMAD3 |
| regulation of response to DNA damage stimulus | GO:2001020 | 0.007083347 | NPAS2, CASP9 |
| myosin II binding | GO:0045159 | 0.007083347 | CALD1, STXBP5 |
| transcription regulator complex | GO:0005667 | 0.007442296 | NFATC2, TBL1X, NPAS2, SMAD3, LDB2, MED17, SKI |
| nucleolus | GO:0005730 | 0.007476062 | SIRT1, ITPR1, RDM1, ARL14EP, EIF3A, CASK, LDB2, MAGI1, GPRC5B, DHX37, |

| | | | |
|---|---|---|---|
| | | | TOP2A, SPTBN1, FBXW7 |
| bicellular tight junction | GO:0005923 | 0.00773194 | CCND1, PRKCZ, MAGI3, MAGI1 |
| dephosphorylation | GO:0016311 | 0.00773194 | NT5DC4, PUDP, PHOSPHO1, HDDC2 |
| SH3 domain binding | GO:0017124 | 0.00773194 | WIPF1, CASP9, KHDRBS2, SYNJ2 |
| drug binding | GO:0008144 | 0.008178787 | TOP2A, HTR2C, PPARA |
| positive regulation of calcium ion transport | GO:0051928 | 0.00858346 | CACNA1D, MYLK |
| phosphatidylinositol kinase activity | GO:0052742 | 0.00858346 | PIK3C2G, PIK3CG |
| laminin complex | GO:0043256 | 0.00858346 | LAMB1, LAMB4 |
| 1-phosphatidylinositol-3-kinase activity | GO:0016303 | 0.00858346 | PIK3C2G, PIK3CG |
| negative thymic T cell selection | GO:0045060 | 0.00858346 | PTPRC, CCR7 |
| bleb assembly | GO:0032060 | 0.00858346 | ANO6, MYLK |
| protein transmembrane transporter activity | GO:0008320 | 0.00858346 | SEC61G, SEC63 |
| negative regulation of cAMP-dependent protein kinase activity | GO:2000480 | 0.00858346 | SIRT1, PRKAR2B |
| amyloid-beta binding | GO:0001540 | 0.008911339 | SCARB1, CRYAB, APBA2 |
| G2/M transition of mitotic cell cycle | GO:0000086 | 0.008911339 | USH1C, FBXL21, TPD52L1 |
| protein N-linked glycosylation | GO:0006487 | 0.009681093 | GFPT2, DAD1, GORASP1 |
| peptide hormone binding | GO:0017046 | 0.009681093 | GALR1, |

| | | | |
|---|---|---|---|
| | | | ADCYAP1R1, GHRHR-LR |
| negative regulation of androgen receptor signaling pathway | GO:0060766 | 0.010212247 | SIRT1, NCOR2 |
| auditory receptor cell stereocilium organization | GO:0060088 | 0.010212247 | WHRN, SEC24B |
| Arp2/3 complex binding | GO:0071933 | 0.010212247 | SNX9, GMFB |
| basement membrane assembly | GO:0070831 | 0.010212247 | LAMB1, LAMB4 |
| regulation of peptidyl-tyrosine phosphorylation | GO:0050730 | 0.010212247 | EPHA7, IL7 |
| phosphatidylinositol-mediated signaling | GO:0048015 | 0.010488406 | PLCB4, PIK3C2G, PIK3CG |
| positive regulation of MAP kinase activity | GO:0043406 | 0.010488406 | TENM1, PDE5A, PIK3CG |
| calcium-mediated signaling using intracellular calcium source | GO:0035584 | 0.011966131 | PTGFR, PTPRC |
| GABA-gated chloride ion channel activity | GO:0022851 | 0.011966131 | GABRA5, GABRB3 |
| lens fiber cell differentiation | GO:0070306 | 0.011966131 | CASP9, SMAD3 |
| stereocilium tip | GO:0032426 | 0.011966131 | USH1C, WHRN |
| cysteine-type endopeptidase activity involved in apoptotic process | GO:0097153 | 0.011966131 | GH, CASP9 |
| NAD+ binding | GO:0070403 | 0.011966131 | SIRT3, SIRT1 |
| positive regulation of gluconeogenesis | GO:0045722 | 0.011966131 | SIRT1, PPARA |
| nucleoplasm | GO:0005654 | 0.012302238 | NFATC2, MGMT, SMAD3, STXBP1, IKZF5, GABRA5, FZD10, BOC, GPRC5B, HSPH1, EIF3A, |

| | | | |
|---|---|---|---|
| | | | CGGBP1, NPAS2, UBQLN4, ARL14EP, MAGI1, TOP2A, SIRT1, FAF1, CTSC, LDB2, PPARA, BACH2, SDCBP, URI1, PTPN13, CCND1, RUNX1, ACTR8, SPATA2, YIPF5 |
| response to bacterium | GO:0009617 | 0.012520936 | PENK, HMCN1, CD79B, FUCA2 |
| response to drug | GO:0042493 | 0.012520936 | CCND1, NFATC2, VAV3, HTR2C |
| lysosome | GO:0005764 | 0.01307373 | RRAGA, WDR48, CTSC, NCSTN, RAB38, FUCA2 |
| cellular protein localization | GO:0034613 | 0.013138759 | FRMD6, RRAGA, GRID2 |
| phosphatidylinositol-3-phosphate biosynthetic process | GO:0036092 | 0.013841602 | PIK3C2G, PIK3CG |
| cell-cell junction organization | GO:0045216 | 0.013841602 | LIMS1, SMAD3 |
| integral component of peroxisomal membrane | GO:0005779 | 0.013841602 | PEX3, FAR1 |
| neuron projection extension | GO:1990138 | 0.013841602 | PRKCZ, |

| | | | |
|---|---|---|---|
| | | | IQGAP3 |
| apoptotic process | GO:0006915 | 0.014051569 | EPHA7, GH, CTSC, DAD1, CASP9 |
| regulation of circadian rhythm | GO:0042752 | 0.01409921 | FBXW7, TOP2A, PPARA |
| protein destabilization | GO:0031648 | 0.015098513 | SIRT1, CDC73, FBXW7 |
| mitotic cytokinesis | GO:0000281 | 0.015098513 | SNX9, CHMP4B, SPTBN1 |
| protein polyubiquitination | GO:0000209 | 0.015098521 | RNF114, TRIM2, TNKS2, UBE2W, SASH1 |
| regulation of membrane potential | GO:0042391 | 0.015144319 | GABRA5, GABRB3, GABRQ, CHRNA1 |
| intracellular membrane-bounded organelle | GO:0043231 | 0.015273167 | CYP7A1, ARL14EP, SCARB1, COMT, FAR1, RPIA, ADCYAP1 R1, GPRC5B |
| protein homodimerization activity | GO:0042803 | 0.01539484 | SNX9, CHMP4B, CRYAB, TENM1, SMAD3, TRIM9, APPL2, XPNPEP1, TPD52L1, SKI, ANO6, TOP2A |
| positive regulation of neural precursor cell | GO:2000179 | 0.015835221 | TOX, DLL4 |

| | | | |
|---|---|---|---|
| proliferation | | | |
| negative regulation of signal transduction | GO:0009968 | 0.015835221 | RGS20, UBASH3B |
| heterotypic cell-cell adhesion | GO:0034113 | 0.015835221 | CD200, PTPRC |
| S100 protein binding | GO:0044548 | 0.015835221 | CALD1, IQGAP3 |
| receptor complex | GO:0043235 | 0.016557362 | IL13RA2, KCTD16, EPHA7, SMAD3, ADCYAP1R1, GPRC5B |
| G protein-coupled peptide receptor activity | GO:0008528 | 0.017214324 | GALR1, ADCYAP1R1, GHRHR-LR |
| regulation of GTPase activity | GO:0043087 | 0.017214324 | CHN1, VAV3, IQGAP3 |
| hydrolase activity, hydrolyzing O-glycosyl compounds | GO:0004553 | 0.017943619 | PGGHG, ABHD10 |
| positive regulation of DNA replication | GO:0045740 | 0.017943619 | FAF1, KAT7 |
| T cell proliferation | GO:0042098 | 0.017943619 | PTPRC, NCSTN |
| calcium ion import | GO:0070509 | 0.017943619 | CACNA1D, CASK |
| sperm axoneme assembly | GO:0007288 | 0.017943619 | CFAP44, SPAG16 |
| steroid hormone mediated signaling pathway | GO:0043401 | 0.017943619 | BMP7, PPARA |
| alpha-actinin binding | GO:0051393 | 0.017943619 | CACNA1D, MAGI1 |
| heart looping | GO:0001947 | 0.019487205 | TBX20, VANGL2, SMAD3 |
| ribonucleoprotein complex | GO:1990904 | 0.019687691 | DDX17, NFATC2, IQGAP3, TOP2A |
| regulation of gene expression | GO:0010468 | 0.019805174 | MECOM, CGGBP1, |

| | | | |
|---|---|---|---|
| | | | APBA2, MAPK8, PRDM11 |
| negative regulation of neuron death | GO:1901215 | 0.020163492 | CHMP4B, SIRT1 |
| nuclear chromosome | GO:0000228 | 0.020163492 | FIGNL1, TOP2A |
| biological_process | GO:0008150 | 0.020163492 | LDB2, CDPF1 |
| spectrin binding | GO:0030507 | 0.020163492 | USH1C, PTPRC |
| site of DNA damage | GO:0090734 | 0.020163492 | UBQLN4, KAT7 |
| platelet aggregation | GO:0070527 | 0.020163492 | STXBP1, UBASH3B |
| positive regulation of neuron apoptotic process | GO:0043525 | 0.020163492 | EPHA7, CASP9 |
| postsynaptic density | GO:0014069 | 0.020453733 | EIF3A, PLCB4, ITPR1, DCLK1, SPTBN1 |
| negative regulation of neuron apoptotic process | GO:0043524 | 0.02136943 | GABRA5, DNAJC5, GABRB3, STXBP1 |
| regulation of cell cycle | GO:0051726 | 0.02136943 | MECOM, PTPRC, FBXL21, FIGNL1 |
| manganese ion binding | GO:0030145 | 0.021917747 | GALNT1, PEPD, XPNPEP1 |
| innervation | GO:0060384 | 0.022491602 | GABRA5, GABRB3 |
| voltage-gated ion channel activity | GO:0005244 | 0.022491602 | SCN4A, CLIC6 |
| nuclear hormone receptor binding | GO:0035257 | 0.022491602 | SIRT1, NCOR2 |
| homeostasis of number of cells | GO:0048872 | 0.022491602 | WDR48, CCR7 |
| neuronal action potential | GO:0019228 | 0.022491602 | SCN4A, CHRNA1 |
| histone deacetylase binding | GO:0042826 | 0.023136588 | CCND1, |

| | | | |
|---|---|---|---|
| | | | MAPK8, NCOR2, TOP2A |
| modulation of chemical synaptic transmission | GO:0050804 | 0.023192219 | PRKAR2B, PLCB4, GRID2 |
| adenylate cyclase-modulating G protein-coupled receptor signaling pathway | GO:0007188 | 0.023192219 | ADCYAP1R1, CACNA1D, GHRHR-LR |
| stress fiber | GO:0001725 | 0.023192219 | VANGL2, MYLK, LPP |
| negative regulation of transcription, DNA-templated | GO:0045892 | 0.023369781 | BMP7, SIRT1, TBL1X, MECOM, CRYAB, KAT7, LDB2 |
| axon | GO:0030424 | 0.023500443 | COMT, STXBP1, CD200, VSTM5, SCN4A, KCNA4 |
| proteolysis | GO:0006508 | 0.023662093 | AMZ1, CTRC, PEPD, CASP9, MIPEP, ADAM33, TMPRSS7, XPNPEP1 |
| cytoplasmic vesicle membrane | GO:0030659 | 0.024506153 | SNX9, GABRB3, ITPR1 |
| Schaffer collateral - CA1 synapse | GO:0098685 | 0.024506153 | ITPR1, CASK, APBA2 |
| cyclin binding | GO:0030332 | 0.024924777 | TOP2A, FBXW7 |
| helicase activity | GO:0004386 | 0.024924777 | DHX8, DHX37 |
| gamma-aminobutyric acid signaling | GO:0007214 | 0.024924777 | GABRA5, |

| | | | |
|---|---|---|---|
| pathway | | | GABRB3 |
| neuromuscular synaptic transmission | GO:0007274 | 0.024924777 | STXBP1, CHRNA1 |
| embryonic pattern specification | GO:0009880 | 0.024924777 | BMP7, SMAD3 |
| regulation of postsynapse organization | GO:0099175 | 0.024924777 | EPHA7, ARHGAP22 |
| neuropeptide signaling pathway | GO:0007218 | 0.024990063 | GALR1, PENK, TENM1, LGALSL |
| RNA polymerase II activating transcription factor binding | GO:0001102 | 0.025859516 | LDB2, TBX20, SMAD3 |
| phosphatidylinositol phosphorylation | GO:0046854 | 0.025859516 | PIK3C2G, TTC7A, PIK3CG |
| cellular response to DNA damage stimulus | GO:0006974 | 0.026144482 | CCND1, NFATC2, VAV3, UBQLN4, TOP2A |
| dendrite | GO:0030425 | 0.026622925 | PLCB4, COMT, TRIM9, HTR2C, VSTM5, PENK |
| transcription corepressor activity | GO:0003714 | 0.026919936 | CCND1, SIRT1, TBL1X, NCOR2, URI1 |
| positive regulation of cell migration | GO:0030335 | 0.026919936 | LAMB1, TBX20, SDCBP, MYLK, SEMA5B |
| signaling receptor activity | GO:0038023 | 0.02693064 | GRIA2, GRIA3, TNFRSF19, GRID2 |
| DNA-binding transcription factor activity | GO:0003700 | 0.027183312 | NFATC2, DMRTA2, |

| | | | |
|---|---|---|---|
| | | | BACH2, NPAS2, SMAD3, MECOM, IKZF5 |
| metalloaminopeptidase activity | GO:0070006 | 0.02745991 | PEPD, XPNPEP1 |
| ephrin receptor binding | GO:0046875 | 0.02745991 | CHN1, PIK3CG |
| inner ear receptor cell stereocilium organization | GO:0060122 | 0.02745991 | USH1C, VANGL2 |
| AMPA glutamate receptor complex | GO:0032281 | 0.02745991 | GRIA2, GRIA3 |
| positive regulation of DNA repair | GO:0045739 | 0.02745991 | SIRT1, NPAS2 |
| cellular response to ionizing radiation | GO:0071479 | 0.02745991 | SIRT1, FIGNL1 |
| trans-Golgi network | GO:0005802 | 0.027709906 | TRAPPC9, TGFBI, SNX9, GH, RAB38 |
| circadian regulation of gene expression | GO:0032922 | 0.028684296 | SIRT1, NPAS2, PPARA |
| flavin adenine dinucleotide binding | GO:0050660 | 0.028684296 | CHDH, ACADSB, ACOX3 |
| nuclear envelope | GO:0005635 | 0.028958996 | FAF1, CHMP4B, TNKS2, PRKCZ |
| protein kinase binding | GO:0019901 | 0.029692875 | IQGAP3, FAF1, CASP9, CCND1, PTPRC, SMAD3, SKI, GPRC5B |
| cysteine-type endopeptidase activity | GO:0004197 | 0.030155546 | CTSC, USP46, USP15 |
| GTPase activator activity | GO:0005096 | 0.031439199 | TBC1D8, ARHGAP22 |

| | | | |
|---|---|---|---|
| | | | , CHN1, STXBP5, RASA3, IQGAP3 |
| stereocilium | GO:0032420 | 0.032823922 | USH1C, WHRN |
| endoplasmic reticulum exit site | GO:0070971 | 0.032823922 | YIPF5, SEC24B |
| negative regulation of intrinsic apoptotic signaling pathway | GO:2001243 | 0.032823922 | URI1, FIGNL1 |
| Notch binding | GO:0005112 | 0.032823922 | DLL4, NCOR2 |
| GTPase binding | GO:0051020 | 0.032823922 | RRAGA, SPTBN1 |
| fat cell differentiation | GO:0045444 | 0.033215202 | CCND1, TBL1X, CBY1 |
| cellular response to starvation | GO:0009267 | 0.034803327 | SIRT1, RRAGA, PPARA |
| axon extension | GO:0048675 | 0.035646894 | DCLK1, SEMA5B |
| negative regulation of Notch signaling pathway | GO:0045746 | 0.035646894 | BMP7, DLL4 |
| positive regulation of Wnt signaling pathway | GO:0030177 | 0.035646894 | CDC73, DIXDC1 |
| platelet-derived growth factor receptor signaling pathway | GO:0048008 | 0.035646894 | CSRNP1, IQGAP3 |
| protein deubiquitination | GO:0016579 | 0.036430102 | JOSD1, WDR48, USP46 |
| positive regulation of cytosolic calcium ion concentration | GO:0007204 | 0.036430102 | GALR1, PTGFR, CCR7 |
| growth factor activity | GO:0008083 | 0.037959954 | BMP7, IL7, GMFB, GH |
| cholesterol homeostasis | GO:0042632 | 0.038095344 | SCARB1, CYP7A1, SIRT1 |
| transmembrane signaling receptor activity | GO:0004888 | 0.038095344 | HMCN1, CD79B, CHRNA1 |
| negative regulation of protein-containing complex assembly | GO:0031333 | 0.038560004 | PRKCZ, CRYAB |

| | | | |
|---|---|---|---|
| establishment of protein localization | GO:0045184 | 0.038560004 | PHLDB2, WHRN |
| humoral immune response | GO:0006959 | 0.038560004 | IFNG, SH2D1A |
| phosphatidylinositol 3-kinase complex | GO:0005942 | 0.038560004 | PIK3C2G, PIK3CG |
| immunological synapse | GO:0001772 | 0.038560004 | CRTAM, VAV3 |
| bHLH transcription factor binding | GO:0043425 | 0.038560004 | SIRT1, SMAD3 |
| BMP signaling pathway | GO:0030509 | 0.039798855 | BMP7, USP15, SMAD3 |
| SMAD protein signal transduction | GO:0060395 | 0.039798855 | BMP7, SKI, SMAD3 |
| actin binding | GO:0003779 | 0.041344564 | CALD1, WIPF1, GMFB, SPTBN1, PHACTR2 |
| peroxisome | GO:0005777 | 0.041540421 | ACOX3, FAR1, TMEM135 |
| RNA polymerase II complex binding | GO:0000993 | 0.041560445 | URI1, CDC73 |
| U2-type catalytic step 2 spliceosome | GO:0071007 | 0.041560445 | DHX8, CRNKL1 |
| ureteric bud development | GO:0001657 | 0.041560445 | BMP7, SMAD3 |
| positive regulation of TOR signaling | GO:0032008 | 0.041560445 | RRAGA, LAMTOR2 |
| protein kinase activity | GO:0004672 | 0.041704028 | CCND1, DCLK1, MYLK, CASK |
| response to hypoxia | GO:0001666 | 0.043319815 | ITPR1, CRYAB, SMAD3 |
| ubiquitin protein ligase binding | GO:0031625 | 0.043584351 | FAF1, SNX9, SMAD3, PRKAR2B, SKI, FBXW7 |

| | | | |
|---|---|---|---|
| COPII-coated ER to Golgi transport vesicle | GO:0030134 | 0.044645469 | VANGL2, YIPF5 |
| histone deacetylation | GO:0016575 | 0.044645469 | SIRT3, TBL1X |
| photoreceptor connecting cilium | GO:0032391 | 0.044645469 | USH1C, WHRN |
| branching involved in blood vessel morphogenesis | GO:0001569 | 0.044645469 | TBX20, DLL4 |
| intra-Golgi vesicle-mediated transport | GO:0006891 | 0.044645469 | COPA, COG6 |
| cell leading edge | GO:0031252 | 0.044645469 | LDB2, PHLDB2 |
| positive regulation of protein phosphorylation | GO:0001934 | 0.045136795 | CCND1, EPHA7, PRR5 |
| regulation of postsynaptic membrane potential | GO:0060078 | 0.047812384 | GABRA5, GRIA3 |
| chemorepellent activity | GO:0045499 | 0.047812384 | EPHA7, SEMA5B |
| somatic stem cell population maintenance | GO:0035019 | 0.047812384 | LDB2, VANGL2 |
| tissue development | GO:0009888 | 0.047812384 | LAMB1, LAMB4 |
| skeletal system morphogenesis | GO:0048705 | 0.047812384 | WDR48, CSRNP1 |
| photoreceptor inner segment | GO:0001917 | 0.047812384 | USH1C, WHRN |
| cell adhesion molecule binding | GO:0050839 | 0.047812384 | TGFBI, TENM1 |
| regulation of microtubule cytoskeleton organization | GO:0070507 | 0.047812384 | PHLDB2, DIXDC1 |
| phosphatase binding | GO:0019902 | 0.047812384 | NFATC2, SMAD3 |
| RNA polymerase II cis-regulatory region sequence-specific DNA binding | GO:0000978 | 0.048515876 | NFATC2, AKNA, TBX20, NPAS2, SMAD3, RUNX1, PPARA, SIRT1, BACH2, SKI |
| basement membrane | GO:0005604 | 0.048882476 | LAMB1, |

| | | | |
|---|---|---|---|
| | | | HMCN1, CASK |
