# Supplementary material for: Genome-Wide Analysis Reveals Copy Number Variation-Mediated Adaptive Evolution in Chickens Under Diverse Environmental and Domestication Pressures
Source: Animals (Basel). 2026 Oct 29;16(21):3424. doi: 10.3390/ani16213424 (PMC13762271; doi:10.3390/ani16213424)
Supplement: Supplementary file 1 [file animals-16-03424-s001.zip › Table S9.pdf]

Table S9. KEGG pathways significantly enriched for genes overlapping candidate CNVRs under high-altitude adaptation selection.

Term	ID	P_Value	Matched_Genes
Neuroactive ligand-receptor interaction	gga04080	5.69468E-05	PTGFR, GABRB3, HTR2C, GRID2, GRIA2, GRIA3, GABRQ, GALR1, CHRNA1, GHRHR-LR, GABRA5, EDNRB, ADCYAP1R1, PENK, GH
Protein processing in endoplasmic reticulum	gga04141	0.000282292	HSPH1, DNAJC5, CRYAB, UBQLN4, SEC63, SEC24B, SEC61G, MAPK8, DAD1
PPAR signaling pathway	gga03320	0.000300872	ACOX3, FABP5, FABP2, UBB, PPARA, CYP7A1
Wnt signaling pathway	gga04310	0.000764809	NFATC2, PLCB4, VANGL2, CCND1, CBY1, TBL1X, FZD10, MAPK8
Tight junction	gga04530	0.001419381	MYL12B, MYL12A, CCND1, CACNA1D, RUNX1, MAGI1, MAPK8, PRKCZ
AGE-RAGE signaling pathway in diabetic complications	gga04933	0.001605782	PLCB4, CCND1, SMAD3, PRKCZ, MAPK8, IL1B
Focal adhesion	gga04510	0.004396868	MYL12B, MYL12A, CCND1, VAV3, MYLK, LAMB1, MAPK8, LAMB4
Vascular smooth muscle contraction	gga04270	0.005506976	PLCB4, CACNA1D, MYLK, GNA12, ITPR1, CALD1
Apelin signaling pathway	gga04371	0.005950018	PLCB4, CCND1, MYLK, PIK3CG, SMAD3, ITPR1
Cytokine-cytokine receptor interaction	gga04060	0.006409308	BMP7, IL13RA2, IFNG, IL7, CCR7, IL1B, TNFRSF19,

			GH
Phosphatidylinositol signaling system	gga04070	0.008416277	PLCB4, DGKH, ITPR1, PIK3C2G, SYNJ2
Necroptosis	gga04217	0.00917064	FAF1, CHMP4B, IFNG, SPATA2, MAPK8, IL1B
Calcium signaling pathway	gga04020	0.011470761	PTGFR, PLCB4, CACNA1D, MYLK, HTR2C, EDNRB, ITPR1
mTOR signaling pathway	gga04150	0.0122904	RRAGA, PRR5, ATP6V1H, ATP6V1G3, FZD10, LAMTOR2
Metabolic pathways	gga01100	0.013421429	B4GALNT4, ATP6V1H, DAD1, PIK3CG, PDE7A, DGKH, RPIA, PHOSPHO1, SYNJ2, CYP7A1, PDE5A, PLCB4, COMT, SUCLG1, PIK3C2G, ACADSB, AGMAT, DDC, CHDH, NT5DC4, GALNT1, HACD2, ACOX3, MTHFD1L, DLAT, ATP6V1G3, GFPT2
Cellular senescence	gga04218	0.013478224	NFATC2, CCND1, CACNA1D, SIRT1, SMAD3, ITPR1
Inositol phosphate metabolism	gga00562	0.016576317	PLCB4, PIK3CG, PIK3C2G, SYNJ2
Notch signaling pathway	gga04330	0.023192219	DLL4, NCOR2, NCSTN
GnRH signaling pathway	gga04912	0.027933808	PLCB4, MAPK8, ITPR1, CACNA1D
Apoptosis	gga04210	0.029333685	PTPN13, MAPK8, CTSC, ITPR1, CASP9
C-type lectin receptor signaling pathway	gga04625	0.031075698	NFATC2, ITPR1, MAPK8, IL1B
Fatty acid metabolism	gga01212	0.036430102	ACOX3, HACD2,

			ACADSB
Protein export	gga03060	0.041560445	SEC61G, SEC63
Regulation of actin cytoskeleton	gga04810	0.043584351	MYL12B, MYL12A, VAV3, MYLK, GNA12, IQGAP3
Carbon metabolism	gga01200	0.049796574	ACOX3, DLAT, RPIA, SUCLG1
