# Supplementary material for: Genome-Wide Analysis Reveals Copy Number Variation-Mediated Adaptive Evolution in Chickens Under Diverse Environmental and Domestication Pressures
Source: Animals (Basel). 2026 Oct 29;16(21):3424. doi: 10.3390/ani16213424 (PMC13762271; doi:10.3390/ani16213424)
Supplement: Supplementary file 1 [file animals-16-03424-s001.zip › Table S8.pdf]

Table S8. Candidate CNVRs and annotated genes under positive selection in the high-altitude adaptation group.

CHROM	BIN_START	BIN_END	Gene_Name
1	106080001	106120000	CLIC6
1	106080001	106120000	RUNX1
1	111860001	111900000	CASK
1	113040001	113080000	RPGR
1	118540001	118580000	gene:ENSGALG00010003247
1	118560001	118600000	gene:ENSGALG00010003247
1	125400001	125440000	TBL1X
1	125420001	125460000	TBL1X
1	12660001	12700000	PHTF2
1	126640001	126680000	PUDP
1	12680001	12720000	PHTF2
1	12680001	12720000	TMEM60
1	12680001	12720000	RSBN1L
1	131380001	131420000	GABRA5
1	131380001	131420000	GABRB3
1	131400001	131440000	GABRA5
1	131400001	131440000	GABRB3
1	133460001	133500000	NPAS2
1	133460001	133500000	RPL31
1	133460001	133500000	TBC1D8
1	133480001	133520000	RPL31
1	133480001	133520000	TBC1D8
1	136400001	136440000	LIMS1
1	136400001	136440000	RANBP2
1	136420001	136460000	LIMS1
1	136420001	136460000	RANBP2
1	136960001	137000000	CDC16
1	136960001	137000000	RASA3
1	14600001	14640000	PIK3CG
1	14620001	14660000	PIK3CG
1	14620001	14660000	PRKAR2B
1	15080001	15120000	LAMB1
1	15080001	15120000	LAMB4
1	15100001	15140000	LAMB1
1	15100001	15140000	LAMB4
1	153860001	153900000	EDNRB
1	153920001	153960000	SLAIN1
1	166160001	166200000	DGKH
1	166180001	166220000	DGKH
1	166200001	166240000	DGKH

1	166220001	166260000	DGKH
1	170780001	170820000	COG6
1	170800001	170840000	COG6
1	172660001	172700000	DCLK1
1	174860001	174900000	HSPH1
1	175200001	175240000	UBL3
1	175220001	175260000	UBL3
1	175320001	175360000	gene:ENSGALG00010007162
1	175340001	175380000	gene:ENSGALG00010007162
1	177340001	177380000	MIPEP
1	177340001	177380000	TNFRSF19
1	185620001	185660000	TM4SF1a
1	185620001	185660000	gene:ENSGALG00010008694
1	185620001	185660000	VSTM5
1	185640001	185680000	gene:ENSGALG00010008694
1	185640001	185680000	VSTM5
1	185640001	185680000	MED17
1	185640001	185680000	C11orf54
1	188140001	188180000	CTSC
1	188160001	188200000	CTSC
1	188160001	188200000	RAB38
1	188540001	188580000	TMEM135
1	188560001	188600000	TMEM135
1	29280001	29320000	PDZRN4
1	29300001	29340000	PDZRN4
1	30780001	30820000	ANO6
1	31560001	31600000	RPAP3
1	31560001	31600000	ATP23
1	31580001	31620000	RPAP3
1	31580001	31620000	ATP23
1	32980001	33020000	TAFA2
1	33000001	33040000	TAFA2
1	33000001	33040000	USP15
1	35160001	35200000	IFNG
1	50880001	50920000	JOSD1
1	50880001	50920000	TOMM22
1	50880001	50920000	CBY1
1	50880001	50920000	DMC1
1	50880001	50920000	DDX17
1	54220001	54260000	C12orf75
1	54220001	54260000	APPL2
1	54240001	54280000	C12orf75
1	54240001	54280000	APPL2

1	54580001	54620000	CHST11
1	62400001	62440000	CALD1
1	62420001	62460000	CALD1
1	64060001	64100000	PIK3C2G
1	69780001	69820000	PRR5
1	69800001	69840000	PRR5
1	71340001	71380000	PPARA
1	71340001	71380000	CDPF1
1	71360001	71400000	CDPF1
1	71360001	71400000	PKDREJ
1	71360001	71400000	gene:ENSGALG00010026220
1	78020001	78060000	gene:ENSGALG00010023158
1	78020001	78060000	gene:ENSGALG00010023251
1	83480001	83520000	ILDR1
1	83480001	83520000	CFAP44
1	83500001	83540000	CFAP44
1	83500001	83540000	BOC
1	87940001	87980000	GUCA1C
1	88760001	88800000	PHLDB2
1	88760001	88800000	ABHD10
1	88760001	88800000	TAGLN3
1	88760001	88800000	TMPRSS7
1	88940001	88980000	CD200
1	88960001	89000000	CGGBP1
1	88960001	89000000	ZNF654
1	99480001	99520000	C21orf91
2	100460001	100500000	MYL12B
2	100480001	100520000	MYL12B
2	100480001	100520000	MYL12A
2	109960001	110000000	ATP6V1H
2	109960001	110000000	RGS20
2	111040001	111080000	ERD2L
2	111040001	111080000	PENK
2	111060001	111100000	ERD2L
2	111060001	111100000	PENK
2	111720001	111760000	UBXN2B
2	111720001	111760000	CYP7A1
2	111720001	111760000	SDCBP
2	111880001	111920000	TOX
2	114560001	114600000	PDE7A
2	114580001	114620000	PDE7A
2	117640001	117680000	STAU2
2	117640001	117680000	UBE2W

2	117660001	117700000	STAU2
2	117660001	117700000	UBE2W
2	120040001	120080000	IL7
2	120920001	120960000	MRPL53
2	120920001	120960000	FABP5
2	120940001	120980000	MRPL53
2	120940001	120980000	FABP5
2	144340001	144380000	TRAPPC9
2	144360001	144400000	TRAPPC9
2	147280001	147320000	LY6CLEL
2	147280001	147320000	gene:ENSGALG00010008048
2	147280001	147320000	gene:ENSGALG00010008053
2	1500001	1540000	ADCYAP1R1
2	1500001	1540000	GHRHR-LR
2	1520001	1560000	GHRHR-LR
2	17240001	17280000	OTUD1
2	17260001	17300000	OTUD1
2	47120001	47160000	TBX20
2	47140001	47180000	TBX20
2	52100001	52140000	SEC61G
2	52120001	52160000	SEC61G
2	5260001	5300000	CSRNP1
2	5260001	5300000	GORASP1
2	5260001	5300000	WDR48
2	80360001	80400000	FIGNL1
2	80360001	80400000	DDC
2	80380001	80420000	DDC
2	83400001	83440000	GALNT1
2	89980001	90020000	GALR1
3	105960001	106000000	HMBOX1
3	105980001	106020000	HMBOX1
3	106760001	106800000	XKR6
3	13700001	13740000	PLCB4
3	13720001	13760000	PLCB4
3	23000001	23040000	PKDCC
3	23020001	23060000	PKDCC
3	26700001	26740000	TTC7A
3	26720001	26760000	TTC7A
3	2920001	2960000	SPTBN1
3	31100001	31140000	TTC27
3	31120001	31160000	TTC27
3	4060001	4100000	CFAP61
3	4080001	4120000	CFAP61

3	4080001	4120000	CRNKL1
3	46880001	46920000	STXBP5
3	46900001	46940000	SASH1
3	48340001	48380000	MTHFD1L
3	48360001	48400000	MTHFD1L
3	51420001	51460000	SNX9
3	51440001	51480000	SNX9
3	51440001	51480000	SYNJ2
3	52120001	52160000	PHACTR2
3	52120001	52160000	FUCA2
3	52120001	52160000	PEX3
3	52120001	52160000	ADAT2
3	52140001	52180000	PEX3
3	52140001	52180000	ADAT2
3	59700001	59740000	HDDC2
3	59700001	59740000	TPD52L1
3	59720001	59760000	TPD52L1
3	59740001	59780000	TPD52L1
3	67300001	67340000	SEC63
3	67300001	67340000	SCML4
3	67320001	67360000	SCML4
3	67380001	67420000	SOBP
3	67400001	67440000	SOBP
3	72160001	72200000	MMS22L
3	72180001	72220000	MMS22L
3	72180001	72220000	gene:ENSGALG00010004383
3	73640001	73680000	EPHA7
3	73660001	73700000	EPHA7
3	75000001	75040000	BACH2
3	79140001	79180000	HMGN2P46
3	86020001	86060000	KHDRBS2
3	86040001	86080000	KHDRBS2
3	91780001	91820000	TMEM18
3	9880001	9920000	LGALSL
3	9900001	9940000	LGALSL
4	10580001	10620000	GABRQ
4	11400001	11440000	gene:ENSGALG00010011823
4	11400001	11440000	KLF8
4	11400001	11440000	RRAGA
4	11420001	11460000	KLF8
4	11420001	11460000	RRAGA
4	15460001	15500000	TENM1
4	15460001	15500000	SH2D1A

4	15480001	15520000	TENM1
4	15480001	15520000	SH2D1A
4	15840001	15880000	GRIA3
4	18520001	18560000	FMR1NB
4	19240001	19280000	TRIM2
4	19260001	19300000	TRIM2
4	20860001	20900000	GRIA2
4	2740001	2780000	HTR2C
4	2760001	2800000	HTR2C
4	2760001	2800000	IL13RA2
4	33260001	33300000	FBXW7
4	33280001	33320000	FBXW7
4	33280001	33320000	gene:ENSGALG00010009088
4	34420001	34460000	gene:ENSGALG00010008152
4	36320001	36360000	GRID2
4	36340001	36380000	GRID2
4	36360001	36400000	GRID2
4	36800001	36840000	OPN4-1
4	36820001	36860000	OPN4-1
4	36820001	36860000	SEC24B
4	45420001	45460000	PTPN13
4	45440001	45480000	PTPN13
4	54200001	54240000	PDE5A
4	54200001	54240000	FABP2
4	54200001	54240000	gene:ENSGALG00010005572
4	54220001	54260000	PDE5A
4	54220001	54260000	FABP2
4	54220001	54260000	gene:ENSGALG00010005572
4	54220001	54260000	USP53
4	65500001	65540000	USP46
4	75880001	75920000	LDB2
4	75900001	75940000	LDB2
4	80480001	80520000	ACOX3
4	80480001	80520000	TRMT44
4	80500001	80540000	TRMT44
4	85800001	85840000	RPIA
4	85800001	85840000	SUCLG1
4	85820001	85860000	SUCLG1
4	89180001	89220000	ADAM33
4	89180001	89220000	gene:ENSGALG00010018087
5	11520001	11560000	ABCC8
5	11540001	11580000	ABCC8
5	11540001	11580000	USH1C

5	1280001	1320000	LUZP2
5	1300001	1340000	LUZP2
5	17060001	17100000	CCND1
5	17060001	17100000	ORAOV1
5	1740001	1780000	SIGIRR
5	1740001	1780000	PKP3
5	1740001	1780000	B4GALNT4
5	1740001	1780000	gene:ENSGALG00010020824
5	1740001	1780000	IFITM5
5	1760001	1800000	B4GALNT4
5	1760001	1800000	gene:ENSGALG00010020824
5	1760001	1800000	IFITM5
5	1760001	1800000	PGGHG
5	1760001	1800000	gene:ENSGALG00010020854
5	1760001	1800000	PSMD13
5	1760001	1800000	SIRT3
5	21680001	21720000	TSPAN18
5	21680001	21720000	TP53I11
5	21700001	21740000	TSPAN18
5	21700001	21740000	TP53I11
5	21780001	21820000	PRDM11
5	23280001	23320000	PHF21A
5	23300001	23340000	PHF21A
5	23900001	23940000	DLL4
5	2920001	2960000	ANO5
5	2940001	2980000	ANO5
5	3440001	3480000	ANO3
5	3460001	3500000	ANO3
5	45040001	45080000	SPIA4
5	45040001	45080000	SPIA1
5	4740001	4780000	KCNA4
5	4740001	4780000	ARL14EP
5	4760001	4800000	ARL14EP
5	4760001	4800000	MPPED2
5	52140001	52180000	BRF1
5	52160001	52200000	BRF1
5	56060001	56100000	GMFB
5	56060001	56100000	CNIH1
5	56060001	56100000	CDKN3
5	57760001	57800000	TRIM9
5	57760001	57800000	TMX1
5	57780001	57820000	TMX1
5	57780001	57820000	FRMD6

5	5820001	5860000	QSER1
5	5840001	5880000	QSER1
5	5840001	5880000	gene:ENSGALG00010023037
5	7140001	7180000	FAR1
5	9380001	9420000	DENND2B
5	9380001	9420000	RPL27A
5	9380001	9420000	TRIM66
5	9400001	9440000	DENND2B
5	9400001	9440000	RPL27A
5	9400001	9440000	TRIM66
5	9780001	9820000	LMO1
6	19020001	19060000	MAPK8
6	19020001	19060000	ARHGAP22
6	19040001	19080000	MAPK8
6	19040001	19080000	ARHGAP22
6	20520001	20560000	HECTD2
6	20540001	20580000	HECTD2
6	20620001	20660000	PPP1R3C
6	20620001	20660000	TNKS2
6	20640001	20680000	TNKS2
6	26440001	26480000	XPNPEP1
6	26460001	26500000	XPNPEP1
6	30720001	30760000	NANOS1
6	30720001	30760000	EIF3A
6	30740001	30780000	NANOS1
6	30740001	30780000	EIF3A
6	32180001	32220000	CUZD1
6	32180001	32220000	C10orf88
6	32180001	32220000	gene:ENSGALG00010001764
6	32180001	32220000	IKZF5
6	32180001	32220000	PSTK
6	32180001	32220000	ACADSB
6	32200001	32240000	gene:ENSGALG00010001764
6	32200001	32240000	IKZF5
6	32200001	32240000	PSTK
6	32200001	32240000	ACADSB
6	34080001	34120000	PTPRE
6	34100001	34140000	PTPRE
6	34820001	34860000	MGMT
6	7280001	7320000	PCDH15
6	7280001	7320000	SIRT1
6	9860001	9900000	gene:ENSGALG00010008686
7	13800001	13840000	ITPRID2

7	16380001	16420000	CHN1
7	16400001	16440000	CHN1
7	16400001	16440000	CHRNA1
7	16400001	16440000	WIPF1
7	26160001	26200000	SEMA5B
7	26760001	26800000	ADCY6
7	26760001	26800000	HACD2
7	26760001	26800000	MYLK
7	26780001	26820000	HACD2
7	26780001	26820000	MYLK
7	3860001	3900000	SPAG16
7	3880001	3920000	SPAG16
8	10360001	10400000	HMCN1
8	10380001	10420000	HMCN1
8	1060001	1100000	VAV3
8	14000001	14040000	BTBD8
8	14000001	14040000	EPHX3
8	14000001	14040000	BRDT
8	14020001	14060000	EPHX3
8	14020001	14060000	BRDT
8	18420001	18460000	PTGFR
8	20400001	20440000	RNF220
8	20400001	20440000	TMEM53
8	20400001	20440000	ARMH1
8	20400001	20440000	KIF2C
8	2080001	2120000	PTPRC
8	2080001	2120000	ATP6V1G3
8	2100001	2140000	ATP6V1G3
8	23440001	23480000	DMRTA2
8	23460001	23500000	DMRTA2
8	23460001	23500000	FAF1
8	2680001	2720000	gene:ENSGALG00010012436
8	2680001	2720000	KCNT2
8	2700001	2740000	KCNT2
8	3360001	3400000	CDC73
8	3380001	3420000	CDC73
8	6280001	6320000	RALGPS2
8	6300001	6340000	RALGPS2
8	6300001	6340000	ANGPTL1
9	14220001	14260000	LPP
9	19740001	19780000	MECOM
10	18780001	18820000	SMAD3
10	5860001	5900000	APBA2

11	8140001	8180000	URI1
11	8160001	8200000	URI1
11	9980001	10020000	PEPD
12	14460001	14500000	MAGI1
12	14760001	14800000	KBTBD8
12	14780001	14820000	KBTBD8
12	14780001	14820000	gene:ENSGALG00010019696
12	17140001	17180000	CNTN3
12	17160001	17200000	CNTN3
12	18580001	18620000	SUMF1
12	18580001	18620000	ITPR1
12	18600001	18640000	ITPR1
12	7360001	7400000	CACNA1D
12	7360001	7400000	CHDH
12	7380001	7420000	CACNA1D
12	7380001	7420000	CHDH
12	7380001	7420000	ACTR8
13	13600001	13640000	GFPT2
13	13620001	13660000	RASGEF1C
13	14780001	14820000	TGFBI
13	14780001	14820000	LECT2
13	14800001	14840000	LECT2
13	14800001	14840000	FBXL21
13	17120001	17160000	YIPF5
13	17140001	17180000	YIPF5
13	17140001	17180000	KCTD16
14	11480001	11520000	RBFOX1
14	11500001	11540000	RBFOX1
14	13640001	13680000	gene:ENSGALG00010018881
14	13640001	13680000	JPT2
14	13660001	13700000	gene:ENSGALG00010018881
14	13660001	13700000	JPT2
14	13660001	13700000	CRAMP1
14	14740001	14780000	GPRC5B
14	14760001	14800000	GPRC5B
14	14760001	14800000	gene:ENSGALG00010017649
14	14760001	14800000	gene:ENSGALG00010017653
14	2300001	2340000	C7orf50
14	2300001	2340000	GPR146
14	2320001	2360000	C7orf50
14	2320001	2360000	GPR146
14	2320001	2360000	gene:ENSGALG00010022470
14	3360001	3400000	AMZ1

14	3360001	3400000	GNA12
14	3380001	3420000	GNA12
14	5840001	5880000	LMF1
14	5860001	5900000	LMF1
14	7080001	7120000	HS3ST4
14	7100001	7140000	HS3ST4
14	7120001	7160000	HS3ST4
15	1080001	1120000	COMT
15	1080001	1120000	ARVCF
15	1100001	1140000	ARVCF
15	1360001	1400000	ZDHHC8
15	1820001	1860000	FBRSL1
15	1880001	1920000	FBRSL1
15	3440001	3480000	FZD10
15	4520001	4560000	DHX37
15	4520001	4560000	UBB
15	4520001	4560000	SCARB1
15	4540001	4580000	SCARB1
15	4540001	4580000	NCOR2
17	3120001	3160000	STXBP1
17	3120001	3160000	AKNA
17	3120001	3160000	WHRN
19	3480001	3520000	SPNS2
19	3500001	3540000	SPNS2
20	12040001	12080000	BMP7
20	13540001	13580000	NFATC2
20	13560001	13600000	NFATC2
20	14140001	14180000	RNF114
20	14140001	14180000	SPATA2
20	14140001	14180000	gene:ENSGALG00010013141
20	14140001	14180000	SLC9A8
20	2400001	2440000	gene:ENSGALG00010024375
20	2420001	2460000	CHMP4B
20	9860001	9900000	DNAJC5
20	9860001	9900000	TPD52L2
20	9880001	9920000	TPD52L2
20	9880001	9920000	ZBTB46
21	1720001	1760000	SKI
21	1740001	1780000	SKI
21	1760001	1800000	SKI
21	1760001	1800000	gene:ENSGALG00010020048
21	1760001	1800000	PRKCZ
21	4900001	4940000	PINK1

21	4900001	4940000	AGMAT
21	4900001	4940000	DNAJC16
21	4900001	4940000	CASP9
21	4900001	4940000	CTRC
21	4900001	4940000	EFHD2
21	4900001	4940000	FHAD1
22	4420001	4460000	gene:ENSGALG00010018371
22	4420001	4460000	SLC20A1
22	4420001	4460000	NT5DC4
22	4420001	4460000	gene:ENSGALG00010018401
22	4420001	4460000	IL1B
24	3180001	3220000	CRTAM
24	3180001	3220000	UBASH3B
24	3200001	3240000	UBASH3B
24	6220001	6260000	DLAT
24	6220001	6260000	DIXDC1
24	6220001	6260000	C11ORF52
24	6220001	6260000	CRYAB
25	1220001	1260000	COPA
25	1220001	1260000	VANGL2
25	1220001	1260000	NHLH1
25	1220001	1260000	NCSTN
25	1880001	1920000	TPM3
25	1880001	1920000	C1orf43
25	1880001	1920000	UBAP2L
25	400001	440000	LAMTOR2
25	400001	440000	UBQLN4
25	400001	440000	gene:ENSGALG00010028085
25	400001	440000	NAXE
25	400001	440000	gene:ENSGALG00010028094
25	400001	440000	TTC24
25	420001	460000	gene:ENSGALG00010028085
25	420001	460000	NAXE
25	420001	460000	gene:ENSGALG00010028094
25	420001	460000	TTC24
25	420001	460000	IQGAP3
26	3720001	3760000	MAGI3
26	3720001	3760000	PHTF1
26	3740001	3780000	MAGI3
26	3740001	3780000	PHTF1
26	3740001	3780000	RSBN1
27	100001	140000	gene:ENSGALG00010022054
27	100001	140000	DAD1

27	100001	140000	gene:ENSGALG00010021157
27	120001	160000	gene:ENSGALG00010021157
27	1260001	1300000	RDM1
27	1260001	1300000	GH
27	1260001	1300000	CD79B
27	1260001	1300000	SCN4A
27	2880001	2920000	KAT7
27	3180001	3220000	DHX8
27	3180001	3220000	PHB1
27	3180001	3220000	ZNF652
27	3200001	3240000	ZNF652
27	3200001	3240000	PHOSPHO1
27	4420001	4460000	TOP2A
27	4420001	4460000	gene:ENSGALG00010022502
27	4420001	4460000	IGFBP4
27	4420001	4460000	TNS4
27	4440001	4480000	gene:ENSGALG00010022502
27	4440001	4480000	IGFBP4
27	4440001	4480000	TNS4
27	4440001	4480000	CCR7
