# Supplementary material for: Genome-Wide Analysis Reveals Copy Number Variation-Mediated Adaptive Evolution in Chickens Under Diverse Environmental and Domestication Pressures
Source: Animals (Basel). 2026 Oct 29;16(21):3424. doi: 10.3390/ani16213424 (PMC13762271; doi:10.3390/ani16213424)
Supplement: Supplementary file 1 [file animals-16-03424-s001.zip › Table S7.pdf]

Table S7. GO terms significantly enriched for genes overlapping candidate CNVRs under thermal adaptation selection.

Term	ID	P_Value	Matched_Genes
integral component of plasma membrane	GO:0005887	8.76596E-08	AQP5, PTPRS, TMEM206, ADCY2, PLSCR1, TSPAN10, AQP9, ADGRL4, ADGRD1, SLC16A14, TENM1, GRM8, CHRNA1, SGMS1, AQP2, TSPAN3, TRPV6, UGT1A1, ADGRB3
water channel activity	GO:0015250	5.90702E-05	AQP5, AQP2, AQP9
water transport	GO:0006833	5.90702E-05	AQP5, AQP2, AQP9
endoplasmic reticulum	GO:0005783	0.000235954	FAIM2, AQP5, PLOD2, TMTC3, NENF, KL, TENM1, INSIG2, SACM1L, SMPD2, SERAC1, CDKAL1
plasma membrane	GO:0005886	0.000345771	AOC1, ANTXR1, TENM1, PI4K2B, CNTN5, GPRC5C, WDR1, GPR142, TULP1, SLC34A2, TTYH2, SLC6A12, LIMD1, EPB41L3, AQP5, AQP2, RIMBP2, SCN8A, PIK3C2G, PTPRS, PLSCR1, STX2, PTEN
nucleus	GO:0005634	0.000422579	CLOCK, CELF2, ERCC6, EIF3E, SPRY2, TENM1, RBMS1, TEAD3, ARNTL2, MIPOL1, HSBP1, ZBTB14, RAN, USP40, RGS4, ZBTB38, IRX5, LIMD1, SOX5, PHACTR1, DIAPH3, FIGNL2, DRGX,

			HSPA4L, IGFBP3, SEPSECS, ZNF518B, UBE2V2, PTEN, ZNF407, ATF2
cell migration	GO:0016477	0.000619331	LAMA4, PEAK1, PTEN, PIK3C2G, ITGB5, LIMD1
histone acetylation	GO:0016573	0.000814122	ATF2, SGF29, CLOCK
blood vessel development	GO:0001568	0.00090369	LAMA4, ANTXR1, ALDH1A2
ceramide biosynthetic process	GO:0046513	0.00090369	DEGS2, SGMS1, SMPD2
phosphatidylinositol dephosphorylation	GO:0046856	0.001101015	PTEN, SACM1L, SYNJ2
glycerol transport	GO:0015793	0.00117637	AQP2, AQP9
stress fiber assembly	GO:0043149	0.00117637	ITGB5, PHACTR1
cellular response to copper ion	GO:0071280	0.00117637	AOC1, AQP2
GTPase activator activity	GO:0005096	0.001294964	ARAP2, SRGAP1, RGS18, RGS4, CHN1, ADGRB3
enzyme binding	GO:0019899	0.001552106	CTNNBL1, PTEN, CAT, UGT1A1, PLSCR1
sodium ion transmembrane transport	GO:0035725	0.00168872	SLC6A12, SLC34A2, SCN8A, SLC4A8
adenylate cyclase-activating G protein-coupled receptor signaling pathway	GO:0007189	0.001770985	ADGRL4, ADGRD1, ADCY2, ADGRB3
positive regulation of GTPase activity	GO:0043547	0.001794253	ARAP2, SRGAP1, RGS18, RGS4, CHN1, ADGRB3
negative regulation of axon regeneration	GO:0048681	0.001874287	PTEN, PTPRS
intraciliary retrograde transport	GO:0035721	0.001874287	IFT43, DYNC2H1
protein homotetramerization	GO:0051289	0.002153964	AQP5, AQP2, ALDH1A2
synaptic cleft	GO:0043083	0.002280903	LAMA4, ADGRB3
substrate	GO:0034446	0.003677683	LAMA4, ANTXR1,

adhesion-dependent cell spreading			PEAK1
calcium ion homeostasis	GO:0055074	0.003725226	TMTC2, KL
cardiac muscle tissue development	GO:0048738	0.004279814	PTEN, ALDH1A2
negative regulation of angiogenesis	GO:0016525	0.004381958	SPRY2, ATF2, ADGRB3
protein O-linked mannosylation	GO:0035269	0.004870148	TMTC3, TMTC2
neural crest cell development	GO:0014032	0.004870148	EDNRA, ALDH1A2
temperature homeostasis	GO:0001659	0.004870148	NMU, FTO
mannosyltransferase activity	GO:0000030	0.004870148	TMTC3, TMTC2
tRNA binding	GO:0000049	0.00516263	FARS2, XPOT, SEPSECS
inositol phosphate dephosphorylation	GO:0046855	0.005495739	PTEN, SYNJ2
integral component of membrane	GO:0016021	0.00590313	STX2, ANTXR1, CDH19, KCNG4, CSMD1, EDNRA, SLC4A8, GPRC5C, SLC25A29, GPR142, SLC16A14, SLC34A2, SLC6A12, AQP5, AQP2, SLC44A5, SLC13A4, MGAT2, TMEM184C, FAIM2, DEGS2, ROBO2, C11orf24, KL, TMTC3, TMTC2, HACL1, CDKAL1
spinal cord motor neuron differentiation	GO:0021522	0.006156104	DYNC2H1, ALDH1A2
neuronal action potential	GO:0019228	0.006156104	SCN8A, CHRNA1
extracellular exosome	GO:0070062	0.00663937	AOC1, AQP2, GPRC5C
neuromuscular synaptic transmission	GO:0007274	0.006850762	CHRNA1, RIMBP2
triglyceride metabolic	GO:0006641	0.006850762	INSIG2, CAT

process			
SH3 domain binding	GO:0017124	0.006961707	WIPF1, PLSCR1, SYNJ2
response to hypoxia	GO:0001666	0.007293192	PLOD2, EDNRA, LIMD1
cell motility	GO:0048870	0.00757924	PTEN, PHACTR1
endodermal cell differentiation	GO:0035987	0.00757924	ITGB5, HSBP1
adipose tissue development	GO:0060612	0.008341067	FTO, ATF2
protein localization to cilium	GO:0061512	0.009135778	TULP1, DYNC2H1
transmembrane transport	GO:0055085	0.009354748	SLC16A14, AQP5, AQP2, SLC44A5, SLC6A12
cytoplasm	GO:0005737	0.009759602	FARS2, SRGAP1, CELF2, CAT, FKBP5, TENM1, RBMS1, ARNTL2, ALDH1A2, HSBP1, RAN, RGS4, KCNIP1, ZCCHC4, AQP5, XPOT, WDR1, DIAPH3, AAK1, PIK3C2G, SEPSECS, FTO, ADCY2, RGS18, PTEN
coronary vasculature development	GO:0060976	0.009962913	DYNC2H1, ZBTB14
DNA damage checkpoint	GO:0000077	0.010822015	ERCC6, CLOCK
actomyosin structure organization	GO:0031032	0.011712633	EPB41L3, PHACTR1
forebrain development	GO:0030900	0.013586632	DYNC2H1, ALDH1A2
negative regulation of transcription, DNA-templated	GO:0045892	0.013841183	CLOCK, ZBTB38, LIMD1, ZBTB14, SOX5
phosphorylation	GO:0016310	0.01388329	LIMD1, PAPSS2, SGMS1
chloride transmembrane transport	GO:1902476	0.014377664	TTYH2, SLC4A8, TMEM206

phosphatidylinositol 3-kinase signaling	GO:0014065	0.014569133	PTEN, PIK3C2G
histone acetyltransferase activity	GO:0004402	0.014569133	ATF2, CLOCK
actin binding	GO:0003779	0.015290172	WIPF1, PHACTR1, EPB41L3, DIAPH3
cell surface receptor signaling pathway	GO:0007166	0.015396113	ADGRL4, ADGRD1, ADGRB3
photoreceptor cell maintenance	GO:0045494	0.015581388	TULP1, ERCC6
DNA-binding transcription factor activity	GO:0003700	0.015703181	CLOCK, ZBTB38, ARNTL2, ATF2, ZBTB14
regulation of cell growth	GO:0001558	0.016622968	IGFBP3, EPB41L3
cation channel activity	GO:0005261	0.016622968	SCN8A, UNC80
apical plasma membrane	GO:0016324	0.016742222	SLC34A2, AQP5, AQP2, PTEN
regulation of transcription, DNA-templated	GO:0006355	0.018642715	ZBTB38, LIMD1, CLOCK, ZNF518B, ZNF407
magnesium ion binding	GO:0000287	0.020740926	HACL1, RAN, STK38L, PGM1
cytosol	GO:0005829	0.021110972	CLOCK, EIF3E, SPRY2, RBMS1, DNAAF2, HSBP1, GPR142, ZBTB14, RAN, TULP1, USP40, CTNNBL1, KLHDC1, PHACTR1, XPOT, HSPA4L, PGM1, PTPRS, PLSCR1, PTEN, PLEKHA8, STK38L
presynaptic membrane	GO:0042734	0.022256007	STX2, SLC4A8
transport	GO:0006810	0.022256007	SLC6A12, TMEM184C
negative regulation of protein phosphorylation	GO:0001933	0.023464756	IGFBP3, PTEN
G protein-coupled receptor activity	GO:0004930	0.024804384	ADGRL4, ADGRD1, GPR142, GPRC5C,

			ADGRB3
actin filament binding	GO:0051015	0.025280475	ROBO2, ANTXR1, TULP1, WDR1
transcription regulator complex	GO:0005667	0.025764756	TEAD3, LIMD1, ARNTL2, CLOCK
positive regulation of translation	GO:0045727	0.027248045	EIF3E, ZCCHC4
signal transduction	GO:0007165	0.028054527	NMU, ARAP2, NENF, SRGAP1, TENM1, CHRNA1
protein homodimerization activity	GO:0042803	0.028364225	AOC1, PTPRS, MGAT2, CAT, ZBTB38, TENM1, UGT1A1
E-box binding	GO:0070888	0.028560182	ARNTL2, CLOCK
negative regulation of ERK1 and ERK2 cascade	GO:0070373	0.028560182	SPRY2, PTEN
cell-cell junction	GO:0005911	0.030738519	EPB41L3, LIMD1, WDR1
cell fate commitment	GO:0045165	0.031258706	SPRY2, SOX5
inner ear morphogenesis	GO:0042472	0.031258706	SPRY2, INSIG2
actin cytoskeleton reorganization	GO:0031532	0.031258706	ANTXR1, PHACTR1
cell surface	GO:0009986	0.033035679	BMP10, ANTXR1, ROBO2, ITGB5, CHRNA1
UV protection	GO:0009650	0.038883461	CAT
intraciliary transport particle A	GO:0030991	0.038883461	IFT43
phospholipid scramblase activity	GO:0017128	0.038883461	PLSCR1
negative regulation of collateral sprouting	GO:0048671	0.038883461	PTPRS
tRNA processing	GO:0008033	0.038883461	FARS2
type B pancreatic cell proliferation	GO:0044342	0.038883461	IGFBP3
activation of JNKK activity	GO:0007256	0.038883461	ERCC6
AP-2 adaptor complex binding	GO:0035612	0.038883461	AAK1
regulation of axon regeneration	GO:0048679	0.038883461	PTEN

oxysterol binding	GO:0008142	0.038883461	INSIG2
acetylcholine binding	GO:0042166	0.038883461	CHRNA1
urea transmembrane transport	GO:0071918	0.038883461	AQP9
cellular response to electrical stimulus	GO:0071257	0.038883461	PTEN
bud elongation involved in lung branching	GO:0060449	0.038883461	SPRY2
maternal behavior	GO:0042711	0.038883461	PTEN
regulation of feeding behavior	GO:0060259	0.038883461	NMU
DNA dealkylation involved in DNA repair	GO:0006307	0.038883461	FTO
neutrophil migration	GO:1990266	0.038883461	WDR1
sodium ion binding	GO:0031402	0.038883461	SLC34A2
negative regulation of smooth muscle cell migration	GO:0014912	0.038883461	IGFBP3
sphingomyelin biosynthetic process	GO:0006686	0.038883461	SGMS1
eye photoreceptor cell development	GO:0042462	0.038883461	TULP1
regulation of smooth muscle contraction	GO:0006940	0.038883461	NMU
presynaptic membrane assembly	GO:0097105	0.038883461	PTEN
telethonin binding	GO:0031433	0.038883461	BMP10
central nervous system neuron axonogenesis	GO:0021955	0.038883461	PTEN
response to interferon-beta	GO:0035456	0.038883461	PLSCR1
NSL complex	GO:0044545	0.038883461	KANSL1L
cranial suture morphogenesis	GO:0060363	0.038883461	INSIG2
regulation of glucose transmembrane transport	GO:0010827	0.038883461	EDNRA
selenocysteine incorporation	GO:0001514	0.038883461	SEPSECS
urea transmembrane	GO:0015204	0.038883461	AQP9

transporter activity			
ribosomal small subunit export from nucleus	GO:0000056	0.038883461	RAN
negative regulation of glucocorticoid receptor signaling pathway	GO:2000323	0.038883461	CLOCK
KICSTOR complex	GO:0140007	0.038883461	C1H12ORF66
acid phosphatase activity	GO:0003993	0.038883461	MINPP1
double-strand break repair via classical nonhomologous end joining	GO:0097680	0.038883461	ERCC6
multicellular organismal response to stress	GO:0033555	0.038883461	PTEN
protein geranylgeranylation	GO:0018344	0.038883461	RABGGTB
heart valve development	GO:0003170	0.038883461	ZBTB14
regulation of protein stability	GO:0031647	0.03992139	PTEN, USP40
phosphatidylinositol phosphorylation	GO:0046854	0.03992139	PI4K2B, PIK3C2G
neuron projection	GO:0043005	0.041613987	SLC6A12, TENM1, CHRNA1, PTEN
PDZ domain binding	GO:0030165	0.042988307	PTEN, SYNJ2
negative regulation of apoptotic process	GO:0043066	0.04357689	PTEN, SPRY2, SGMS1, CAT
voltage-gated potassium channel complex	GO:0008076	0.044553806	KCNG4, KCNIP1
regulation of clathrin-dependent endocytosis	GO:2000369	0.045216353	AAK1
heparan sulfate proteoglycan binding	GO:0043395	0.045216353	PTPRS
gamma-aminobutyric acid transport	GO:0015812	0.045216353	SLC6A12
sphingomyelin phosphodiesterase activity	GO:0004767	0.045216353	SMPD2

phosphate ion binding	GO:0042301	0.045216353	SLC34A2
aging	GO:0007568	0.045216353	KL
cilium-dependent cell motility	GO:0060285	0.045216353	DNAAF2
insulin-like growth factor II binding	GO:0031995	0.045216353	IGFBP3
negative regulation of cell size	GO:0045792	0.045216353	PTEN
apical junction assembly	GO:0043297	0.045216353	WDR1
negative regulation of vascular endothelial growth factor signaling pathway	GO:1900747	0.045216353	SPRY2
sodium:bicarbonate symporter activity	GO:0008510	0.045216353	SLC4A8
chondroitin sulfate binding	GO:0035374	0.045216353	PTPRS
ceramide transport	GO:0035627	0.045216353	PLEKHA8
peptidyl-lysine hydroxylation	GO:0017185	0.045216353	PLOD2
thiamine pyrophosphate binding	GO:0030976	0.045216353	HACL1
3',5'-cyclic-GMP phosphodiesterase activity	GO:0047555	0.045216353	PDE6G
phospholipase A1 activity	GO:0008970	0.045216353	PLA2G15
artery smooth muscle contraction	GO:0014824	0.045216353	EDNRA
positive regulation of actin filament depolymerization	GO:0030836	0.045216353	WDR1
negative regulation of cell aging	GO:0090344	0.045216353	PTEN
response to osmotic stress	GO:0006970	0.045216353	ATF2
photoreceptor outer segment membrane	GO:0042622	0.045216353	PDE6G
error-prone translesion synthesis	GO:0042276	0.045216353	POLE2
positive regulation of insulin-like growth	GO:0043568	0.045216353	IGFBP3

factor receptor signaling pathway			
gene silencing by miRNA	GO:0035195	0.045216353	LIMD1
ventricular cardiac muscle cell development	GO:0055015	0.045216353	BMP10
dorsal spinal cord development	GO:0021516	0.045216353	DRGX
cellular phosphate ion homeostasis	GO:0030643	0.045216353	SLC34A2
phagocytosis, recognition	GO:0006910	0.045216353	TULP1
adenylate cyclase-inhibiting G protein-coupled glutamate receptor signaling pathway	GO:0007196	0.045216353	GRM8
focal adhesion	GO:0005925	0.045998788	LIMD1, ITGB5, PEAK1
positive regulation of DNA-binding transcription factor activity	GO:0051091	0.046140207	PTEN, ATF2
neuromuscular junction	GO:0031594	0.046140207	LAMA4, CHRNA1
positive regulation of MAPK cascade	GO:0043410	0.047747171	IGFBP3, NENF
nucleoplasm	GO:0005654	0.047937155	PLA2G15, PTEN, FKBP5, XPOT, ZBTB14, HACL1, NENF, PLSCR1, CLOCK, ZBTB38, RAN, CTNNBL1, LIMD1, ARNTL2, PLEKHA8, ATF2
