# Supplementary material for: Genome-Wide Analysis Reveals Copy Number Variation-Mediated Adaptive Evolution in Chickens Under Diverse Environmental and Domestication Pressures
Source: Animals (Basel). 2026 Oct 29;16(21):3424. doi: 10.3390/ani16213424 (PMC13762271; doi:10.3390/ani16213424)
Supplement: Supplementary file 1 [file animals-16-03424-s001.zip › Table S6.pdf]

Table S6. KEGG pathways significantly enriched for genes overlapping candidate CNVRs under thermal adaptation selection.

Term	ID	P_Value	Matched_Genes
Inositol phosphate metabolism	gga00562	1.19746E-05	PTEN, PI4K2B, SACM1L, SYNJ2, PIK3C2G, MINPP1
Metabolic pathways	gga01100	0.000398344	AOC1, DEGS2, PAPSS2, ADCY2, PTEN, SMPD2, PGM1, PDE6G, KL, CAT, MGAT2, PIK3C2G, SACM1L, SGMS1, SYNJ2, PI4K2B, MINPP1, UGT1A1, ALDH1A2, SEPSECS
Phosphatidylinositol signaling system	gga04070	0.000439244	PTEN, PI4K2B, PIK3C2G, SACM1L, SYNJ2
Aminoacyl-tRNA biosynthesis	gga00970	0.00345949	FARS2, LARS2, SEPSECS
Sphingolipid metabolism	gga00600	0.003904097	DEGS2, SGMS1, SMPD2
Selenocompound metabolism	gga00450	0.005495739	PAPSS2, SEPSECS
Pentose and glucuronate interconversions	gga00040	0.009135778	UGT1A1, KL
Purine metabolism	gga00230	0.010564524	PAPSS2, PDE6G, ADCY2, PGM1
Nucleotide excision repair	gga03420	0.028560182	POLE2, ERCC6
Retinol metabolism	gga00830	0.029897195	UGT1A1, ALDH1A2
Tryptophan metabolism	gga00380	0.029897195	AOC1, CAT
