# Supplementary material for: Genome-Wide Analysis Reveals Copy Number Variation-Mediated Adaptive Evolution in Chickens Under Diverse Environmental and Domestication Pressures
Source: Animals (Basel). 2026 Oct 29;16(21):3424. doi: 10.3390/ani16213424 (PMC13762271; doi:10.3390/ani16213424)
Supplement: Supplementary file 1 [file animals-16-03424-s001.zip › Table S5.pdf]

Table S5. Candidate CNVRs and annotated genes under positive selection in the thermal adaptation group.

CHROM	BIN_START	BIN_END	Gene_Name
1	119940001	119980000	MAP7D2
1	119960001	120000000	MAP7D2
1	152600001	152640000	SPRY2
1	152620001	152660000	SPRY2
1	162340001	162380000	DIAPH3
1	174100001	174140000	KL
1	182140001	182180000	DYNC2H1
1	182160001	182200000	DYNC2H1
1	183500001	183540000	CNTN5
1	183520001	183560000	CNTN5
1	189720001	189760000	DLG2
1	21060001	21100000	ZNF800
1	21060001	21100000	GRM8
1	21080001	21120000	GRM8
1	21180001	21220000	GRM8
1	33700001	33740000	SRGAP1
1	33700001	33740000	C1H12ORF66
1	33700001	33740000	RPL18A
1	33700001	33740000	XPOT
1	33720001	33760000	C1H12ORF66
1	33720001	33760000	RPL18A
1	33720001	33760000	XPOT
1	41140001	41180000	TMTC2
1	42860001	42900000	CEP290
1	42880001	42920000	CEP290
1	42880001	42920000	TMTC3
1	58420001	58460000	SLC13A4
1	58420001	58460000	C7orf73
1	58440001	58480000	C7orf73
1	60200001	60240000	SLC6A12
1	6160001	6200000	CELF2
1	6180001	6220000	CELF2
1	64100001	64140000	PIK3C2G
1	64120001	64160000	PIK3C2G
1	65760001	65800000	SOX5
1	65760001	65800000	gene:ENSGALG00010014391
1	65780001	65820000	SOX5
1	65780001	65820000	gene:ENSGALG00010014391
1	68200001	68240000	STK38L

1	68200001	68240000	ARNTL2
1	68220001	68260000	ARNTL2
1	77840001	77880000	gene:ENSGALG00010021710
1	77840001	77880000	gene:ENSGALG00010021712
1	77840001	77880000	TRPV6
1	77860001	77900000	TRPV6
1	83700001	83740000	CD200R1A
1	97660001	97700000	ROBO2
2	107340001	107380000	gene:ENSGALG00010005395
2	107740001	107780000	UBE2V2
2	107740001	107780000	gene:ENSGALG00010005479
2	107760001	107800000	gene:ENSGALG00010005479
2	111780001	111820000	NSMAF
2	111800001	111840000	NSMAF
2	131220001	131260000	EIF3E
2	131220001	131260000	EMC2
2	131240001	131280000	EMC2
2	33860001	33900000	PLEKHA8
2	33860001	33900000	COLQ
2	33860001	33900000	HACL1
2	33880001	33920000	COLQ
2	33880001	33920000	HACL1
2	400001	440000	AOC1
2	400001	440000	gene:ENSGALG00010027194
2	400001	440000	gene:ENSGALG00010027196
2	400001	440000	gene:ENSGALG00010027197
2	400001	440000	gene:ENSGALG00010027210
2	400001	440000	gene:ENSGALG00010027215
2	400001	440000	gene:ENSGALG00010027219
2	42760001	42800000	SACM1L
2	42760001	42800000	LIMD1
2	42780001	42820000	LIMD1
2	42780001	42820000	LARS2
2	50400001	50440000	SUGCT
2	54620001	54660000	IGFBP3
2	58880001	58920000	CDKAL1
2	58900001	58940000	CDKAL1
2	62220001	62260000	PHACTR1
2	62240001	62280000	PHACTR1
2	65060001	65100000	FARS2
2	78900001	78940000	ADCY2
2	78920001	78960000	ADCY2
2	81440001	81480000	gene:ENSGALG00010007815

2	81440001	81480000	gene:ENSGALG00010007826
2	91220001	91260000	ZNF407
2	91240001	91280000	ZNF407
2	94800001	94840000	CDH19
2	94820001	94860000	CDH19
2	99820001	99860000	EPB41L3
2	99840001	99880000	ZBTB14
3	21580001	21620000	TMEM206
3	21580001	21620000	NENF
3	21600001	21640000	TMEM206
3	21600001	21640000	NENF
3	51500001	51540000	SYNJ2
3	51500001	51540000	SERAC1
3	65400001	65440000	LAMA4
3	82660001	82700000	COL19A1
3	82680001	82720000	COL19A1
3	83220001	83260000	ADGRB3
3	83240001	83280000	ADGRB3
3	89340001	89380000	CSMD1
3	98760001	98800000	gene:ENSGALG00010003639
3	98780001	98820000	gene:ENSGALG00010003639
4	15040001	15080000	TENM1
4	15060001	15100000	TENM1
4	15060001	15100000	gene:ENSGALG00010011666
4	31480001	31520000	EDNRA
4	31500001	31540000	EDNRA
4	31500001	31540000	TMEM184C
4	33820001	33860000	INTU
4	33840001	33880000	INTU
4	33840001	33880000	HSPA4L
4	64540001	64580000	NMU
4	64540001	64580000	PDCL2
4	64560001	64600000	NMU
4	64560001	64600000	PDCL2
4	64560001	64600000	CLOCK
4	69680001	69720000	DTHD1
4	69680001	69720000	ARAP2
4	69700001	69740000	DTHD1
4	69700001	69740000	ARAP2
4	73000001	73040000	SLC34A2
4	73000001	73040000	ANAPC4
4	73000001	73040000	ZCCHC4
4	73000001	73040000	PI4K2B

4	73020001	73060000	ANAPC4
4	73020001	73060000	ZCCHC4
4	73020001	73060000	PI4K2B
4	73020001	73060000	SEPSECS
4	77800001	77840000	ZNF518B
4	77800001	77840000	WDR1
4	77820001	77860000	WDR1
5	15840001	15880000	C11orf24
5	15860001	15900000	C11orf24
5	18200001	18240000	ABTB2
5	18200001	18240000	CAT
5	36580001	36620000	MIPOL1
5	38000001	38040000	IFT43
5	38020001	38060000	IFT43
5	48000001	48040000	DEGS2
5	48000001	48040000	YY2
5	48020001	48060000	YY2
5	48020001	48060000	SLC25A47
5	48020001	48060000	SLC25A29
5	57360001	57400000	MGAT2
5	57360001	57400000	DNAAF2
5	57360001	57400000	POLE2
5	57360001	57400000	KLHDC1
5	57380001	57420000	POLE2
5	57380001	57420000	KLHDC1
5	8500001	8540000	IRAG1
5	8520001	8560000	IRAG1
6	10240001	10280000	PTEN
6	10360001	10400000	PAPSS2
6	10360001	10400000	MINPP1
6	10360001	10400000	SGMS1
6	10380001	10420000	MINPP1
6	10380001	10420000	SGMS1
6	19440001	19480000	C6H10ORF71
6	19440001	19480000	DRGX
6	19460001	19500000	DRGX
6	19460001	19500000	gene:ENSGALG00010020021
6	19460001	19500000	ERCC6
7	16240001	16280000	ATP5G3
7	16260001	16300000	ATP5G3
7	16260001	16300000	ATF2
7	16380001	16420000	CHN1
7	16400001	16440000	CHN1

7	16400001	16440000	CHRNA1
7	16400001	16440000	WIPF1
7	21320001	21360000	RBMS1
7	21340001	21380000	RBMS1
7	2720001	2760000	UNC80
7	2740001	2780000	UNC80
7	2740001	2780000	gene:ENSGALG00010011374
7	2740001	2780000	KANSL1L
7	27500001	27540000	ITGB5
7	27500001	27540000	gene:ENSGALG00010020087
7	27520001	27560000	ITGB5
7	27520001	27560000	gene:ENSGALG00010020087
7	28460001	28500000	INSIG2
7	28460001	28500000	CCDC93
7	28480001	28520000	CCDC93
7	5880001	5920000	UGT1A1
7	5900001	5940000	UGT1A1
7	5900001	5940000	USP40
8	18280001	18320000	ADGRL4
8	18280001	18320000	VTG1
8	18300001	18340000	VTG1
8	27540001	27580000	EFCAB7
8	27540001	27580000	PGM1
8	29440001	29480000	SLC44A5
8	29440001	29480000	RABGGTB
8	29460001	29500000	SLC44A5
8	29460001	29500000	RABGGTB
8	29460001	29500000	SGF29
8	3660001	3700000	RGS4
8	3660001	3700000	RGS18
8	3660001	3700000	RGS5
8	3660001	3700000	gene:ENSGALG00010012851
9	11200001	11240000	PLOD2
9	11200001	11240000	PLSCR1
9	11220001	11260000	PLSCR1
9	9820001	9860000	ZBTB38
9	9820001	9860000	SLC16A14
9	9840001	9880000	ZBTB38
10	10660001	10700000	SPATA5L1
10	10660001	10700000	C10H15ORF48
10	13740001	13780000	AGBL1
10	13760001	13800000	AGBL1
10	3100001	3140000	PEAK1

10	3100001	3140000	TSPAN3
10	3120001	3160000	TSPAN3
10	7080001	7120000	AQP9
10	7100001	7140000	AQP9
10	7100001	7140000	ALDH1A2
11	1	40000	gene:ENSGALG00010022879
11	16240001	16280000	HSBP1
11	16260001	16300000	HSBP1
11	16280001	16320000	KCNG4
11	20001	60000	gene:ENSGALG00010022879
11	20001	60000	PLA2G15
11	3800001	3840000	IRX5
11	4360001	4400000	FTO
11	4380001	4420000	FTO
13	3260001	3300000	KCNIP1
13	3280001	3320000	KCNIP1
15	3180001	3220000	ADGRD1
15	3180001	3220000	STX2
15	3180001	3220000	RAN
15	3200001	3240000	ADGRD1
15	3200001	3240000	STX2
15	3200001	3240000	RAN
15	3200001	3240000	RIMBP2
18	9740001	9780000	TTYH2
18	9740001	9780000	DNAI2
18	9740001	9780000	KIF19
18	9740001	9780000	BTBD17
18	9740001	9780000	GPR142
18	9740001	9780000	GPRC5C
18	9740001	9780000	OXLD1
18	9740001	9780000	gene:ENSGALG00010029508
18	9740001	9780000	PDE6G
18	9740001	9780000	TSPAN10
20	10780001	10820000	CTNNBL1
20	10800001	10840000	CTNNBL1
20	10800001	10840000	WFDC2
20	10800001	10840000	gene:ENSGALG00010015828
20	10800001	10840000	gene:ENSGALG00010015836
20	10800001	10840000	gene:ENSGALG00010015842
20	10800001	10840000	gene:ENSGALG00010015847
20	10800001	10840000	gene:ENSGALG00010015854
20	10800001	10840000	gene:ENSGALG00010015859
21	20001	60000	KLHDC7A

21	40001	80000	KLHDC7A
21	60001	100000	KLHDC7A
21	80001	120000	KLHDC7A
22	280001	320000	ANTXR1
22	280001	320000	GKN1
22	280001	320000	GKN2
22	300001	340000	GKN1
22	300001	340000	GKN2
22	300001	340000	BMP10
22	60001	100000	AAK1
22	80001	120000	AAK1
26	80001	120000	SMPD2
26	80001	120000	TEAD3
26	80001	120000	TULP1
26	80001	120000	FKBP5
28	5000001	5040000	gene:ENSGALG00010027963
28	5000001	5040000	gene:ENSGALG00010027967
28	5020001	5060000	PTPRS
34	2160001	2200000	FAIM2
34	2160001	2200000	AQP2
34	2160001	2200000	gene:ENSGALG00010025602
34	2160001	2200000	AQP5
34	2160001	2200000	gene:ENSGALG00010025626
34	2160001	2200000	gene:ENSGALG00010025482
34	2180001	2220000	AQP5
34	2180001	2220000	gene:ENSGALG00010025626
34	2180001	2220000	gene:ENSGALG00010025482
34	2180001	2220000	SLC4A8
34	220001	260000	FIGNL2
34	220001	260000	SCN8A
34	220001	260000	gene:ENSGALG00010022277
