# Supplementary material for: Genome-Wide Analysis Reveals Copy Number Variation-Mediated Adaptive Evolution in Chickens Under Diverse Environmental and Domestication Pressures
Source: Animals (Basel). 2026 Oct 29;16(21):3424. doi: 10.3390/ani16213424 (PMC13762271; doi:10.3390/ani16213424)
Supplement: Supplementary file 1 [file animals-16-03424-s001.zip › Table S4.pdf]

Table S4. Population grouping for the altitude adaptation analysis.

Target group	control group
Lijiang Chicken	Yunyang Large Chicken
Nyingchi Chicken	Beijing You Chicken
Tibetan Black Chicken	Xianju Chicken
Tibetan chicken	Rhode Island Red chicken
Shannan Chicken	Liyang chicken
Nixi chicken	Baier Yellow Chicken
Chahua Chicken	Silkie
Wumeng Black-bone Chicken	Luxi Gamefowl
Lhasa White Chicken	Leghorn
Weining chicken	Huiyang Bearded Chicken
Gongbo'gyamda Chicken	Jining Bairi Chicken
	White Leghorn
	Wenchang Chicken
	Xinghua Chicken
	White Recessive Rock Chicken
	Sichuan low-altitude chicken
