# Supplementary material for: Genome-Wide Analysis Reveals Copy Number Variation-Mediated Adaptive Evolution in Chickens Under Diverse Environmental and Domestication Pressures
Source: Animals (Basel). 2026 Oct 29;16(21):3424. doi: 10.3390/ani16213424 (PMC13762271; doi:10.3390/ani16213424)
Supplement: Supplementary file 1 [file animals-16-03424-s001.zip › Table S3.pdf]

Table S3. Population grouping for the domestication analysis.

Target group	control group
Xianju Chicken	Red Junglefowl
Jining Bairi Chicken	Hongyuan Podiceps cristatus subspecies
Lindian chicken	Gallus gallus murghi
Hetian Chicken	Gallus gallus gallus
Lijiang Chicken	Gallus gallus spadiceus
Weining chicken	
Qiandongnan Small Fragrant Chicken	
Tibetan chicken	
