# Supplementary material for: Genome-Wide Analysis Reveals Copy Number Variation-Mediated Adaptive Evolution in Chickens Under Diverse Environmental and Domestication Pressures
Source: Animals (Basel). 2026 Oct 29;16(21):3424. doi: 10.3390/ani16213424 (PMC13762271; doi:10.3390/ani16213424)
Supplement: Supplementary file 1 [file animals-16-03424-s001.zip › Table S2.pdf]

Table S2. Population grouping for the thermal adaptation analysis.

Target group	control group
Huiyang Bearded Chicken	Korean native chicken
Xinghua Chicken	Norwegian Jaerhon
Heike Duji	Orloff chicken
Local chicken breeds of Ethiopia	Jining Bairi Chicken
Qiandongnan Mini Fragrant Chicken	Lindian chicken
Silkie	Beijing You Chicken
Fayoumi chicken	Luxi Fighting Chicken
Wenchang Chicken	
