# Supplementary material for: Genome-Wide Analysis Reveals Copy Number Variation-Mediated Adaptive Evolution in Chickens Under Diverse Environmental and Domestication Pressures
Source: Animals (Basel). 2026 Oct 29;16(21):3424. doi: 10.3390/ani16213424 (PMC13762271; doi:10.3390/ani16213424)
Supplement: Supplementary file 1 [file animals-16-03424-s001.zip › Table S1.pdf]

Table S1. Sample information and geographic origins of the chicken populations used in this study.

RUN	Breed	Area
DRR089959	Gallus varius1	Indonesia:Java
DRR089960	Gallus varius2	Indonesia:Java
DRR089961	Gallus varius3	Indonesia:Madura
DRR089962	Gallus varius4	Indonesia:Madura
DRR089963	Gallus varius5	Indonesia:Madura
DRR089964	Gallus varius6	Indonesia:Madura
DRR089965	Gallus varius7	Indonesia:Madura
DRR089966	Gallus varius8	Indonesia:Madura
DRR089967	Gallus varius9	Indonesia:Madura
DRR089968	Gallus gallus1	Indonesia:Java
DRR089969	Gallus gallus2	Indonesia:Java
DRR089970	Gallus gallus3	Indonesia:Java
DRR089971	Gallus gallus4	Indonesia:Sumatra
DRR089972	Gallus gallus5	Indonesia:Sumatra
DRR089973	Black Sumatra1	American
DRR089974	Black Sumatra2	American
DRR089975	Black Sumatra3	American
DRR089976	Black Sumatra4	American
DRR089977	Black Sumatra5	American
DRR089978	Black Sumatra6	American
DRR089979	Black Sumatra7	American
DRR089980	Black Sumatra8	American
DRR089981	Black Sumatra9	American
DRR089982	Black Sumatra10	American
DRR089983	Black Sumatra11	American
DRR089984	Black Sumatra12	American
DRR089985	Black Sumatra13	American
DRR089986	Black Sumatra14	American
DRR089987	Black Sumatra15	American
DRR089988	Kedu Hitam1	Indonesia:Java
DRR089989	Kedu Hitam2	Indonesia:Java
DRR089990	Kedu Hitam3	Indonesia:Java
DRR089991	Kedu Hitam4	Indonesia:Java
DRR089992	Kedu Hitam5	Indonesia:Java
DRR089993	Kedu Hitam6	Indonesia:Java
DRR089994	Kedu Hitam7	Indonesia:Java
DRR089995	Kedu Hitam8	Indonesia:Java
DRR089996	Kedu Hitam9	Indonesia:Java
DRR089997	Kedu Hitam10	Indonesia:Java
DRR089998	Black Java1	American

DRR089999	Black Java2	American
DRR090000	Black Java3	American
DRR090001	Black Java4	American
DRR090002	Black Java5	American
DRR090003	Black Java6	American
DRR090004	Black Java7	American
DRR090005	Black Java8	American
DRR090006	Black Java9	American
DRR090007	Black Java10	American
ERR4344204	Ethiopian indigenous chicken1	Ethiopia
ERR4344207	Ethiopian indigenous chicken2	Ethiopia
ERR4344217	Ethiopian indigenous chicken3	Ethiopia
ERR4345015	Ethiopian indigenous chicken4	Ethiopia
ERR4346501	Ethiopian indigenous chicken5	Ethiopia
ERR4346562	Ethiopian indigenous chicken6	Ethiopia
ERR4349139	Ethiopian indigenous chicken7	Ethiopia
ERR4349374	Ethiopian indigenous chicken8	Ethiopia
ERR4349376	Ethiopian indigenous chicken9	Ethiopia
ERR4350090	Ethiopian indigenous chicken10	Ethiopia
ERR4350101	Ethiopian indigenous chicken11	Ethiopia
ERR4350102	Ethiopian indigenous chicken12	Ethiopia
ERR4350108	Ethiopian indigenous chicken13	Ethiopia
ERR4350110	Ethiopian indigenous chicken14	Ethiopia
ERR4350116	Ethiopian indigenous chicken15	Ethiopia
ERR4350148	Ethiopian indigenous chicken16	Ethiopia
ERR4350697	Ethiopian indigenous chicken17	Ethiopia
ERR4351083	Ethiopian indigenous chicken18	Ethiopia
ERR4351085	Ethiopian indigenous chicken19	Ethiopia
ERR4351344	Ethiopian indigenous chicken20	Ethiopia
SRR12145401	Xinghua Chicken1	Fengkai County, Guangdong Province
SRR12145402	Xinghua Chicken2	Fengkai County, Guangdong Province
SRR12145403	Xinghua Chicken3	Fengkai County, Guangdong Province
SRR12145404	Xinghua Chicken4	Fengkai County, Guangdong Province
SRR12145405	Xinghua Chicken5	Fengkai County, Guangdong Province
SRR12145406	Xinghua Chicken6	Fengkai County, Guangdong Province
SRR12145407	Xinghua Chicken7	Fengkai County, Guangdong Province

SRR12145408	Xinghua Chicken8	Fengkai County, Guangdong Province
SRR12145409	Xinghua Chicken9	Fengkai County, Guangdong Province
SRR12145410	White Recessive Rock Chicken1	Foshan
SRR12145412	White Recessive Rock Chicken2	Foshan
SRR12145413	White Recessive Rock Chicken3	Foshan
SRR12145414	White Recessive Rock Chicken4	Foshan
SRR12145415	White Recessive Rock Chicken5	Foshan
SRR12145416	White Recessive Rock Chicken6	Foshan
SRR12145417	Turpan Wild Chicken1	Turpan City
SRR12145418	Turpan Wild Chicken2	Turpan City
SRR12145419	Turpan Wild Chicken3	Turpan City
SRR12145420	Turpan Wild Chicken4	Turpan City
SRR12145421	Turpan Wild Chicken5	Turpan City
SRR12145423	Turpan Wild Chicken6	Turpan City
SRR12145444	Bai'er Yellow Chicken1	Shangrao, Jiangxi
SRR12145433	Bai'er Yellow Chicken2	Shangrao, Jiangxi
SRR12145422	Bai'er Yellow Chicken3	Shangrao, Jiangxi
SRR12145411	Bai'er Yellow Chicken4	Shangrao, Jiangxi
SRR12145520	Bai'er Yellow Chicken5	Shangrao, Jiangxi
SRR12145509	Bai'er Yellow Chicken6	Shangrao, Jiangxi
SRR12145424	Silkie Chicken1	Taihe, Jiangxi
SRR12145425	Silkie Chicken2	Taihe, Jiangxi
SRR12145426	Silkie Chicken3	Taihe, Jiangxi
SRR12145427	Silkie Chicken4	Taihe, Jiangxi
SRR12145428	Silkie Chicken5	Taihe, Jiangxi
SRR12145429	Silkie Chicken6	Taihe, Jiangxi
SRR12145430	Silkie Chicken7	Taihe, Jiangxi
SRR12145431	Silkie Chicken8	Taihe, Jiangxi
SRR12145432	Silkie Chicken9	Taihe, Jiangxi
SRR12145434	Silkie Chicken10	Taihe, Jiangxi
SRR12145435	Silkie Chicken11	Taihe, Jiangxi
SRR12145442	Red Junglefowl1	Guangxi
SRR12145443	Red Junglefowl2	Guangxi
SRR12145445	Red Junglefowl3	Guangxi
SRR12145446	Red Junglefowl4	Guangxi
SRR12145447	Red Junglefowl5	Guangxi
SRR12145448	Rhode Island Red Chicken1	Beijing
SRR12145449	Rhode Island Red Chicken2	Beijing
SRR12145450	Rhode Island Red Chicken3	Beijing
SRR12145451	Rhode Island Red Chicken4	Beijing
SRR12145452	Rhode Island Red Chicken5	Beijing

SRR12145453	Rhode Island Red Chicken6	Beijing
SRR12145460	Liyang Chicken1	Liyang, Jiangsu
SRR12145459	Liyang Chicken2	Liyang, Jiangsu
SRR12145458	Liyang Chicken3	Liyang, Jiangsu
SRR12145457	Liyang Chicken4	Liyang, Jiangsu
SRR12145456	Liyang Chicken5	Liyang, Jiangsu
SRR12145454	Liyang Chicken6	Liyang, Jiangsu
SRR12145461	Luxi Game Chicken1	Heze, Shandong
SRR12145462	Luxi Game Chicken2	Heze, Shandong
SRR12145463	Luxi Game Chicken3	Heze, Shandong
SRR12145464	Luxi Game Chicken4	Heze, Shandong
SRR12145465	Luxi Game Chicken5	Heze, Shandong
SRR12145467	Luxi Game Chicken6	Heze, Shandong
SRR12145468	Luxi Game Chicken7	Heze, Shandong
SRR12145469	Luxi Game Chicken8	Heze, Shandong
SRR12145470	Luxi Game Chicken9	Heze, Shandong
SRR12145471	Luxi Game Chicken10	Heze, Shandong
SRR12145472	Leghorn1	Guangzhou
SRR12145473	Leghorn2	Guangzhou
SRR12145474	Leghorn3	Guangzhou
SRR12145475	Leghorn4	Guangzhou
SRR12145476	Leghorn5	Guangzhou
SRR12145478	Leghorn6	Guangzhou
SRR1744077	Leghorn7	South Korea
SRR1744079	Leghorn8	South Korea
SRR1744080	Leghorn9	South Korea
SRR12145479	Lindian chicken1	Lindian, Heilongjiang
SRR12145480	Lindian chicken2	Lindian, Heilongjiang
SRR12145481	Lindian chicken3	Lindian, Heilongjiang
SRR12145482	Lindian chicken4	Lindian, Heilongjiang
SRR12145485	Laos1	Laos
SRR12145486	Laos2	Laos
SRR12145487	Laos3	Laos
SRR12145455	Huiyang Bearded Chicken1	Dongjiang, Guangdong
SRR12145466	Huiyang Bearded Chicken2	Dongjiang, Guangdong
SRR12145477	Huiyang Bearded Chicken3	Dongjiang, Guangdong
SRR12145488	Huiyang Bearded Chicken4	Dongjiang, Guangdong
SRR12145521	Huiyang Bearded Chicken5	Dongjiang, Guangdong
SRR12145522	Huiyang Bearded Chicken6	Dongjiang, Guangdong
SRR12145491	Jining Bainian Chicken1	Jining, Shandong
SRR12145492	Jining Bainian Chicken2	Jining, Shandong
SRR12145493	Jining Bainian Chicken3	Jining, Shandong
SRR12145494	Jining Bainian Chicken4	Jining, Shandong

SRR12145495	Jining Bainian Chicken5	Jining, Shandong
SRR12145498	Jining Bainian Chicken6	Jining, Shandong
SRR12145496	Yunyang Large Chicken1	Shiyan, Hubei
SRR12145497	Yunyang Large Chicken2	Shiyan, Hubei
SRR12145499	Yunyang Large Chicken3	Shiyan, Hubei
SRR12145500	Yunyang Large Chicken4	Shiyan, Hubei
SRR12145501	Yunyang Large Chicken5	Shiyan, Hubei
SRR12145502	Yunyang Large Chicken6	Shiyan, Hubei
SRR12145503	Beijing You Chicken1	Beijing
SRR12145504	Beijing You Chicken2	Beijing
SRR12145505	Beijing You Chicken3	Beijing
SRR12145506	Beijing You Chicken4	Beijing
SRR12145507	Beijing You Chicken5	Beijing
SRR12145508	Beijing You Chicken6	Beijing
SRR12145510	Beijing You Chicken7	Beijing
SRR12145511	Beijing You Chicken8	Beijing
SRR12145512	Beijing You Chicken9	Beijing
SRR12145513	Beijing You Chicken10	Beijing
SRR12145514	Xianju Chicken1	Zhejiang
SRR12145515	Xianju Chicken2	Zhejiang
SRR12145516	Xianju Chicken3	Zhejiang
SRR12145517	Xianju Chicken4	Zhejiang
SRR12145518	Xianju Chicken5	Zhejiang
SRR12145519	Xianju Chicken6	Zhejiang
SRR1217491	tibetan chicken1	Xizang
SRR1217492	tibetan chicken2	Xizang
SRR1217493	tibetan chicken3	Xizang
SRR1217494	tibetan chicken4	Xizang
SRR1217495	tibetan chicken5	Xizang
SRR1217496	tibetan chicken6	Xizang
SRR1217497	tibetan chicken7	Xizang
SRR1217498	tibetan chicken8	Xizang
SRR1217499	tibetan chicken9	Xizang
SRR1217506	tibetan chicken10	Xizang
SRR1217507	tibetan chicken11	Xizang
SRR1217508	tibetan chicken12	Xizang
SRR1217555	tibetan chicken13	Xizang
SRR1217556	tibetan chicken14	Xizang
SRR1217557	tibetan chicken15	Xizang
SRR1217558	tibetan chicken16	Xizang
SRR1217559	tibetan chicken17	Xizang
SRR1217560	tibetan chicken18	Xizang
SRR1217561	tibetan chicken19	Xizang

SRR1217562	tibetan chicken20	Xizang
SRR1217563	tibetan chicken21	Xizang
SRR1217512	fighting chicken1	Yunnan
SRR1217514	fighting chicken2	Yunnan
SRR1217516	fighting chicken3	Yunnan
SRR1217518	fighting chicken4	Yunnan
SRR1217519	fighting chicken5	Yunnan
SRR1217520	fighting chicken6	Yunnan
SRR1217535	native chicken1	Yunnan
SRR1217536	native chicken2	Yunnan
SRR1217537	native chicken3	Yunnan
SRR1217538	native chicken4	Yunnan
SRR1217539	native chicken5	Yunnan
SRR1217540	native chicken6	Yunnan
SRR1217541	native chicken7	Yunnan
SRR1217542	native chicken8	Yunnan
SRR1217543	native chicken9	Yunnan
SRR1217544	native chicken10	Yunnan
SRR1217545	native chicken11	Yunnan
SRR1217546	native chicken12	Yunnan
SRR1217547	native chicken13	Yunnan
SRR1217548	native chicken14	Yunnan
SRR1217549	native chicken15	Yunnan
SRR1217550	native chicken16	Yunnan
SRR1217551	native chicken17	Yunnan
SRR1217552	native chicken18	Yunnan
SRR1217553	native chicken19	Yunnan
SRR1217554	commercial chicken	Yunnan
SRR12279032	Hetian Chicken1	Changting, Fujian
SRR12279033	Hetian Chicken2	Changting, Fujian
SRR12279034	Hetian Chicken3	Changting, Fujian
SRR12279035	Hetian Chicken4	Changting, Fujian
SRR12145489	Hetian Chicken5	Changting, Fujian
SRR12145490	Hetian Chicken6	Changting, Fujian
SRR12868129	Gallus gallus murghi1	India
SRR12868130	Gallus gallus murghi2	India
SRR12868131	Gallus gallus murghi3	India
SRR12868132	Gallus gallus gallus1	Thailand
SRR12868133	Gallus gallus gallus2	Thailand
SRR12868134	Gallus gallus gallus3	Thailand
SRR12868141	Gallus gallus gallus4	Thailand
SRR12868138	Gallus sonneratii1	India
SRR12868139	Gallus sonneratii2	India

SRR12868140	Gallus sonneratii3	France
SRR12868142	Gallus sonneratii4	France
SRR12868143	Gallus sonneratii5	Japan
SRR12868144	Gallus lafayetii1	France
SRR12868145	Gallus lafayetii2	France
SRR12868146	Gallus gallus spadiceus1	India
SRR12868147	Gallus gallus spadiceus2	India
SRR12868148	Gallus gallus spadiceus3	Viet Nam
SRR12868149	Gallus gallus spadiceus4	Viet Nam
SRR12868150	Gallus gallus spadiceus5	Thailand
SRR12868151	Gallus gallus spadiceus6	Thailand
SRR12868128	Gallus gallus spadiceus7	Thailand
SRR12868152	Gallus gallus bankiva1	France
SRR12868153	Gallus gallus bankiva2	France
SRR16992086	Chahua chicken1	Yunnan
SRR16992087	Chahua chicken2	Yunnan
SRR16992088	Chahua chicken3	Yunnan
SRR16992089	Chahua chicken4	Yunnan
SRR16992090	Chahua chicken5	Yunnan
SRR16992269	Chahua chicken6	Yunnan
SRR16992270	Chahua chicken7	Yunnan
SRR3145345	Chahua chicken8	Xizang
SRR16992092	Red jungle fowl1	Yunnan
SRR16992093	Red jungle fowl2	Yunnan
SRR16992094	Red jungle fowl3	Yunnan
SRR16992095	Red jungle fowl4	Yunnan
SRR16992097	Red jungle fowl5	Yunnan
SRR16992098	Red jungle fowl6	Yunnan
SRR16992099	Red jungle fowl7	Yunnan
SRR16992100	Red jungle fowl8	Yunnan
SRR16992101	Red jungle fowl9	Yunnan
SRR16992102	Red jungle fowl10	Yunnan
SRR1217524	Red jungle fowl11	Yunnan
SRR1217525	Red jungle fowl12	Yunnan
SRR1217526	Red jungle fowl13	Yunnan
SRR1217527	Red jungle fowl14	Yunnan
SRR1217528	Red jungle fowl15	Yunnan
SRR1217529	Red jungle fowl16	Yunnan
SRR1217530	Red jungle fowl17	Yunnan
SRR1217531	Red jungle fowl18	Vietnam
SRR1217532	Red jungle fowl19	Vietnam
SRR1217533	Red jungle fowl20	Yunnan
SRR1217534	Red jungle fowl21	Yunnan

SRR17916227	Red jungle fowl22	Guadeloupe
SRR17916229	Red jungle fowl23	Ivory Coast
SRR17916235	Red jungle fowl24	Japan
SRR17916232	Red jungle fowl25	Israel
SRR12868135	Red jungle fowl26	Cameroon
SRR12868136	Red jungle fowl27	Japan
SRR12868137	Red jungle fowl28	Taiwan
SRR17916238	Red jungle fowl29	Iceland
SRR17916239	Red jungle fowl30	Taiwan, China
SRR17916240	Red jungle fowl31	Netherlands
SRR17916241	Red jungle fowl32	Chile
SRR16992103	Nixi Chicken1	Nixi, Yunnan
SRR16992104	Nixi Chicken2	Nixi, Yunnan
SRR16992105	Nixi Chicken3	Nixi, Yunnan
SRR16992106	Nixi Chicken4	Nixi, Yunnan
SRR16992108	Nixi Chicken5	Nixi, Yunnan
SRR16992109	Nixi Chicken6	Nixi, Yunnan
SRR16992110	Nixi Chicken7	Nixi, Yunnan
SRR16992111	Nixi Chicken8	Nixi, Yunnan
SRR16992112	Nixi Chicken9	Nixi, Yunnan
SRR3145350	Nixi Chicken10	Nyingchi, Xizang
SRR16992115	shannan Chicken1	Nyingchi, Xizang
SRR16992116	Linzhi indigenous chicken1	Nyingchi, Xizang
SRR16992117	Linzhi indigenous chicken2	Nyingchi, Xizang
SRR16992119	Linzhi indigenous chicken3	Nyingchi, Xizang
SRR16992120	Linzhi indigenous chicken4	Nyingchi, Xizang
SRR16992121	Linzhi indigenous chicken5	Nyingchi, Xizang
SRR16992122	Linzhi indigenous chicken6	Nyingchi, Xizang
SRR16992123	Linzhi indigenous chicken7	Nyingchi, Xizang
SRR16992124	Linzhi indigenous chicken8	Nyingchi, Xizang
SRR16992126	Lijiang indigenous chicken1	Lijiang, Yunnan
SRR16992127	Lijiang indigenous chicken2	Lijiang, Yunnan
SRR16992128	Lijiang indigenous chicken3	Lijiang, Yunnan
SRR16992130	Lijiang indigenous chicken4	Lijiang, Yunnan
SRR16992131	Lijiang indigenous chicken5	Lijiang, Yunnan
SRR16992132	Lijiang indigenous chicken6	Lijiang, Yunnan
SRR16992133	Lijiang indigenous chicken7	Lijiang, Yunnan
SRR16992134	Lijiang indigenous chicken8	Lijiang, Yunnan
SRR16992135	Lijiang indigenous chicken9	Lijiang, Yunnan
SRR16992149	Gongbujiangda Chicken1	Xizang
SRR16992150	Gongbujiangda Chicken2	Xizang
SRR16992152	Gongbujiangda Chicken3	Xizang
SRR16992153	Gongbujiangda Chicken4	Xizang

SRR16992154	Gongbujiangda Chicken5	Xizang
SRR16992155	Gongbujiangda Chicken6	Xizang
SRR16992156	Gongbujiangda Chicken7	Xizang
SRR16992157	Gongbujiangda Chicken8	Xizang
SRR16992175	Zhenyuan Chicken1	Zhenyuan Chicken
SRR16992176	Zhenyuan Chicken2	Zhenyuan Chicken
SRR16992177	Zhenyuan Chicken3	Zhenyuan Chicken
SRR16992178	Zhenyuan Chicken4	Zhenyuan Chicken
SRR16992179	Zhenyuan Chicken5	Zhenyuan Chicken
SRR16992180	Zhenyuan Chicken6	Zhenyuan Chicken
SRR16992203	Zhenyuan Chicken7	Zhaotong Chicken
SRR16992204	Zhenyuan Chicken8	Zhaotong Chicken
SRR16992206	Zhenyuan Chicken9	Zhaotong Chicken
SRR16992207	Zhenyuan Chicken10	Zhaotong Chicken
SRR16992208	Zhenyuan Chicken11	Zhaotong Chicken
SRR16992209	Zhenyuan Chicken12	Zhaotong Chicken
SRR16992214	Shannan Chicken2	Xizang
SRR16992215	Shannan Chicken3	Xizang
SRR16992217	Shannan Chicken4	Xizang
SRR16992218	Shannan Chicken5	Xizang
SRR16992219	Shannan Chicken6	Xizang
SRR16992233	Yimen Chicken1	Yimen Chicken
SRR16992234	Yimen Chicken2	Yimen Chicken
SRR16992235	Yimen Chicken3	Yimen Chicken
SRR16992236	Yimen Chicken4	Yimen Chicken
SRR16992237	Yimen Chicken5	Yimen Chicken
SRR16992239	Yimen Chicken6	Yimen Chicken
SRR16992244	Wuliangshan Chicken1	Wuliangshan Chicken
SRR16992245	Wuliangshan Chicken2	Wuliangshan Chicken
SRR16992246	Wuliangshan Chicken3	Wuliangshan Chicken
SRR16992247	Wuliangshan Chicken4	Wuliangshan Chicken
SRR16992248	Wuliangshan Chicken5	Wuliangshan Chicken
SRR1744074	Gyeongbuk Araucana1	South Korea
SRR1744075	Gyeongbuk Araucana2	South Korea
SRR1744078	Gyeongbuk Araucana3	South Korea
SRR1744081	Korean native chicken1	South Korea
SRR1744082	Korean native chicken2	South Korea
SRR1744083	Korean native chicken3	South Korea
SRR1770445	Kauai feral chickens1	Hawaii, USA
SRR1772172	Kauai feral chickens2	Hawaii, USA
SRR1772178	Kauai feral chickens3	Hawaii, USA
SRR1772198	Kauai feral chickens4	Hawaii, USA
SRR1772199	Kauai feral chickens5	Hawaii, USA

SRR17916225	Miao indigenous chicken	Vietnam
SRR17916226	Gallic Golden Chicken	France
SRR17916228	Fayoumi Chicken	Egypt
SRR17916230	Jiujinhuang Chicken	England
SRR17916231	Indonesian Black Phoenix Chicken	Indonesia
SRR17916233	Red Villafranquina	Spain
SRR17916234	Orlov	Russia
SRR17916236	Marans	France
SRR17916237	Jaerhoens	Norway
SRR18363594	tibetan chicken22	Garze, Sichuan
SRR18363599	tibetan chicken23	Garze, Sichuan
SRR18363600	tibetan chicken24	Garze, Sichuan
SRR18363601	tibetan chicken25	Garze, Sichuan
SRR18363602	tibetan chicken26	Garze, Sichuan
SRR18363607	tibetan chicken27	Garze, Sichuan
SRR18363608	tibetan chicken28	Garze, Sichuan
SRR18363609	tibetan chicken29	Garze, Sichuan
SRR18363610	tibetan chicken30	Garze, Sichuan
SRR18363611	tibetan chicken31	Garze, Sichuan
SRR18363612	tibetan chicken32	Garze, Sichuan
SRR18363613	tibetan chicken33	Garze, Sichuan
SRR18363615	tibetan chicken34	Garze, Sichuan
SRR18363616	tibetan chicken35	Garze, Sichuan
SRR5149620	tibetan chicken36	Garze, Sichuan
SRR3145347	Lhasa White1	Lhasa, Xizang
SRR3145348	Lhasa White2	Lhasa, Xizang
SRR3145351	Lhasa White3	Beijing
SRR3145352	Lhasa White4	Beijing
SRR3145349	tibetan Linzhi37	Nyingchi, Xizang
SRR18363604	Wenchang chicken1	Wenchang, Hainan
SRR18363593	Wenchang chicken2	Wenchang, Hainan
SRR18363621	Wenchang chicken3	Wenchang, Hainan
SRR18363634	Wenchang chicken4	Wenchang, Hainan
SRR18363636	Wenchang chicken5	Wenchang, Hainan
SRR18363637	Wenchang chicken6	Wenchang, Hainan
SRR18363638	Wenchang chicken7	Wenchang, Hainan
SRR18363639	Wenchang chicken8	Wenchang, Hainan
SRR18363640	Wenchang chicken9	Wenchang, Hainan
SRR18363641	Wenchang chicken10	Wenchang, Hainan
SRR18363642	Wenchang chicken11	Wenchang, Hainan
SRR18363643	Wenchang chicken12	Wenchang, Hainan
SRR18363682	Green-shell laying hens with black feather	Yangzhou, Jiangsu
SRR18363630	Green-shell laying hens with hemp feather1	Yangzhou, Jiangsu

SRR18363631	Green-shell laying hens with hemp feather2	Yangzhou, Jiangsu
SRR18363632	Green-shell laying hens with hemp feather3	Yangzhou, Jiangsu
SRR18363633	Green-shell laying hens with hemp feather4	Yangzhou, Jiangsu
SRR18363635	Green-shell laying hens with hemp feather5	Yangzhou, Jiangsu
SRR18363683	Green-shell laying hens with hemp feather6	Yangzhou, Jiangsu
SRR18363684	Green-shell laying hens with hemp feather7	Yangzhou, Jiangsu
SRR18363685	Green-shell laying hens with hemp feather8	Yangzhou, Jiangsu
SRR18363686	Green-shell laying hens with hemp feather9	Yangzhou, Jiangsu
SRR18363688	Green-shell laying hens with hemp feather10	Yangzhou, Jiangsu
SRR18363689	Green-shell laying hens with hemp feather11	Yangzhou, Jiangsu
SRR18363690	Green-shell laying hens with hemp feather12	Yangzhou, Jiangsu
SRR18363691	Green-shell laying hens with hemp feather13	Yangzhou, Jiangsu
SRR18363692	Green-shell laying hens with hemp feather14	Yangzhou, Jiangsu
SRR18363705	Lindian chicken5	Lindian, Heilongjiang
SRR18363706	Lindian chicken6	Lindian, Heilongjiang
SRR18363707	Lindian chicken7	Lindian, Heilongjiang
SRR18363708	Lindian chicken8	Lindian, Heilongjiang
SRR18363709	Lindian chicken9	Lindian, Heilongjiang
SRR18363710	Lindian chicken10	Lindian, Heilongjiang
SRR18363712	Lindian chicken11	Lindian, Heilongjiang
SRR18363713	Lindian chicken12	Lindian, Heilongjiang
SRR18363714	Lindian chicken13	Lindian, Heilongjiang
SRR18363715	Lindian chicken14	Lindian, Heilongjiang
SRR18363716	Lindian chicken15	Lindian, Heilongjiang
SRR18363717	Lindian chicken16	Lindian, Heilongjiang
SRR18363718	Lindian chicken17	Lindian, Heilongjiang
SRR18363719	Lindian chicken18	Lindian, Heilongjiang
SRR18363720	Lindian chicken19	Lindian, Heilongjiang
SRR18363721	Lindian chicken20	Lindian, Heilongjiang
SRR1951793	White Leghorn1	Beijing
SRR1951796	White Leghorn2	Beijing
SRR1951798	White Leghorn3	Beijing
SRR1951801	White Leghorn4	Beijing
SRR1951806	White Leghorn5	Beijing
SRR1951821	White Leghorn6	Beijing
SRR2153337	Wellcome	Pirbright Institute
SRR2154196	Village chicken1	Horro,Jarso,Ethiopia
SRR2154198	Village chicken2	Horro,Jarso,Ethiopia
SRR2154199	Village chicken3	Horro,Jarso,Ethiopia
SRR2154200	Village chicken4	Horro,Jarso,Ethiopia
SRR2154201	Village chicken5	Horro,Jarso,Ethiopia
SRR2154203	Village chicken6	Horro,Jarso,Ethiopia

SRR2154224	Village chicken7	Horro,Jarso,Ethiopia
SRR2154237	Village chicken8	Horro,Jarso,Ethiopia
SRR2154242	Village chicken9	Horro,Jarso,Ethiopia
SRR2154250	Village chicken10	Horro,Jarso,Ethiopia
SRR23936536	Weining Chicken1	Weining, Guizhou
SRR23936542	Weining Chicken2	Weining, Guizhou
SRR23936553	Weining Chicken3	Weining, Guizhou
SRR23936593	Weining Chicken4	Weining, Guizhou
SRR23936604	Weining Chicken5	Weining, Guizhou
SRR23936612	Weining Chicken6	Weining, Guizhou
SRR23936623	Weining Chicken7	Weining, Guizhou
SRR23936640	Weining Chicken8	Weining, Guizhou
SRR23936651	Weining Chicken9	Weining, Guizhou
SRR23936537	Qiandongnan Xiaoxiang Chicken1	Southeastern Guizhou
SRR23936538	Qiandongnan Xiaoxiang Chicken2	Southeastern Guizhou
SRR23936539	Qiandongnan Xiaoxiang Chicken3	Southeastern Guizhou
SRR23936540	Qiandongnan Xiaoxiang Chicken4	Southeastern Guizhou
SRR23936541	Qiandongnan Xiaoxiang Chicken5	Southeastern Guizhou
SRR23936605	Qiandongnan Xiaoxiang Chicken6	Southeastern Guizhou
SRR23936606	Qiandongnan Xiaoxiang Chicken7	Southeastern Guizhou
SRR23936543	Xingyi Dwarf Chicken1	Xingyi, Guizhou
SRR23936544	Xingyi Dwarf Chicken2	Xingyi, Guizhou
SRR23936545	Xingyi Dwarf Chicken3	Xingyi, Guizhou
SRR23936546	Xingyi Dwarf Chicken4	Xingyi, Guizhou
SRR23936547	Xingyi Dwarf Chicken5	Xingyi, Guizhou
SRR23936548	Xingyi Dwarf Chicken6	Xingyi, Guizhou
SRR23936549	Xingyi Dwarf Chicken7	Xingyi, Guizhou
SRR23936550	Xingyi Dwarf Chicken8	Xingyi, Guizhou
SRR23936551	Xingyi Dwarf Chicken9	Xingyi, Guizhou
SRR23936552	Xingyi Dwarf Chicken10	Xingyi, Guizhou
SRR23936554	Xingyi Dwarf Chicken11	Xingyi, Guizhou
SRR23936555	Xingyi Dwarf Chicken12	Xingyi, Guizhou
SRR23936609	Xingyi Dwarf Chicken13	Xingyi, Guizhou
SRR23936610	Xingyi Dwarf Chicken14	Xingyi, Guizhou
SRR23936592	Puding High-legged Chicken1	Anshun, Guizhou
SRR23936599	Puding High-legged Chicken2	Anshun, Guizhou
SRR23936600	Puding High-legged Chicken3	Anshun, Guizhou
SRR23936601	Puding High-legged Chicken4	Anshun, Guizhou
SRR23936602	Puding High-legged Chicken5	Anshun, Guizhou
SRR23936603	Puding High-legged Chicken6	Anshun, Guizhou
SRR23936668	Puding High-legged Chicken7	Anshun, Guizhou
SRR23936628	Changshun Green-egg Chicken1	Southern Guizhou
SRR23936629	Changshun Green-egg Chicken2	Southern Guizhou

SRR23936630	Changshun Green-egg Chicken3	Southern Guizhou
SRR23936631	Changshun Green-egg Chicken4	Southern Guizhou
SRR23936632	Changshun Green-egg Chicken5	Southern Guizhou
SRR23936681	Changshun Green-egg Chicken6	South-central Guizhou
SRR23936683	Changshun Green-egg Chicken7	South-central Guizhou
SRR23936684	Changshun Green-egg Chicken8	South-central Guizhou
SRR23936685	Changshun Green-egg Chicken9	South-central Guizhou
SRR23936639	Wumeng Black-bone Chicken1	Wumeng, Guizhou
SRR23936641	Wumeng Black-bone Chicken2	Wumeng, Guizhou
SRR23936642	Wumeng Black-bone Chicken3	Wumeng, Guizhou
SRR23936643	Wumeng Black-bone Chicken4	Wumeng, Guizhou
SRR23936644	Wumeng Black-bone Chicken5	Wumeng, Guizhou
SRR23936645	Wumeng Black-bone Chicken6	Wumeng, Guizhou
SRR23936657	Shanghai Xinyang Chicken1	Northern Yunnan
SRR23936658	Shanghai Xinyang Chicken2	Northern Yunnan
SRR23936659	Shanghai Xinyang Chicken3	Northern Yunnan
SRR23936660	Shanghai Xinyang Chicken4	Northern Yunnan
SRR23936661	Shanghai Xinyang Chicken5	Northern Yunnan
SRR23936662	Shanghai Xinyang Chicken6	Northern Yunnan
SRR23936663	Shanghai Xinyang Chicken7	Northern Yunnan
SRR23936534	Shanghai Xinyang Chicken8	Northern Yunnan
SRR23936535	Shanghai Xinyang Chicken9	Northern Yunnan
SRR23936705	Yaoshan Chicken1	Southern Guizhou
SRR23936706	Yaoshan Chicken2	Southern Guizhou
SRR23936707	Yaoshan Chicken3	Southern Guizhou
SRR23936708	Yaoshan Chicken4	Southern Guizhou
SRR23936768	Yaoshan Chicken5	Southern Guizhou
SRR23936769	Yaoshan Chicken6	Southern Guizhou
SRR23936721	Tianfu Broiler Chicken1	Northern Yunnan
SRR23936722	Tianfu Broiler Chicken2	Northern Yunnan
SRR23936723	Tianfu Broiler Chicken3	Northern Yunnan
SRR23936724	Tianfu Broiler Chicken4	Northern Yunnan
SRR23936725	Tianfu Broiler Chicken5	Northern Yunnan
SRR23936726	Tianfu Broiler Chicken6	Northern Yunnan
SRR23936727	Tianfu Broiler Chicken7	Northern Yunnan
SRR23936728	Tianfu Broiler Chicken8	Northern Yunnan
SRR23936729	Tianfu Broiler Chicken9	Northern Yunnan
SRR23936581	Tianfu Broiler Chicken10	Northern Yunnan
SRR23936582	Tianfu Broiler Chicken11	Northern Yunnan
SRR23936583	Tianfu Broiler Chicken12	Northern Yunnan
SRR23936793	Chishui Black-bone Chicken1	Chishui, Guizhou
SRR23936794	Chishui Black-bone Chicken2	Chishui, Guizhou
SRR23936795	Chishui Black-bone Chicken3	Chishui, Guizhou

SRR23936796	Chishui Black-bone Chicken4	Chishui, Guizhou
SRR23936797	Chishui Black-bone Chicken5	Chishui, Guizhou
SRR23936799	Chishui Black-bone Chicken6	Chishui, Guizhou
SRR23936804	Chishui Black-bone Chicken7	Chishui, Guizhou
SRR23936805	Chishui Black-bone Chicken8	Chishui, Guizhou
SRR23936806	Chishui Black-bone Chicken9	Chishui, Guizhou
SRR23936807	Chishui Black-bone Chicken10	Chishui, Guizhou
SRR23936808	Chishui Black-bone Chicken11	Chishui, Guizhou
SRR23936810	Chishui Black-bone Chicken12	Chishui, Guizhou
SRR23936811	Chishui Black-bone Chicken13	Chishui, Guizhou
SRR23936812	Chishui Black-bone Chicken14	Chishui, Guizhou
SRR24423387	Tibetan black chicken1	Lhunze County, Shannan, Xizang 3900m
SRR24423388	Tibetan black chicken2	Lhunze County, Shannan, Xizang 3900m
SRR24423389	Tibetan black chicken3	Lhunze County, Shannan, Xizang 3900m
SRR24423390	Tibetan black chicken4	Lhunze County, Shannan, Xizang 3900m
SRR24423391	Tibetan black chicken5	Lhunze County, Shannan, Xizang 3900m
SRR24423392	Tibetan black chicken6	Lhunze County, Shannan, Xizang 3900m
SRR24423393	Tibetan black chicken7	Lhunze County, Shannan, Xizang 3900m
SRR24423394	Tibetan black chicken8	Lhunze County, Shannan, Xizang 3900m
SRR24423395	Tibetan black chicken9	Lhunze County, Shannan, Xizang 3900m
SRR24423396	Tibetan black chicken10	Lhunze County, Shannan, Xizang 3900m
SRR24423397	Tibetan black chicken11	Lhunze County, Shannan, Xizang 3900m
SRR24423398	Tibetan black chicken12	Lhunze County, Shannan, Xizang 3900m
SRR24423399	Tibetan black chicken13	Lhunze County, Shannan, Xizang 3900m
SRR24423400	Tibetan black chicken14	Lhunze County, Shannan, Xizang 3900m
SRR24423401	Tibetan black chicken15	Lhunze County, Shannan, Xizang 3900m
SRR5149619	Sichuan indigenous chicken	Ya'an, Sichuan
SRR851160	Silkie1	Taiwan, China

SRR867742	Silkie2	Taiwan, China
SRR867743	Silkie3	Taiwan, China
SRR867744	Silkie4	Taiwan, China
SRR867746	Silkie5	Taiwan, China
SRR867747	Silkie6	Taiwan, China
SRR867748	Taiwanese native chicken 1	Taiwan, China
SRR867749	Taiwanese native chicken 2	Taiwan, China
SRR867750	Taiwanese native chicken 3	Taiwan, China
SRR867767	Taiwanese native chicken 4	Taiwan, China
SRR867768	Taiwanese native chicken 5	Taiwan, China
SRR8730633	North American Araucana1	United States
SRR8730634	North American Araucana2	United States
SRR8730635	North American Araucana3	United States
SRR8730636	North American Araucana4	United States
SRR8730637	North American Araucana5	United States
SRR8730638	North American Araucana6	United States
